# Supplementary material for: Synthesis and Characterization of Novel Methyl (3)5-(N-Boc-piperidinyl)-1H-pyrazole-4-carboxylates
Source: Molecules. 2021 Jun 22;26(13):3808. doi: 10.3390/molecules26133808 (PMC8270337; doi:10.3390/molecules26133808)
Supplement: Supplementary file 1 [file molecules-26-03808-s001.zip › molecules-1244469-supplementary.pdf]

Supplementary Materials

Synthesis and Characterization of Novel Methyl (3)5-(*N*-Boc-piperidinyl)-1*H*-pyrazole-4-carboxylates

Gita Matulevičiūtė [1,2], Eglė Arbačiauskienė [2,*], Neringa Kleizienė [1], Vilija Kederienė [2], Greta Ragaitė [1], Miglė Dagilienė [1], Aurimas Bieliauskas [1], Vaida Milišiūnaitė [1], Frank A. Sløk [3] and Algirdas Šačkus [1,*]

[1] Institute of Synthetic Chemistry, Kaunas University of Technology,
K. Baršausko g. 59, Kaunas LT-51423, Lithuania;

[2] Department of Organic Chemistry, Kaunas University of Technology,
Radvilenų pl. 19, Kaunas LT-50254, Lithuania;

[3] Vipergen ApS,
Gammel Kongevej 23A, Copenhagen V DK-1610, Denmark

* Corresponding authors

E-mail address: algirdas.sackus@ktu.lt (A. Šačkus), egle.arbaciauskiene@ktu.lt (E. Arbačiauskienė)

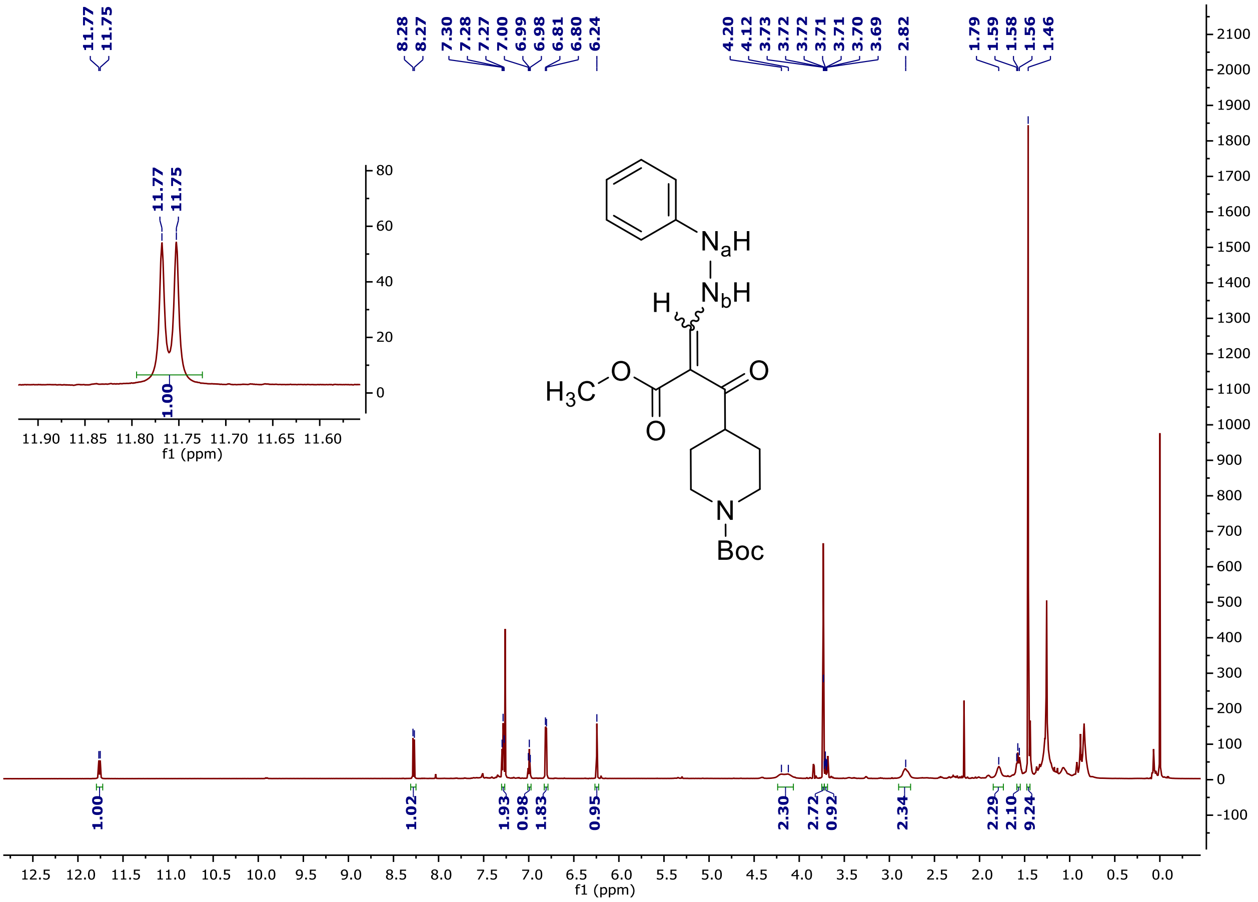

Figure S1. ***tert*-Butyl 4-[(2*E(Z)*)-2-(methoxycarbonyl)-3-(2-phenylhydrazinyl)prop-2-enoyl]piperidine-1-carboxylate (4a). ^{1}H NMR spectrum (700 MHz, CDCl$_3$).**

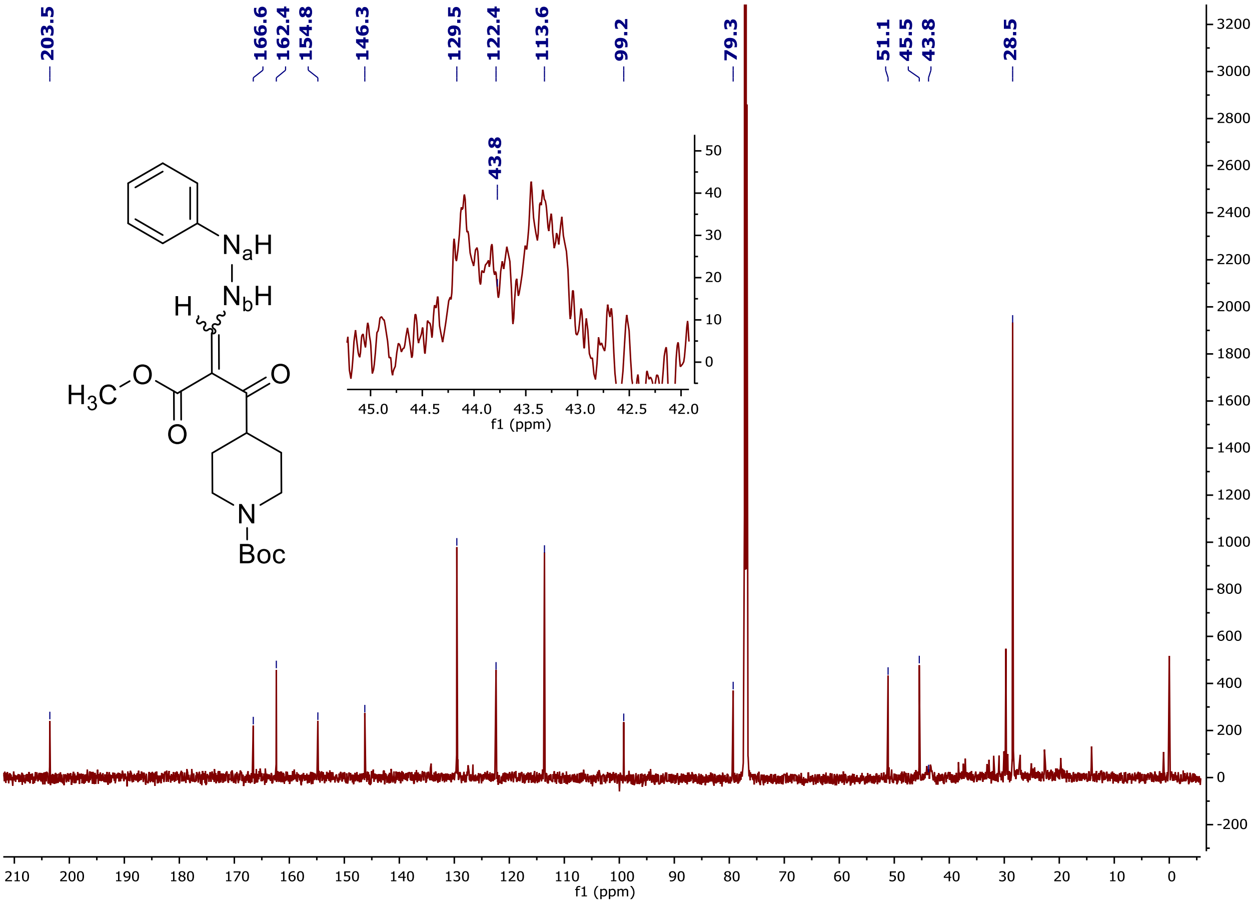

Figure S2. ***tert*-Butyl 4-[(2*E(Z)*)-2-(methoxycarbonyl)-3-(2-phenylhydrazinyl)prop-2-enoyl]piperidine-1-carboxylate (4a). ^{13}C NMR spectrum (176 MHz, CDCl$_3$).**

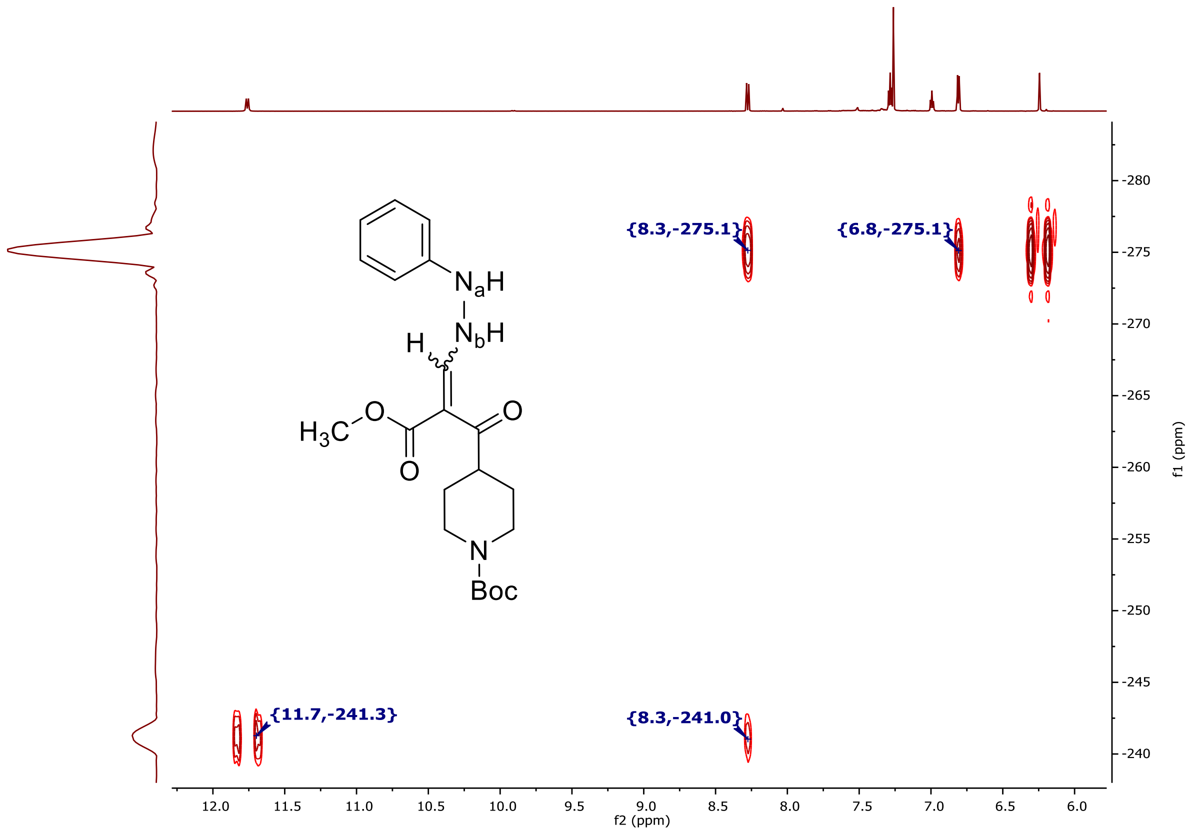

Figure S3. ***tert*-Butyl 4-[(2*E(Z)*)-2-(methoxycarbonyl)-3-(2-phenylhydrazinyl)prop-2-enoyl]piperidine-1-carboxylate (4a). ^{1}H-^{15}N HMBC spectrum (71 MHz, $CDCl_3$).**

Compound Spectrum SmartFormula Report

Analysis Info

Analysis Name	D:\Data\GMP-601-E_tarpinis.d	Acquisition Date	12/5/2020 1:48:04 AM
Method	DirectInfusion_TuneLow_pos.m	Operator	hplc
Sample Name	GMP-601-E_tarpinis	Instrument	micrOTOF-Q III 8228888.20448
Comment	AB		

Acquisition Parameter

Source Type	ESI	Ion Polarity	Positive	Set Nebulizer	0.4 Bar
Focus	Not active	Set Capillary	4500 V	Set Dry Heater	180 °C
Scan Begin	50 m/z	Set End Plate Offset	-500 V	Set Dry Gas	4.0 l/min
Scan End	1000 m/z	Set Collision Cell RF	140.0 Vpp	Set Divert Valve	Waste

#	RT [min]	Area	Int. Type	I	S/N	Chromatogram	Max. m/z	FWHM [min]
n.a.	9.3	n.a.	Single spectrum	n.a.	n.a.	n.a.	426.2001	n.a.

+MS, 9.3min #555

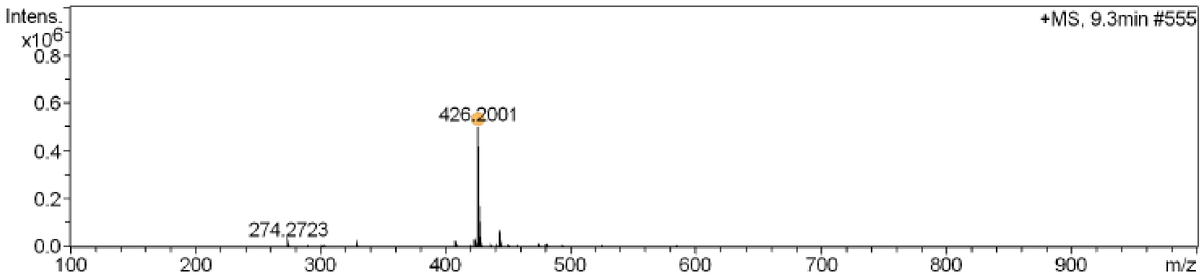

Meas. m/z	#	Ion Formula	m/z	err [ppm]	mSigma	# Sigma	Score	rdb	e¯ Conf	N-Rule
426.2001	1	C21H29N3NaO5	426.1999	-0.5	7.3	1	100.00	8.5	even	ok

Figure S4. *tert*-Butyl 4-[(2*E(Z)*)-2-(methoxycarbonyl)-3-(2-phenylhydrazinyl)prop-2-enoyl]piperidine-1-carboxylate (4a). HRMS (ESI-TOF).

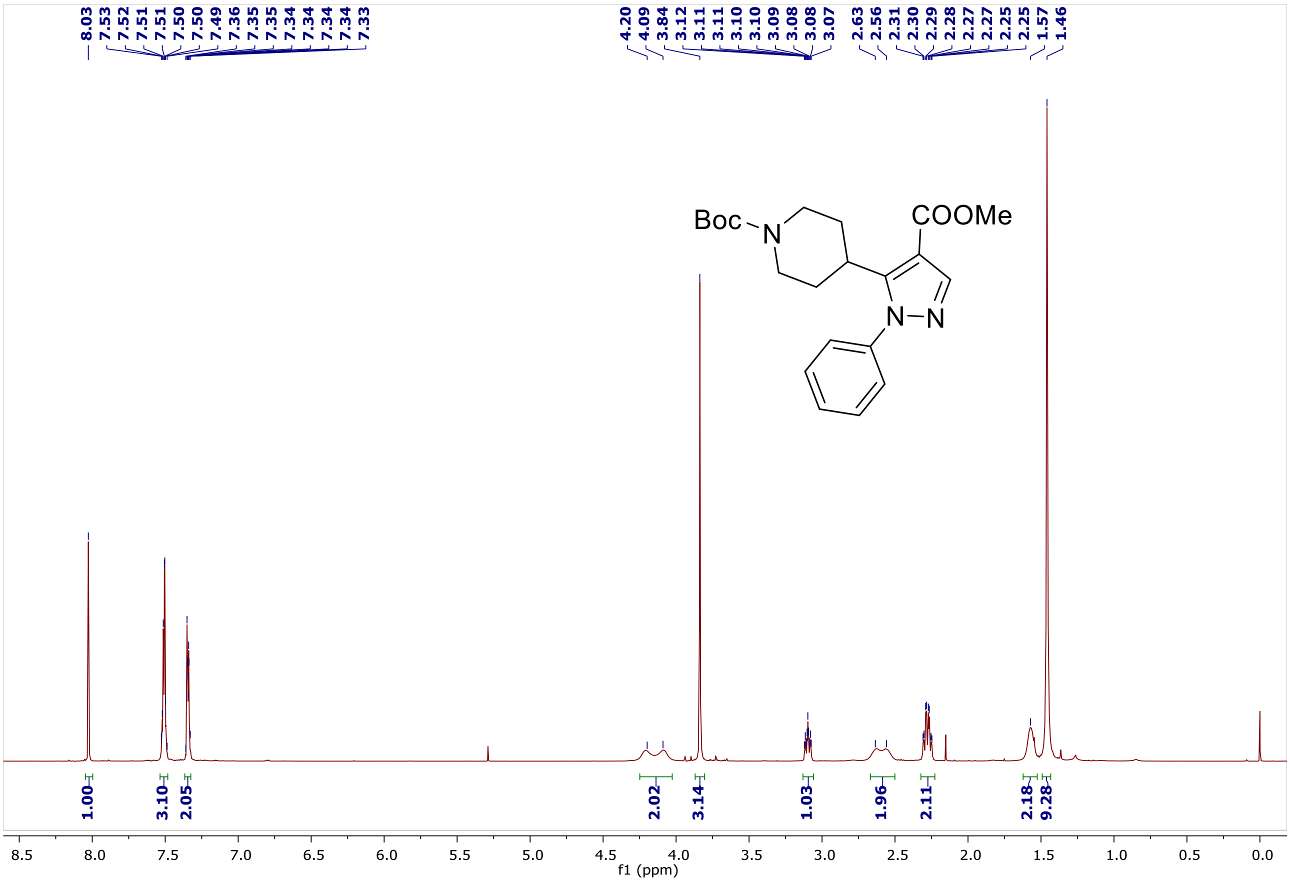

Figure S5. ***tert*-Butyl 4-[4-(methoxycarbonyl)-1-phenyl-1*H*-pyrazol-5-yl]piperidine-1-carboxylate (5a). ^{1}H NMR spectrum (700 MHz, $CDCl_3$).**

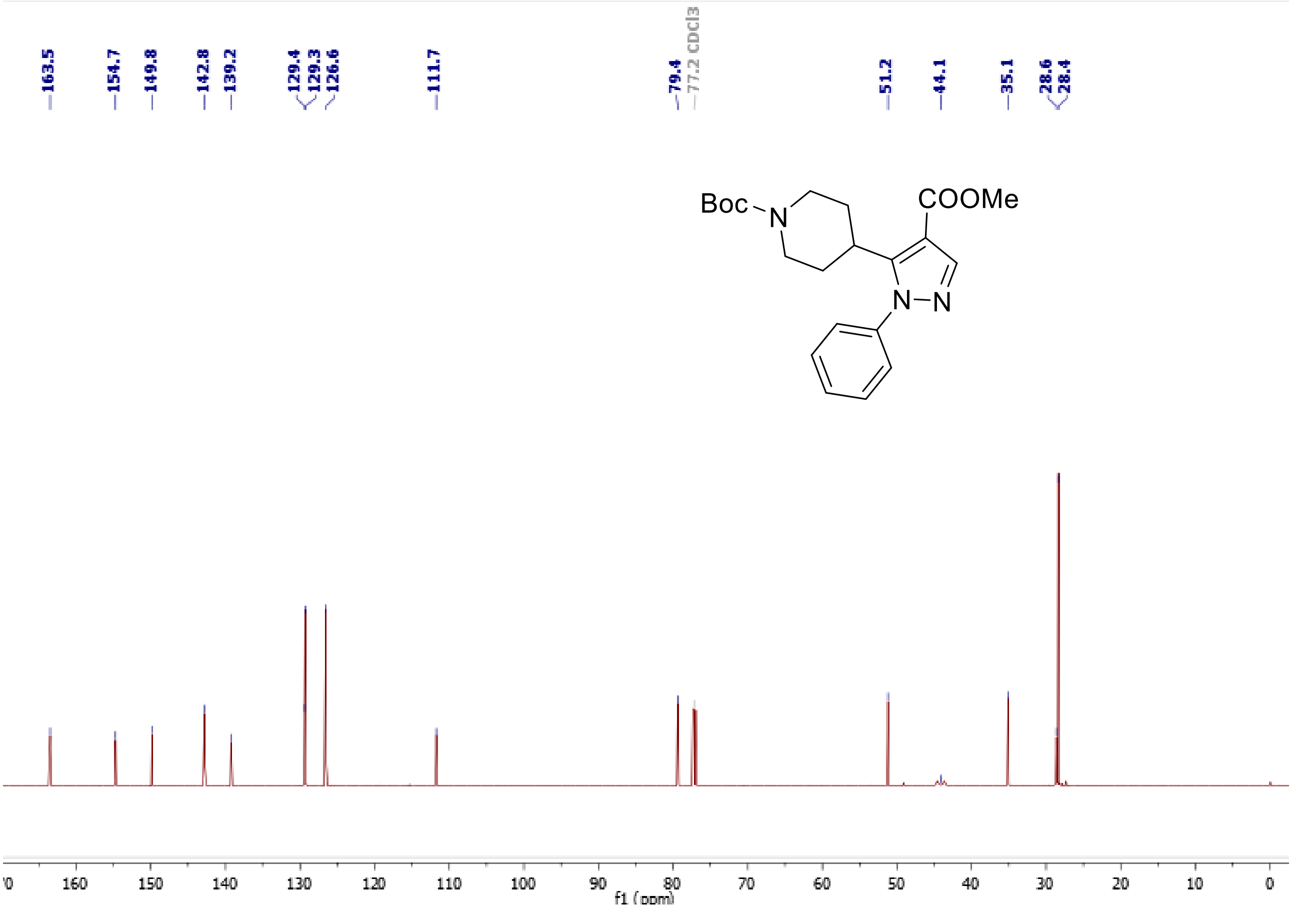

Figure S6. ***tert*-Butyl 4-[4-(methoxycarbonyl)-1-phenyl-1*H*-pyrazol-5-yl]piperidine-1-carboxylate (5a). ^{13}C NMR spectrum (176 MHz, $CDCl_3$).**

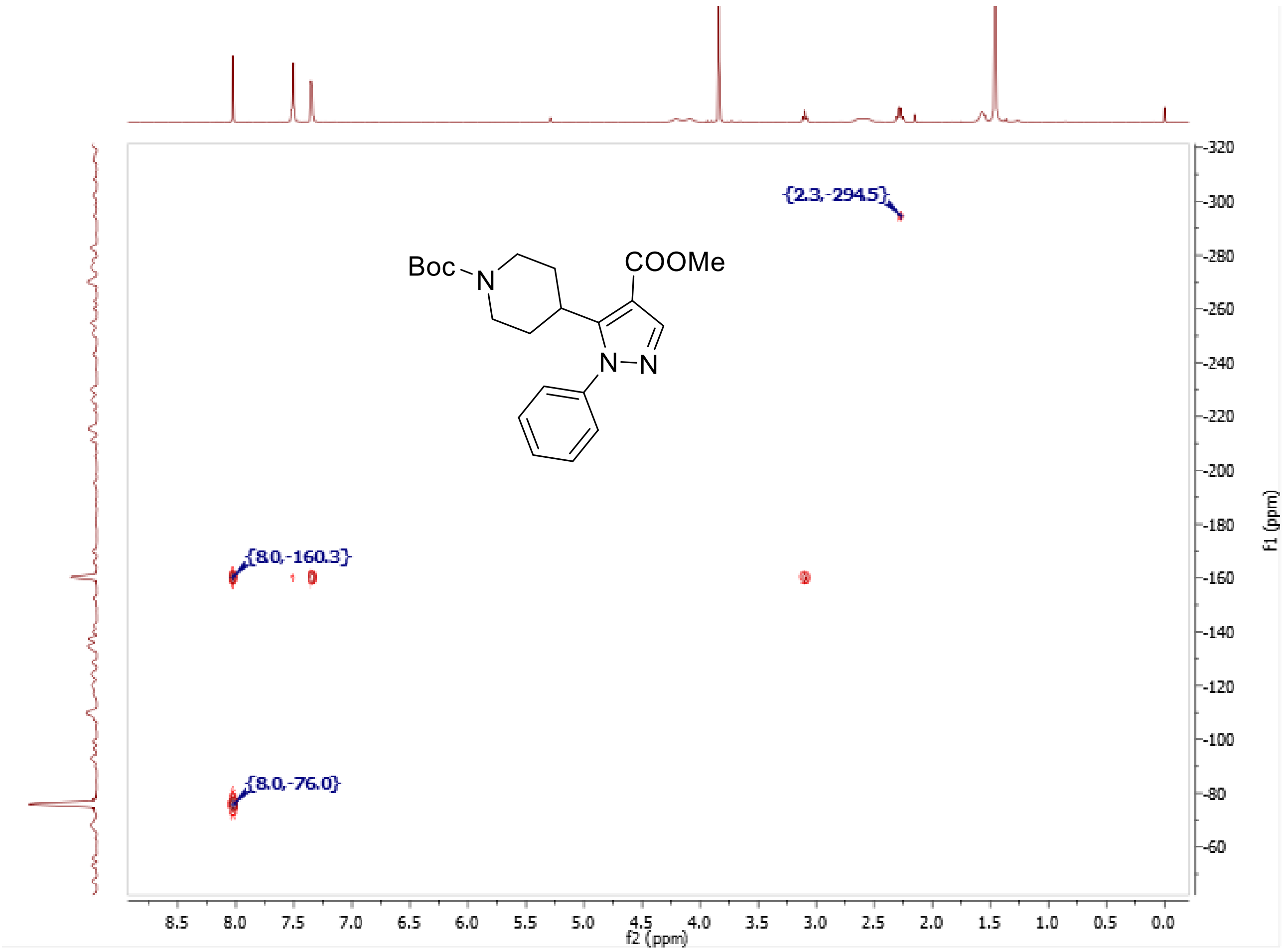

Figure S7. ***tert*****-Butyl 4-[4-(methoxycarbonyl)-1-phenyl-1*H*-pyrazol-5-yl]piperidine-1-carboxylate (5a). ^{1}H-^{15}N HMBC spectrum (71 MHz, $CDCl_3$).**

Compound Spectrum SmartFormula Report

Analysis Info

Analysis Name	D:\Data\GMA-021.d	Acquisition Date	11/27/2018 11:59:44 AM
Method	DirectInfusion_TuneLow_pos.m	Operator	hplc
Sample Name	GMA-021	Instrument	micrOTOF-Q III 8228888.20448
Comment	SB		

Acquisition Parameter

Source Type	ESI	Ion Polarity	Positive	Set Nebulizer	0.4 Bar
Focus	Not active	Set Capillary	4500 V	Set Dry Heater	180 °C
Scan Begin	50 m/z	Set End Plate Offset	-500 V	Set Dry Gas	4.0 l/min
Scan End	1000 m/z	Set Collision Cell RF	140.0 Vpp	Set Divert Valve	Waste

#	RT [min]	Area	Int. Type	I	S/N	Chromatogram	Max. m/z	FWHM [min]
n.a.	0.2	n.a.	Single spectrum	n.a.	n.a.	n.a.	304.2632	n.a.
n.a.	4.1	n.a.	Single spectrum	n.a.	n.a.	n.a.	408.1894	n.a.

+MS, 4.1min #246

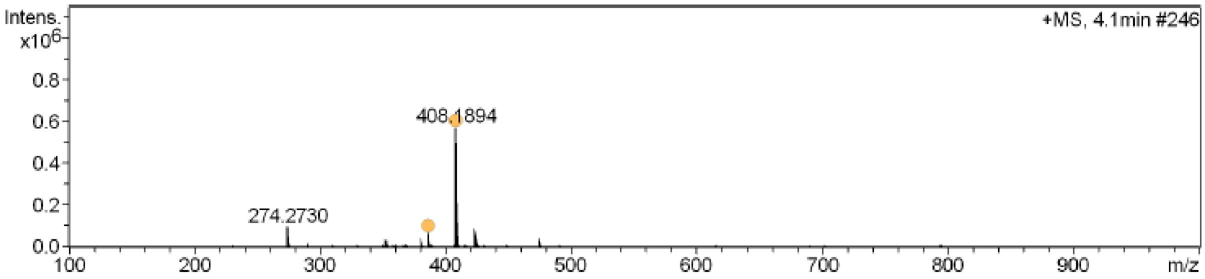

Meas. m/z	#	Ion Formula	m/z	err [ppm]	mSigma	# Sigma	Score	rdb	e⁻ Conf	N-Rule
386.2075	1	C21H28N3O4	386.2074	-0.2	5.8	1	100.00	9.5	even	ok
408.1894	1	C21H27N3NaO4	408.1894	0.1	1.4	1	100.00	9.5	even	ok

Figure S8. *tert*-Butyl 4-[4-(methoxycarbonyl)-1-phenyl-1*H*-pyrazol-5-yl]piperidine-1-carboxylate (5a). HRMS (ESI-TOF).

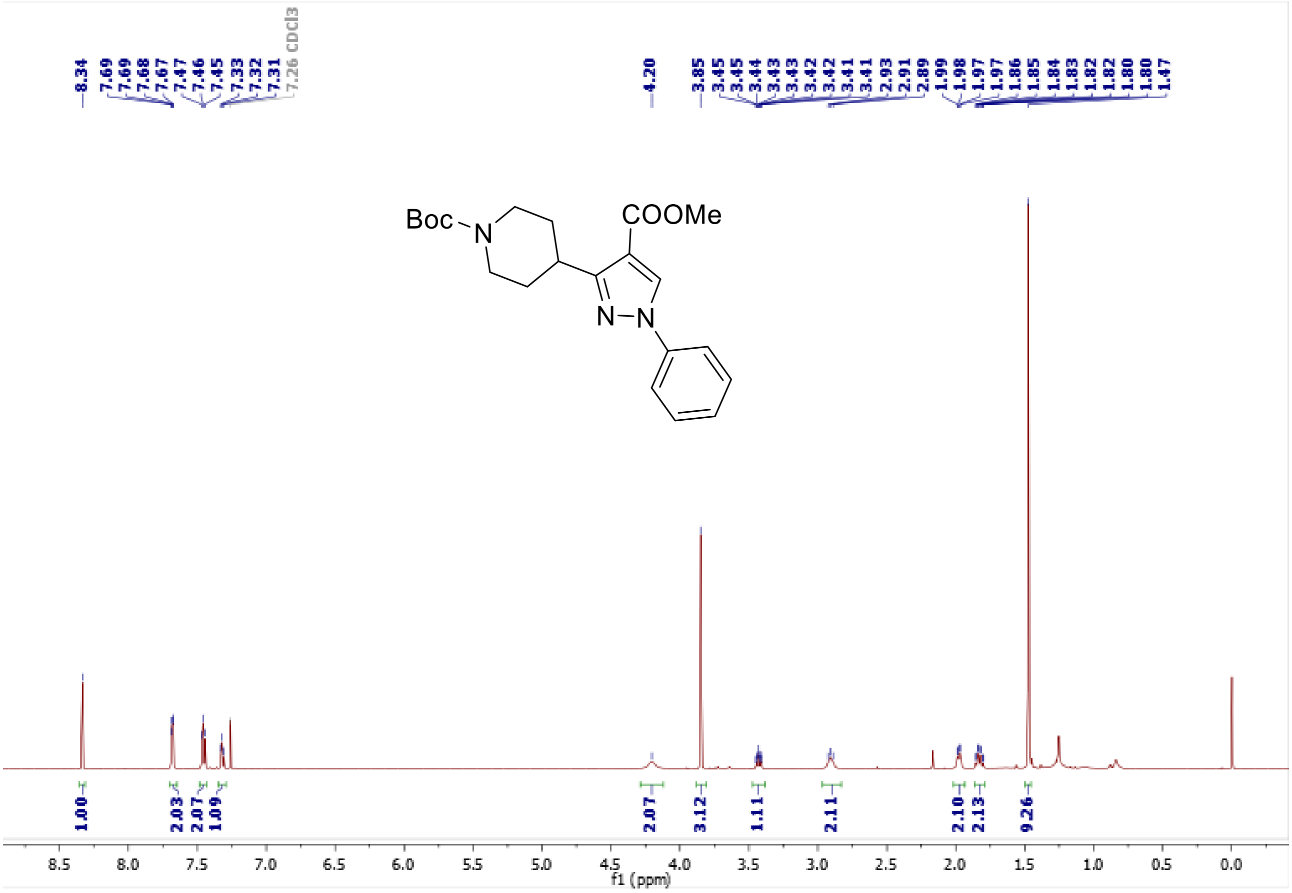

Figure S9. ***tert*-Butyl 4-[4-(methoxycarbonyl)-1-phenyl-1*H*-pyrazol-3-yl]piperidine-1-carboxylate (6a). ^{1}H NMR spectrum (700 MHz, $CDCl_3$).**

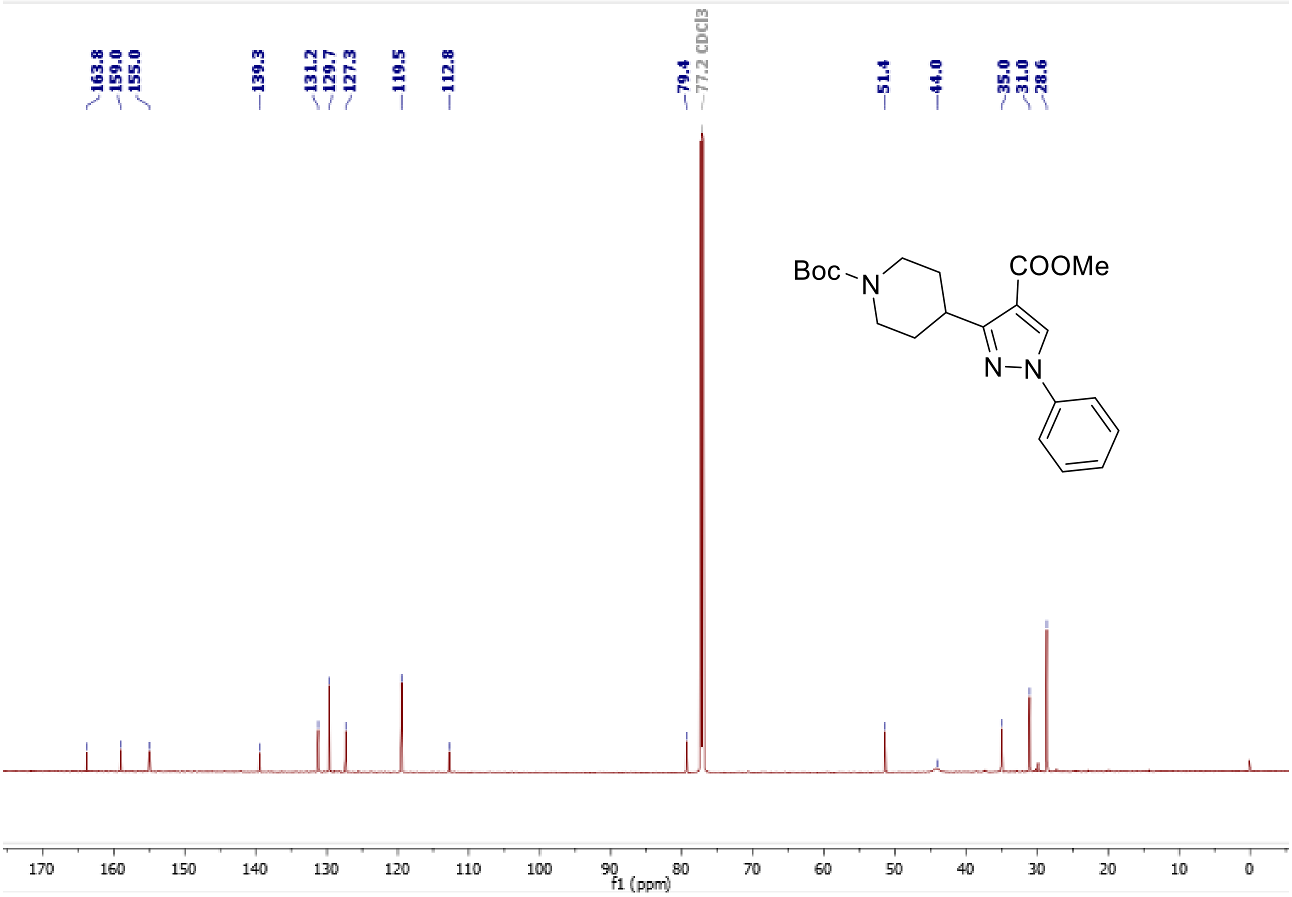

Figure S10. ***tert*-Butyl 4-[4-(methoxycarbonyl)-1-phenyl-1*H*-pyrazol-3-yl]piperidine-1-carboxylate (6a). ^{13}C NMR spectrum (176 MHz, $CDCl_3$).**

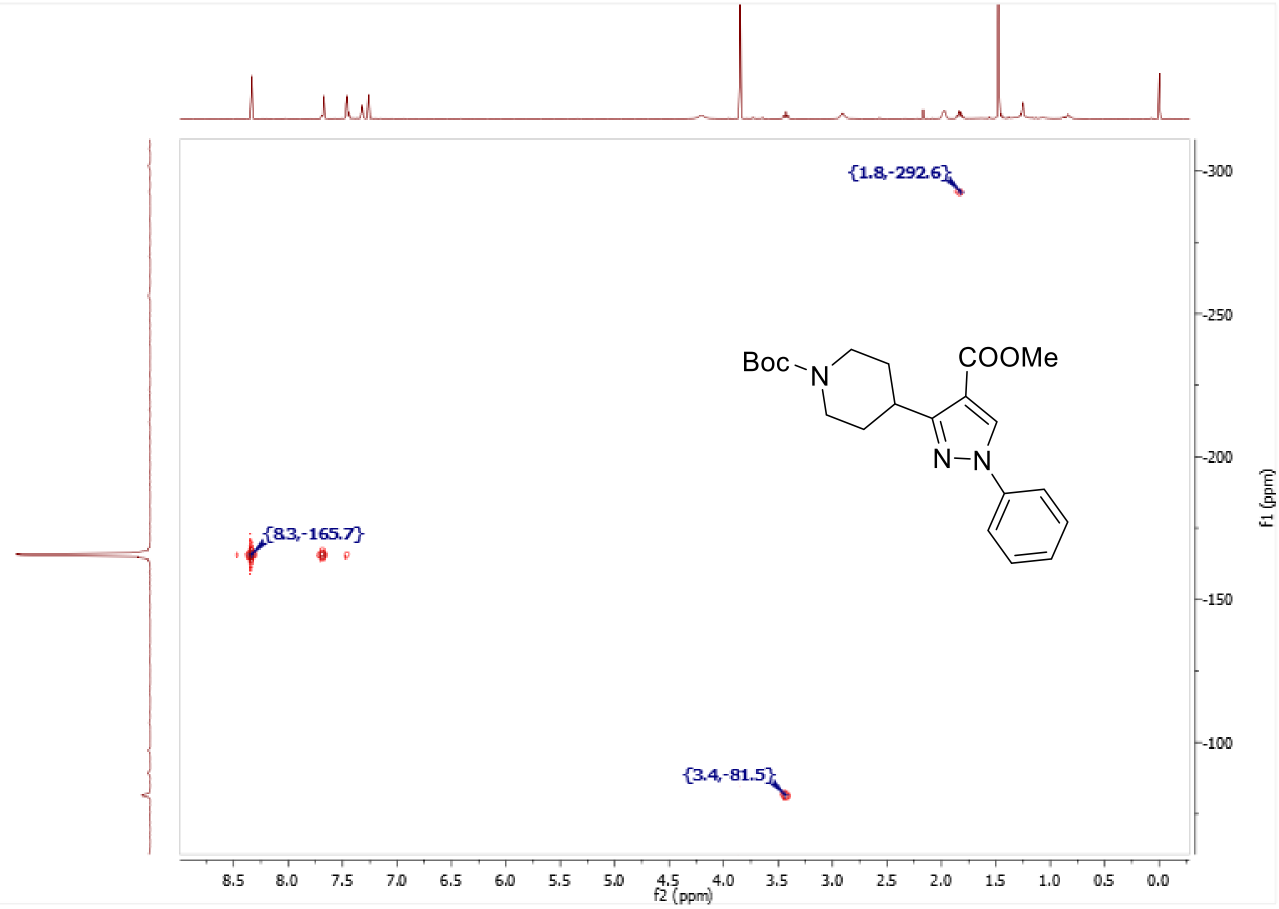

Figure S11. ***tert*-Butyl 4-[4-(methoxycarbonyl)-1-phenyl-1*H*-pyrazol-3-yl]piperidine-1-carboxylate (6a). ^{1}H-^{15}N HMBC spectrum (71 MHz, $CDCl_3$).**

Compound Spectrum SmartFormula Report

Analysis Info

Analysis Name	D:\Data\GMP-601-E1.d	Acquisition Date	2/4/2021 11:29:42 PM
Method	DirectInfusion_TuneLow_pos.m	Operator	hplc
Sample Name	GMP-601-E1	Instrument	micrOTOF-Q III 8228888.20448
Comment	AB		

Acquisition Parameter

Source Type	ESI	Ion Polarity	Positive	Set Nebulizer	0.4 Bar
Focus	Not active	Set Capillary	4500 V	Set Dry Heater	180 °C
Scan Begin	50 m/z	Set End Plate Offset	-500 V	Set Dry Gas	4.0 l/min
Scan End	1000 m/z	Set Collision Cell RF	140.0 Vpp	Set Divert Valve	Waste

#	RT [min]	Area	Int. Type	I	S/N	Chromatogram	Max. m/z	FWHM [min]
n.a.	2.1	n.a.	Single spectrum	n.a.	n.a.	n.a.	408.1894	n.a.

+MS, 2.1min #125

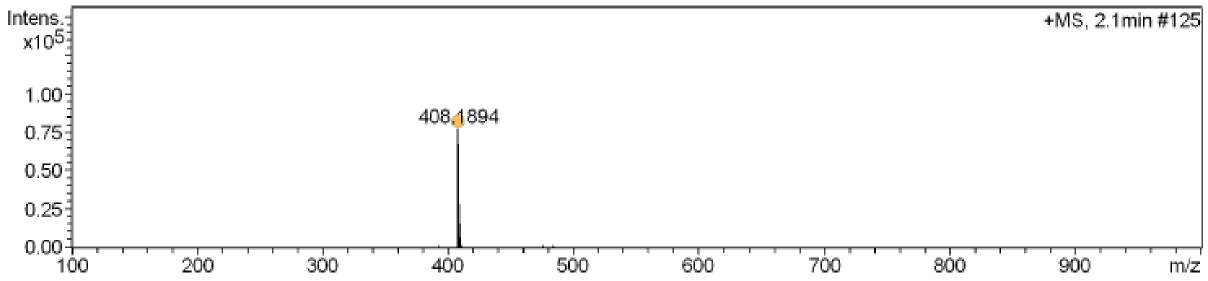

Meas. m/z	#	Ion Formula	m/z	err [ppm]	mSigma	# Sigma	Score	rdb	e¯ Conf	N-Rule
408.1894	1	C21H27N3NaO4	408.1894	-0.1	5.8	1	100.00	9.5	even	ok

Figure S12. *tert*-Butyl 4-[4-(methoxycarbonyl)-1-phenyl-1*H*-pyrazol-3-yl]piperidine-1-carboxylate (6a). HRMS (ESI-TOF).

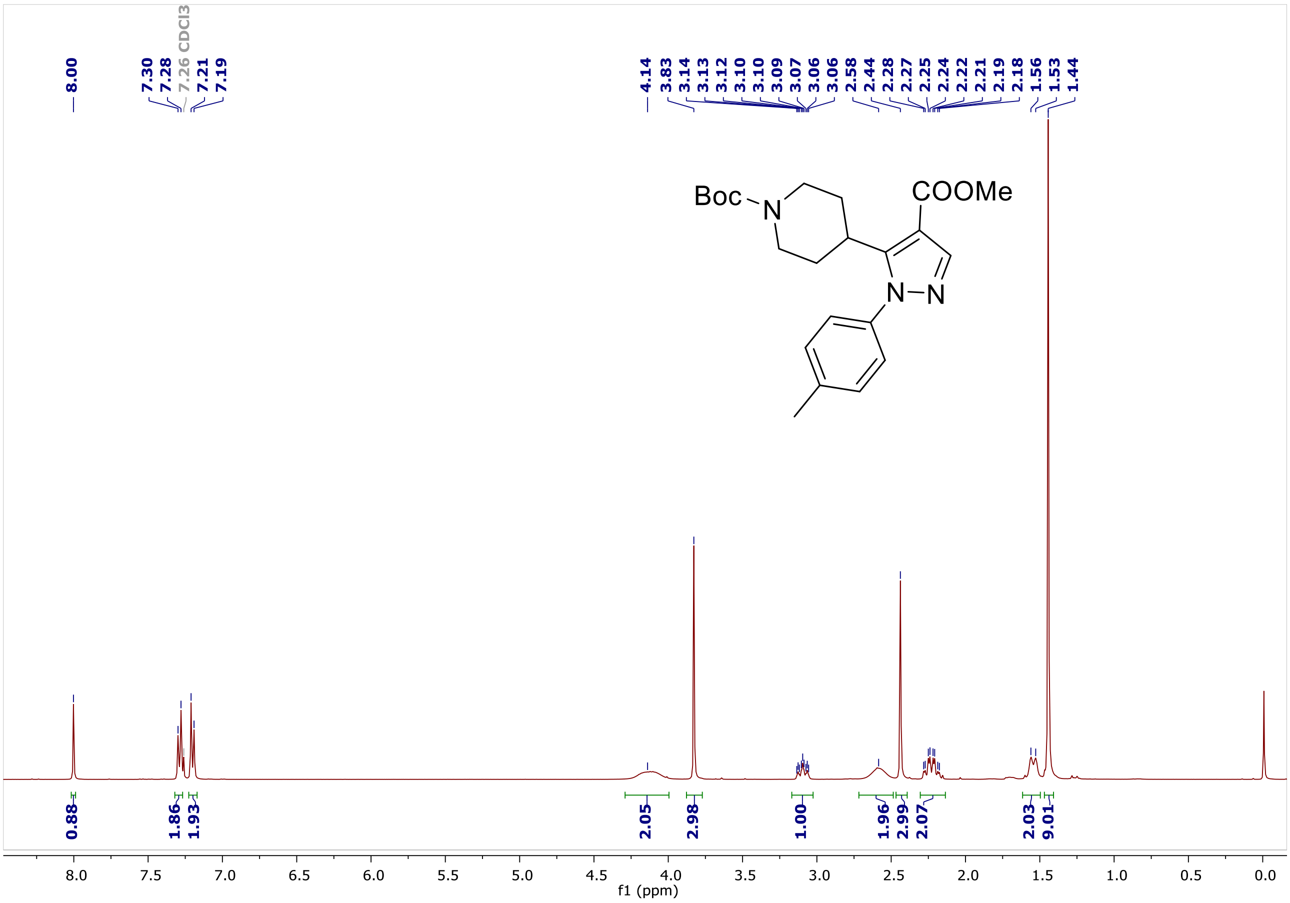

Figure S13. ***tert*-Butyl 4-[4-(methoxycarbonyl)-1-(4-methylphenyl)-1*H*-pyrazol-5-yl]piperidine-1-carboxylate (5b). ^{1}H NMR spectrum (400 MHz, $CDCl_3$).**

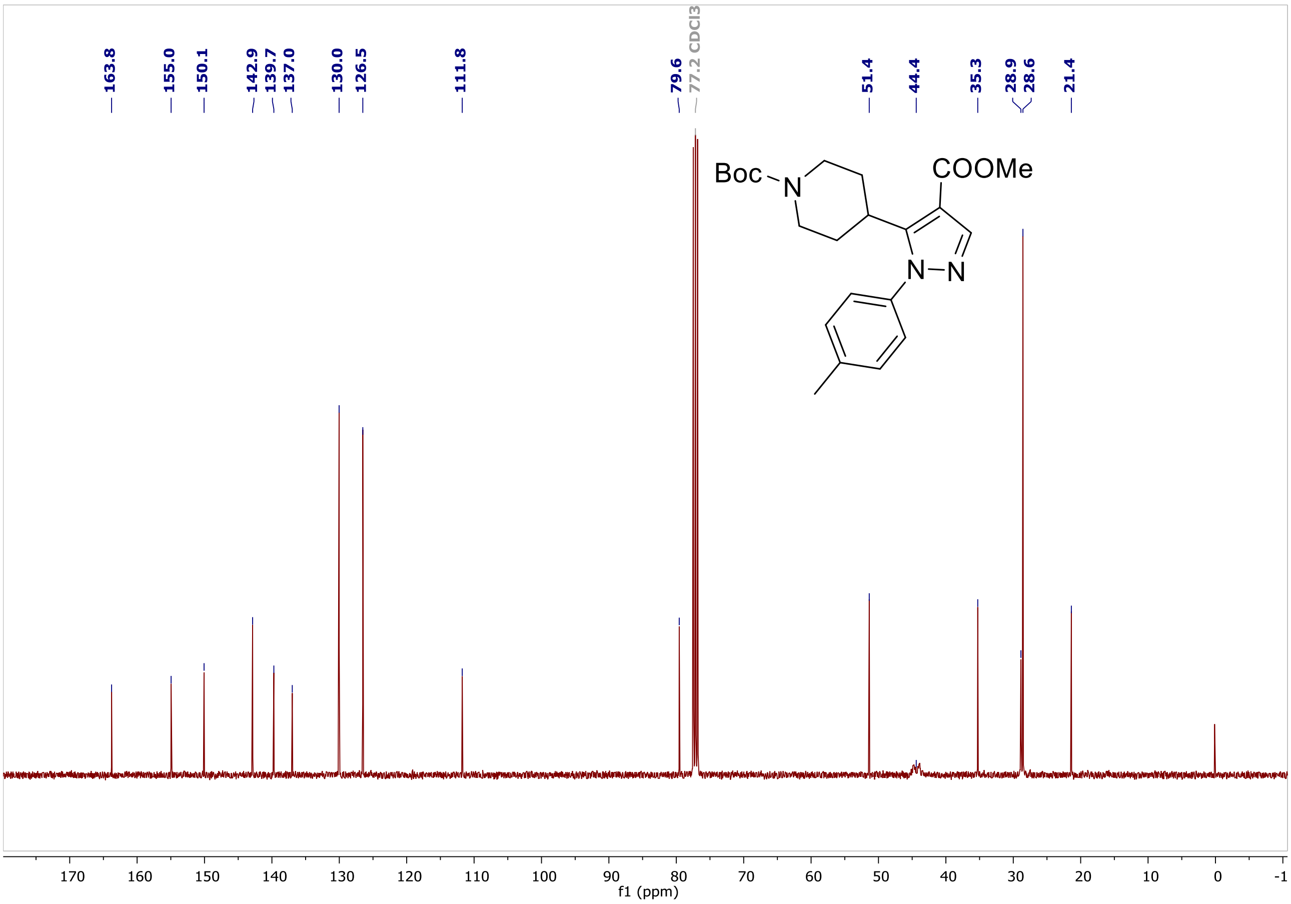

Figure S14. ***tert*-Butyl 4-[4-(methoxycarbonyl)-1-(4-methylphenyl)-1*H*-pyrazol-5-yl]piperidine-1-carboxylate (5b). ^{13}C NMR spectrum (101 MHz, $CDCl_3$).**

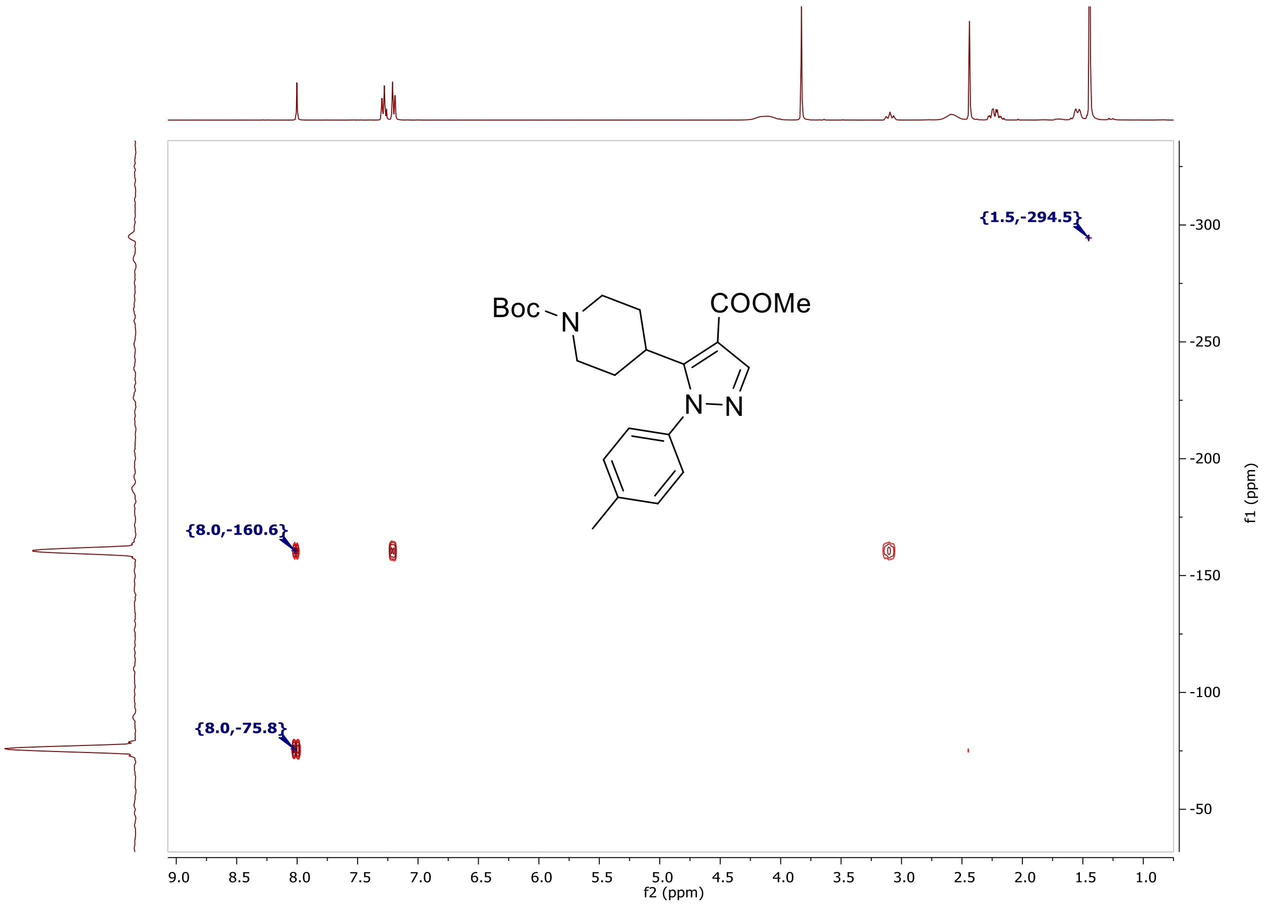

Figure S15. *tert*-Butyl 4-[4-(methoxycarbonyl)-1-(4-methylphenyl)-1*H*-pyrazol-5-yl]piperidine-1-carboxylate (5b). ^{1}H-^{15}N HMBC spectrum (41 MHz, $CDCl_3$).

Compound Spectrum SmartFormula Report

Analysis Info

Analysis Name	D:\Data\GMP-007.d	Acquisition Date	11/27/2018 11:44:50 AM
Method	DirectInfusion_TuneLow_pos.m	Operator	hplc
Sample Name	GMP-007	Instrument	micrOTOF-Q III 8228888.20448
Comment	SB		

Acquisition Parameter

Source Type	ESI	Ion Polarity	Positive	Set Nebulizer	0.4 Bar
Focus	Not active	Set Capillary	4500 V	Set Dry Heater	180 °C
Scan Begin	50 m/z	Set End Plate Offset	-500 V	Set Dry Gas	4.0 l/min
Scan End	1000 m/z	Set Collision Cell RF	140.0 Vpp	Set Divert Valve	Waste

#	RT [min]	Area	Int. Type	I	S/N	Chromatogram	Max. m/z	FWHM [min]
n.a.	0.2	n.a.	Single spectrum	n.a.	n.a.	n.a.	304.2634	n.a.
n.a.	3.7	n.a.	Single spectrum	n.a.	n.a.	n.a.	422.2051	n.a.

+MS, 3.7min #221

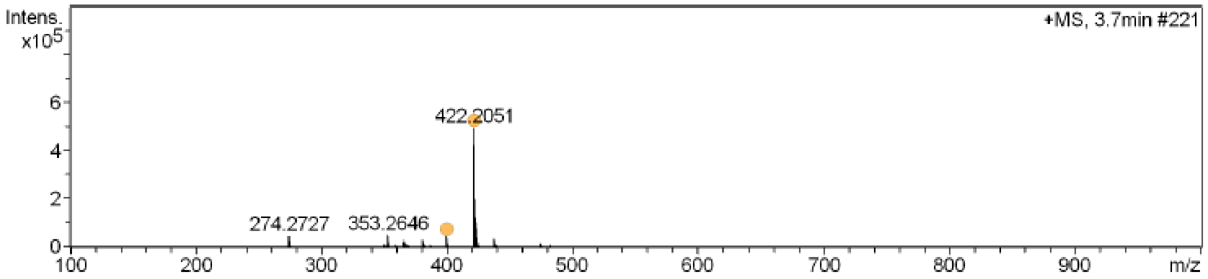

Meas. m/z	#	Ion Formula	m/z	err [ppm]	mSigma	# Sigma	Score	rdb	e⁻ Conf	N-Rule
400.2231	1	C22H30N3O4	400.2231	-0.0	8.7	1	100.00	9.5	even	ok
422.2051	1	C22H29N3NaO4	422.2050	-0.2	2.1	1	100.00	9.5	even	ok

Figure S16. *tert*-Butyl 4-[4-(methoxycarbonyl)-1-(4-methylphenyl)-1*H*-pyrazol-5-yl]piperidine-1-carboxylate (5b). HRMS (ESI-TOF).

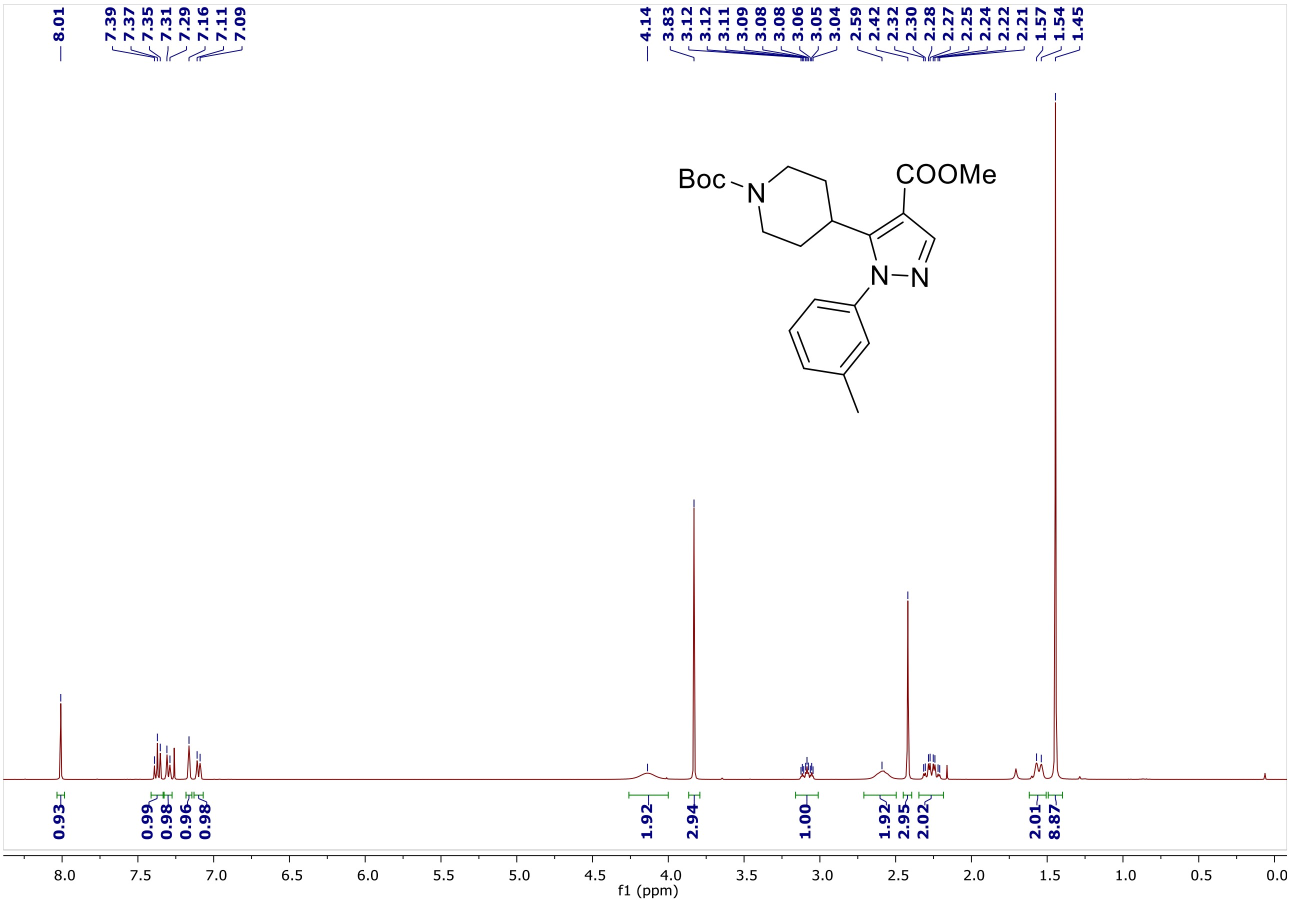

Figure S17. ***tert*****-Butyl 4-[4-(methoxycarbonyl)-1-(3-methylphenyl)-1*****H*****-pyrazol-5-yl]piperidine-1-carboxylate (5c). ^{1}H NMR spectrum (400 MHz, CDCl$_3$).**

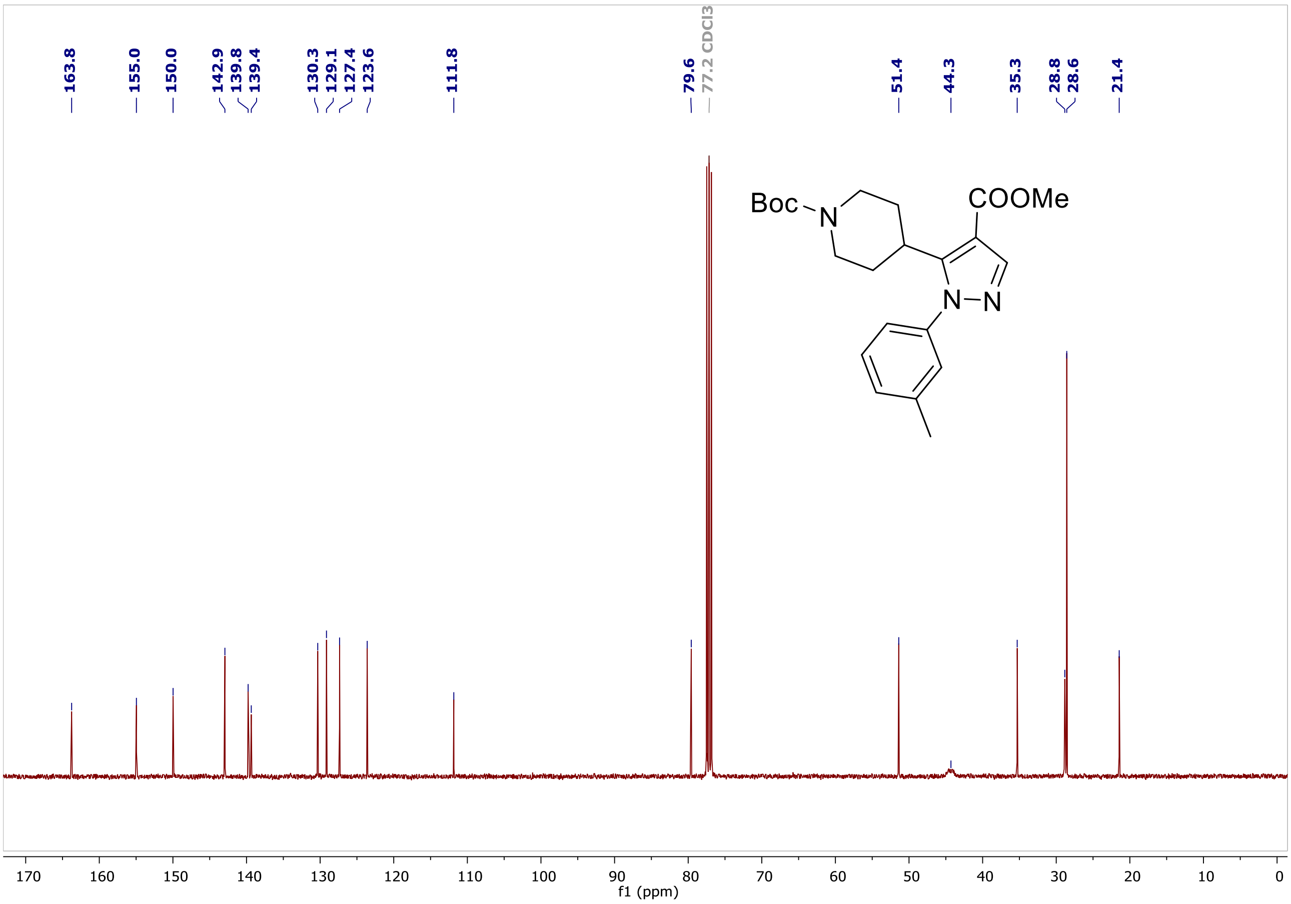

Figure S18. ***tert*****-Butyl 4-[4-(methoxycarbonyl)-1-(3-methylphenyl)-1*****H*****-pyrazol-5-yl]piperidine-1-carboxylate (5c). ^{13}C NMR spectrum (176 MHz, CDCl$_3$).**

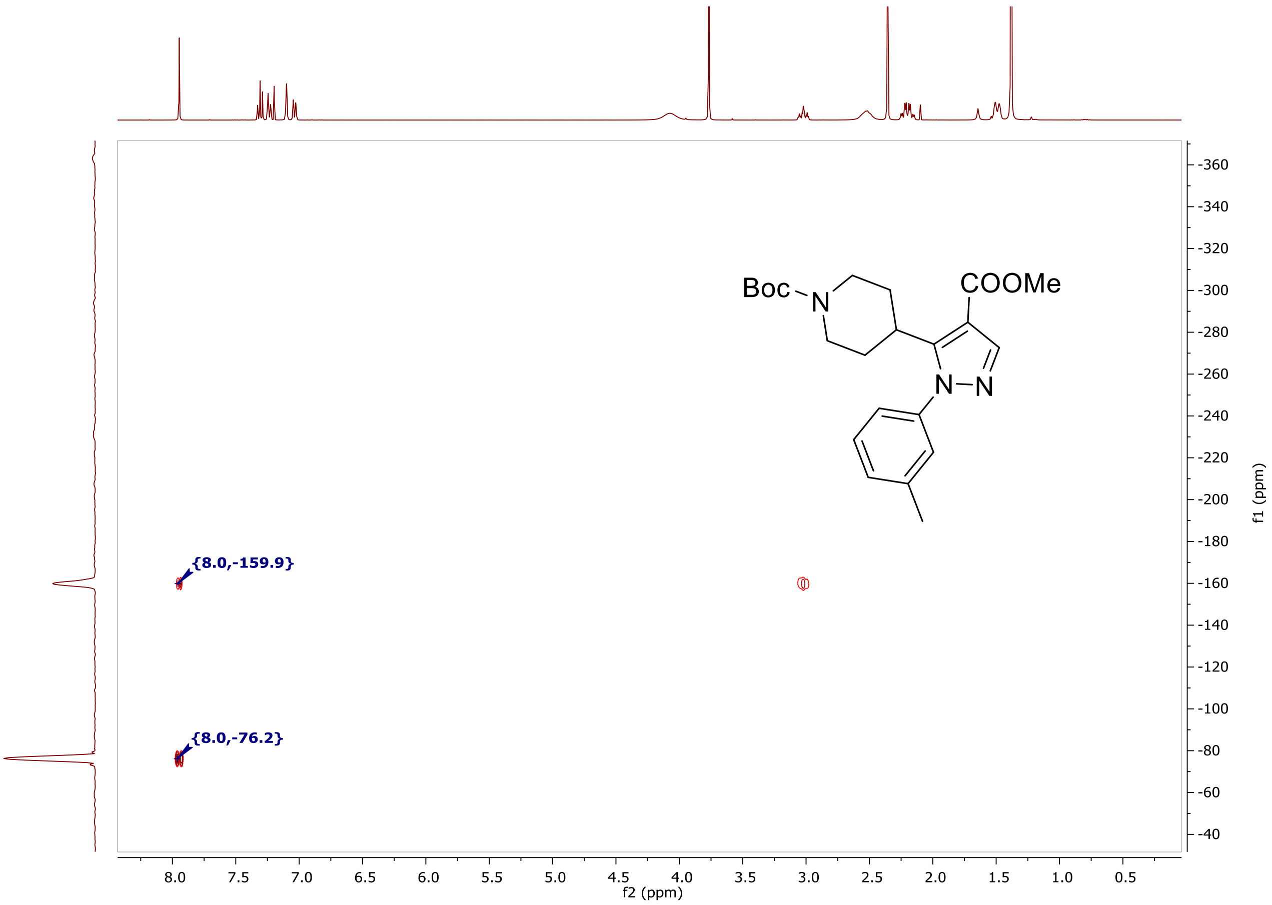

Figure S19. ***tert*-Butyl 4-[4-(methoxycarbonyl)-1-(3-methylphenyl)-1*H*-pyrazol-5-yl]piperidine-1-carboxylate (5c). ^{15}N NMR spectrum (41 MHz, $CDCl_3$).**

Compound Spectrum SmartFormula Report

Analysis Info

Analysis Name	D:\Data\GMP-039.d	Acquisition Date	11/29/2018 12:15:34 PM
Method	DirectInfusion_TuneLow_pos.m	Operator	hplc
Sample Name	GMP-039	Instrument	micrOTOF-Q III 8228888.20448
Comment	SB		

Acquisition Parameter

Source Type	ESI	Ion Polarity	Positive	Set Nebulizer	0.4 Bar
Focus	Not active	Set Capillary	4500 V	Set Dry Heater	180 °C
Scan Begin	50 m/z	Set End Plate Offset	-500 V	Set Dry Gas	4.0 l/min
Scan End	1000 m/z	Set Collision Cell RF	140.0 Vpp	Set Divert Valve	Waste

#	RT [min]	Area	Int. Type	I	S/N	Chromatogram	Max. m/z	FWHM [min]
n.a.	0.8	n.a.	Single spectrum	n.a.	n.a.	n.a.	226.9519	n.a.
n.a.	3.6	n.a.	Single spectrum	n.a.	n.a.	n.a.	422.2050	n.a.

+MS, 3.6min #216

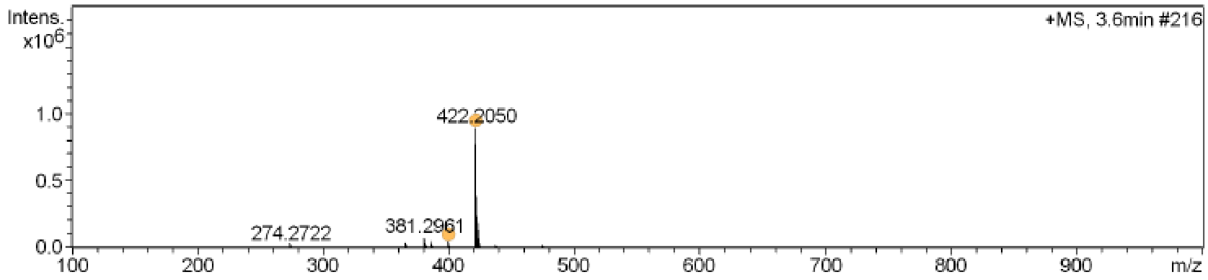

Meas. m/z	#	Ion Formula	m/z	err [ppm]	mSigma	# Sigma	Score	rdb	e¯ Conf	N-Rule
400.2223	1	C22H30N3O4	400.2231	-2.0	41.0	1	100.00	9.5	even	ok
422.2050	1	C22H29N3NaO4	422.2050	-0.0	11.7	1	100.00	9.5	even	ok

Figure S20. ***tert*-Butyl 4-[4-(methoxycarbonyl)-1-(3-methylphenyl)-1*H*-pyrazol-5-yl]piperidine-1-carboxylate (5c). HRMS (ESI-TOF).**

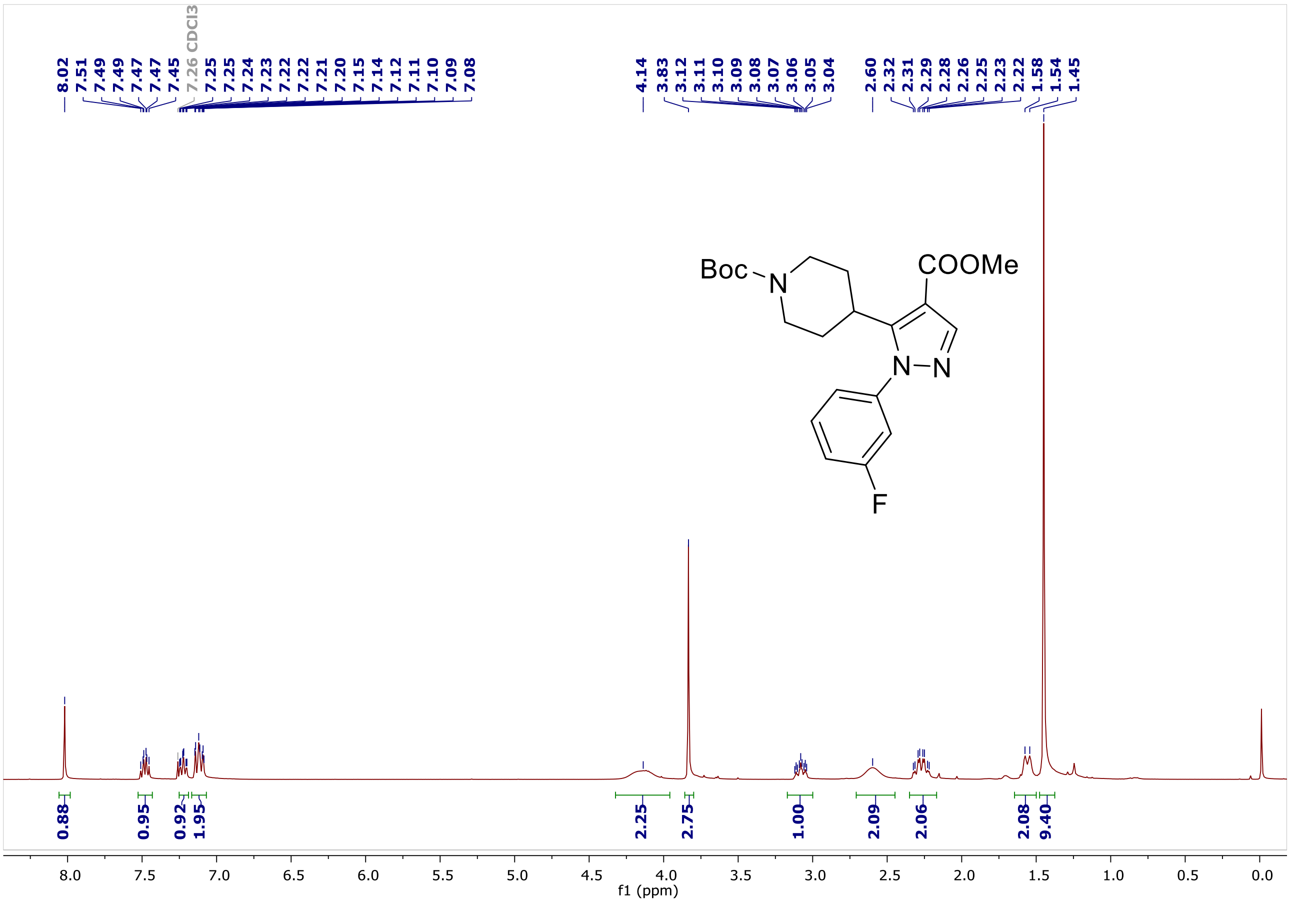

Figure S21. ***tert*-Butyl 4-[1-(3-fluorophenyl)-4-(methoxycarbonyl)-1*H*-pyrazol-5-yl]piperidine-1-carboxylate (5d). ^{1}H NMR spectrum (400 MHz, CDCl$_3$).**

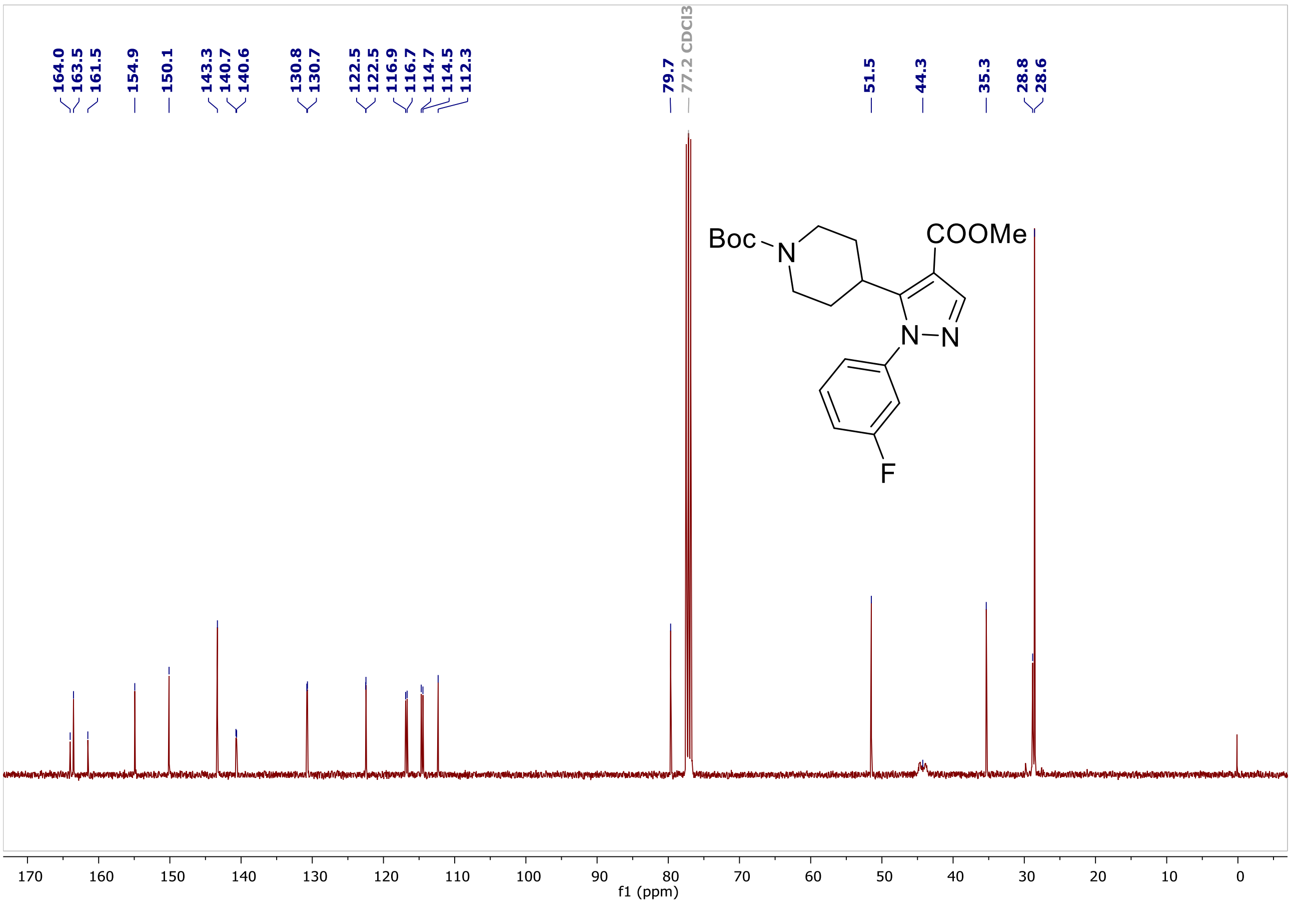

Figure S22. ***tert*-Butyl 4-[1-(3-fluorophenyl)-4-(methoxycarbonyl)-1*H*-pyrazol-5-yl]piperidine-1-carboxylate (5d). ^{13}C NMR spectrum (101 MHz, CDCl$_3$).**

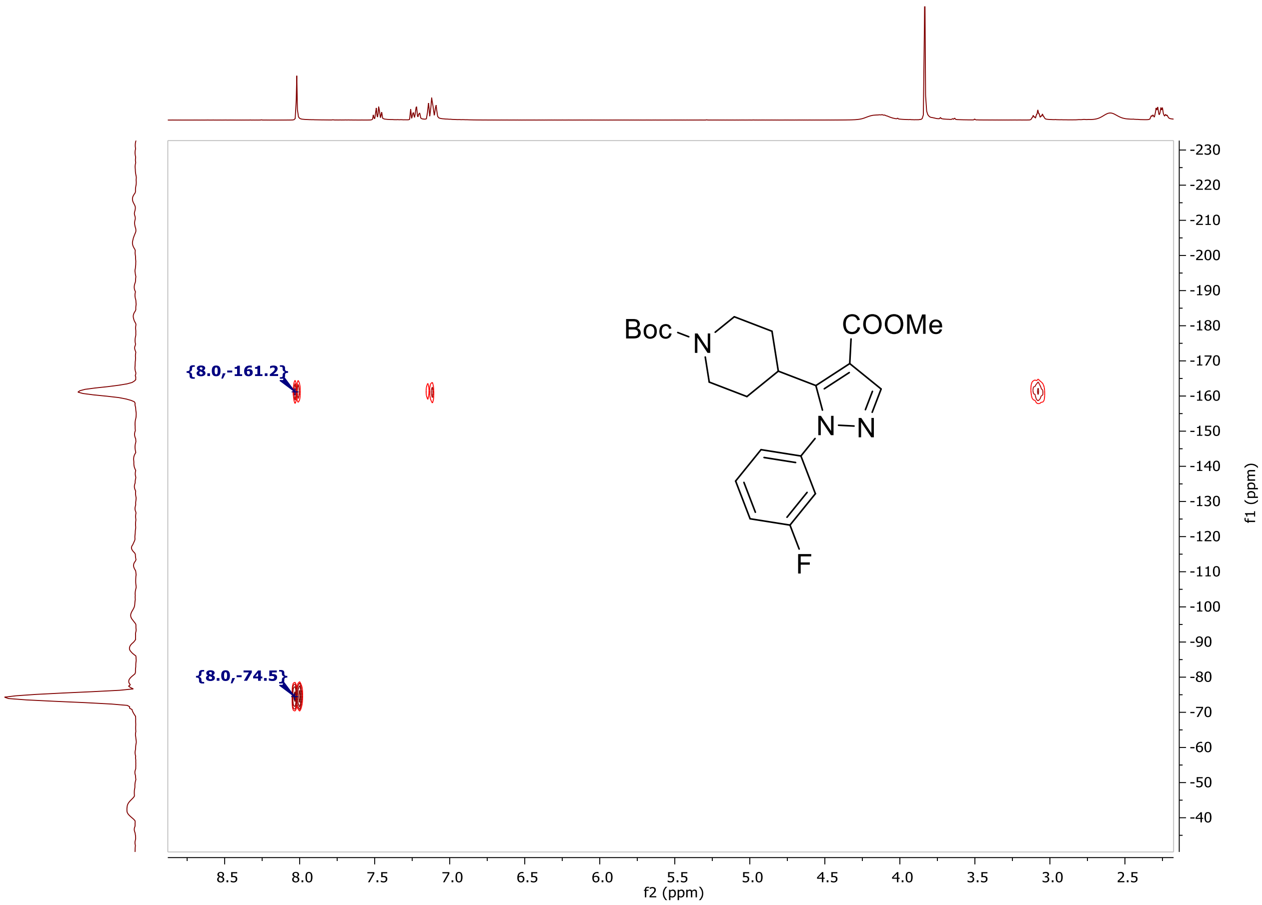

Figure S23. ***tert*-Butyl 4-[1-(3-fluorophenyl)-4-(methoxycarbonyl)-1*H*-pyrazol-5-yl]piperidine-1-carboxylate (5d). ^{1}H-^{15}N HMBC spectrum (41 MHz, $CDCl_3$).**

Compound Spectrum SmartFormula Report

Analysis Info

Analysis Name	D:\Data\GMP-014.d	Acquisition Date	11/27/2018 2:20:31 PM
Method	DirectInfusion_TuneLow_pos.m	Operator	hplc
Sample Name	GMP-014	Instrument	micrOTOF-Q III 8228888.20448
Comment	SB		

Acquisition Parameter

Source Type	ESI	Ion Polarity	Positive	Set Nebulizer	0.4 Bar
Focus	Not active	Set Capillary	4500 V	Set Dry Heater	180 °C
Scan Begin	50 m/z	Set End Plate Offset	-500 V	Set Dry Gas	4.0 l/min
Scan End	1000 m/z	Set Collision Cell RF	140.0 Vpp	Set Divert Valve	Waste

#	RT [min]	Area	Int. Type	I	S/N	Chromatogram	Max. m/z	FWHM [min]
n.a.	0.3	n.a.	Single spectrum	n.a.	n.a.	n.a.	304.2632	n.a.
n.a.	3.3	n.a.	Single spectrum	n.a.	n.a.	n.a.	426.1799	n.a.

+MS, 3.3min #200

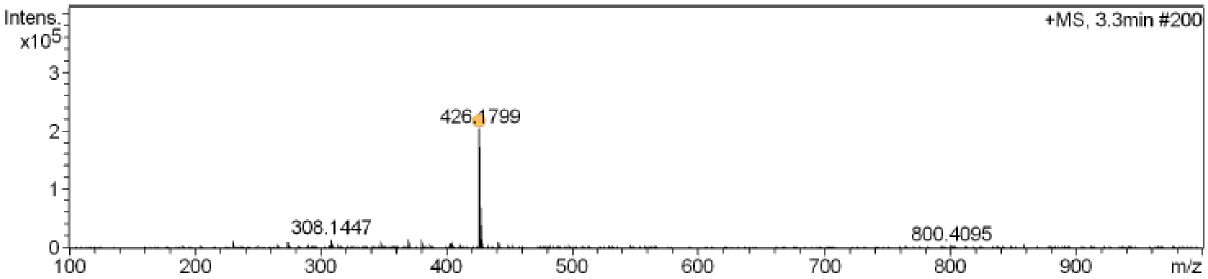

Meas. m/z	#	Ion Formula	m/z	err [ppm]	mSigma	# Sigma	Score	rdb	e⁻ Conf	N-Rule
426.1799	1	C21H26FN3NaO4	426.1800	0.2	8.8	1	100.00	9.5	even	ok

Figure S24. ***tert*-Butyl 4-[1-(3-fluorophenyl)-4-(methoxycarbonyl)-1*H*-pyrazol-5-yl]piperidine-1-carboxylate (5d). HRMS (ESI-TOF).**

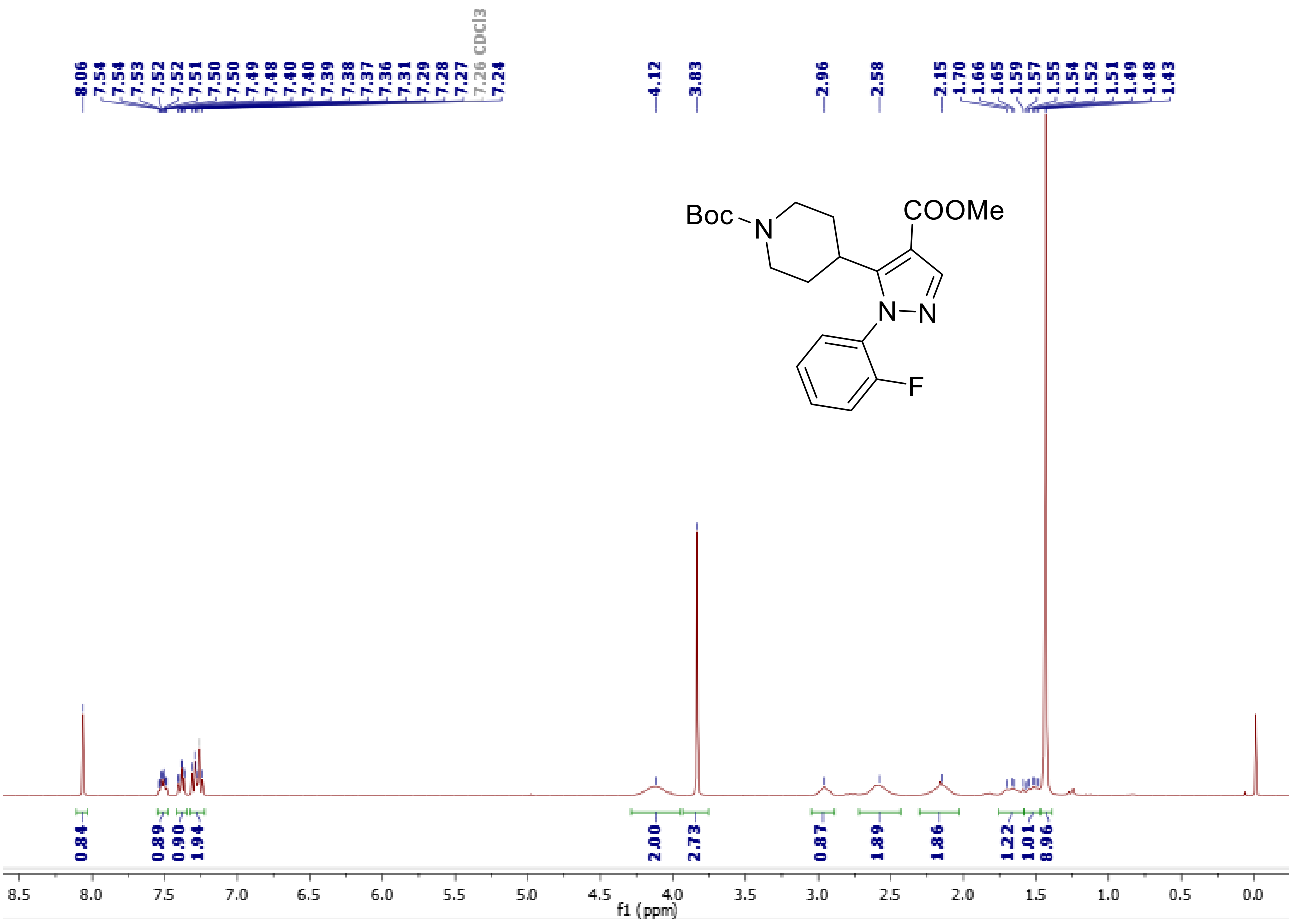

Figure S25. ***tert*-Butyl 4-[1-(2-fluorophenyl)-4-(methoxycarbonyl)-1*H*-pyrazol-5-yl]piperidine-1-carboxylate (5e). ¹H NMR spectrum (400 MHz, $CDCl_3$).**

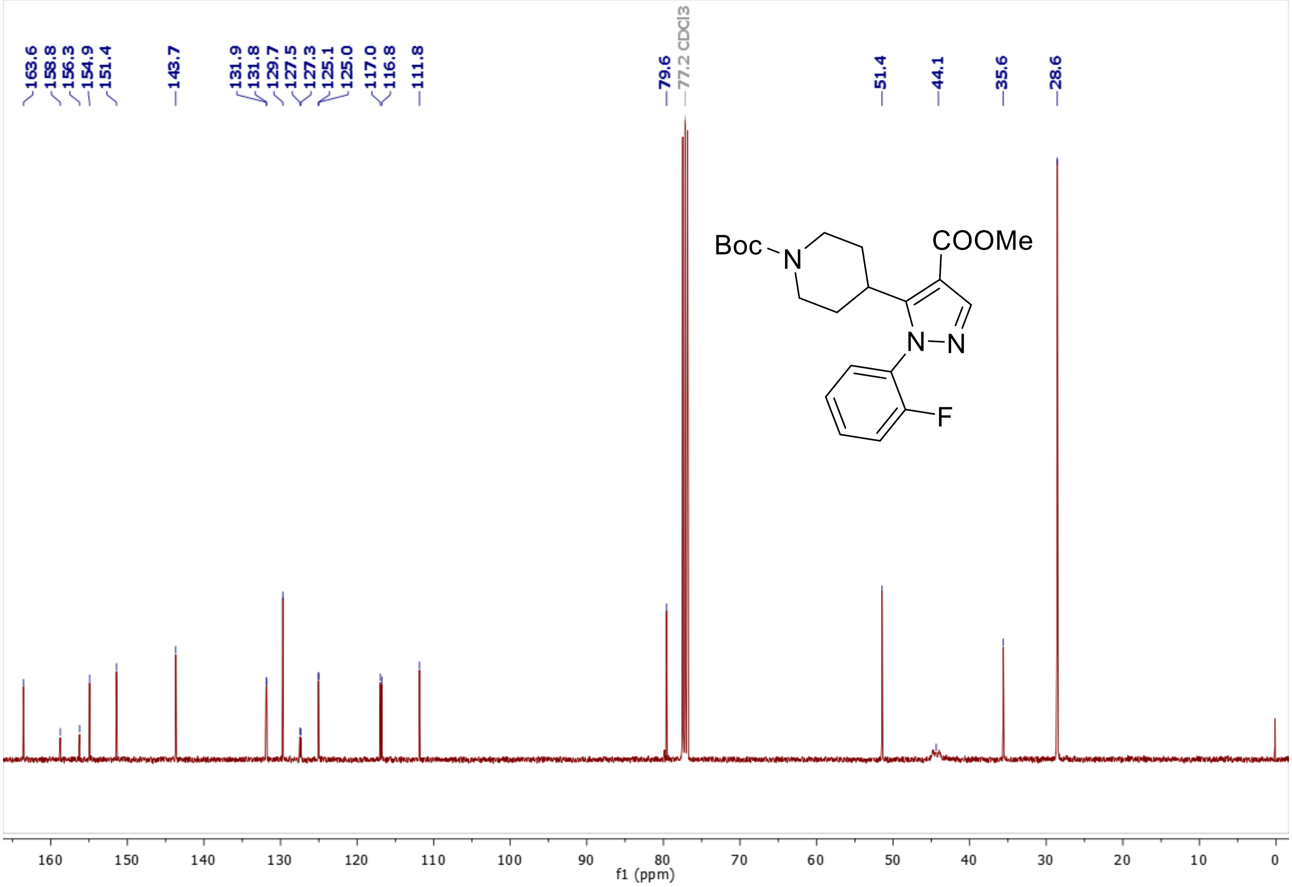

Figure S26. ***tert*-Butyl 4-[1-(2-fluorophenyl)-4-(methoxycarbonyl)-1*H*-pyrazol-5-yl]piperidine-1-carboxylate (5e). ¹³C NMR spectrum (101 MHz, $CDCl_3$).**

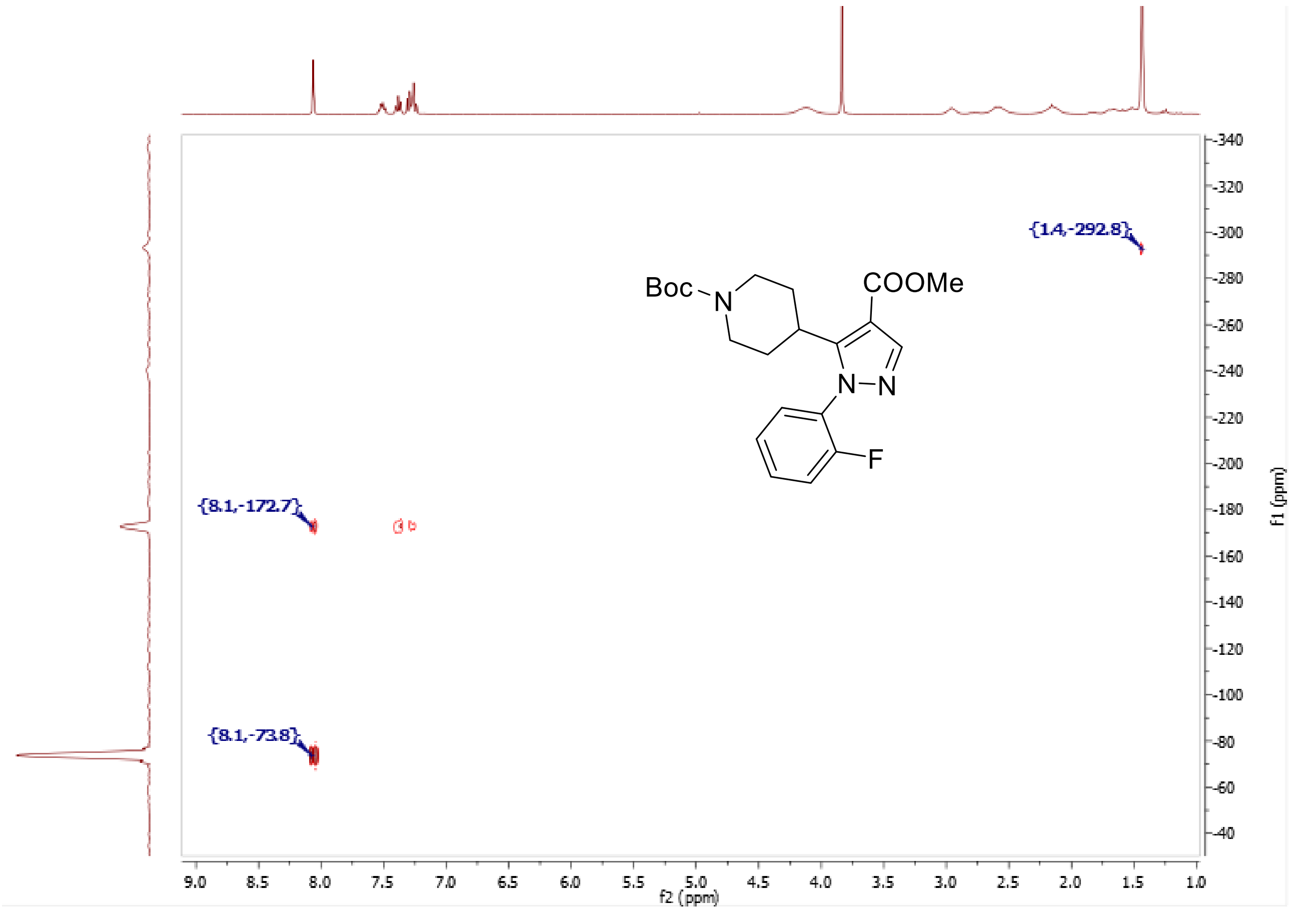

Figure S27. ***tert*-Butyl 4-[1-(2-fluorophenyl)-4-(methoxycarbonyl)-1*H*-pyrazol-5-yl]piperidine-1-carboxylate (5e). ^{1}H-^{15}N HMBC spectrum (41 MHz, $CDCl_3$).**

Compound Spectrum SmartFormula Report

Analysis Info

Analysis Name D:\Data\GMP-013.d
Method DirectInfusion_TuneLow_pos.m
Sample Name GMP-013
Comment SB

Acquisition Date 11/27/2018 1:33:39 PM
Operator hplc
Instrument micrOTOF-Q III 8228888.20448

Acquisition Parameter

Source Type	ESI	Ion Polarity	Positive	Set Nebulizer	0.4 Bar
Focus	Not active	Set Capillary	4500 V	Set Dry Heater	180 °C
Scan Begin	50 m/z	Set End Plate Offset	-500 V	Set Dry Gas	4.0 l/min
Scan End	1000 m/z	Set Collision Cell RF	140.0 Vpp	Set Divert Valve	Waste

#	RT [min]	Area	Int. Type	I	S/N	Chromatogram	Max. m/z	FWHM [min]
n.a.	0.2	n.a.	Single spectrum	n.a.	n.a.	n.a.	304.2632	n.a.
n.a.	2.4	n.a.	Single spectrum	n.a.	n.a.	n.a.	426.1800	n.a.

+MS, 2.4min #145

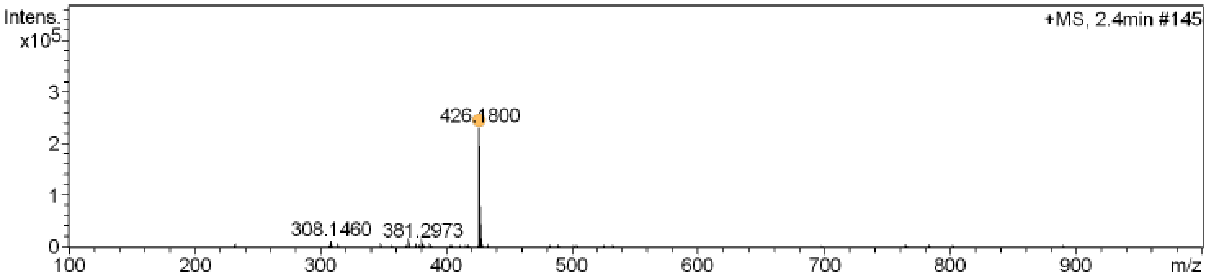

Meas. m/z	#	Ion Formula	m/z	err [ppm]	mSigma	# Sigma	Score	rdb	e⁻ Conf	N-Rule
426.1800	1	C21H26FN3NaO4	426.1800	0.1	4.3	1	100.00	9.5	even	ok

Figure S28. *tert*-Butyl 4-[1-(2-fluorophenyl)-4-(methoxycarbonyl)-1*H*-pyrazol-5-yl]piperidine-1-carboxylate (5e). HRMS (ESI-TOF).

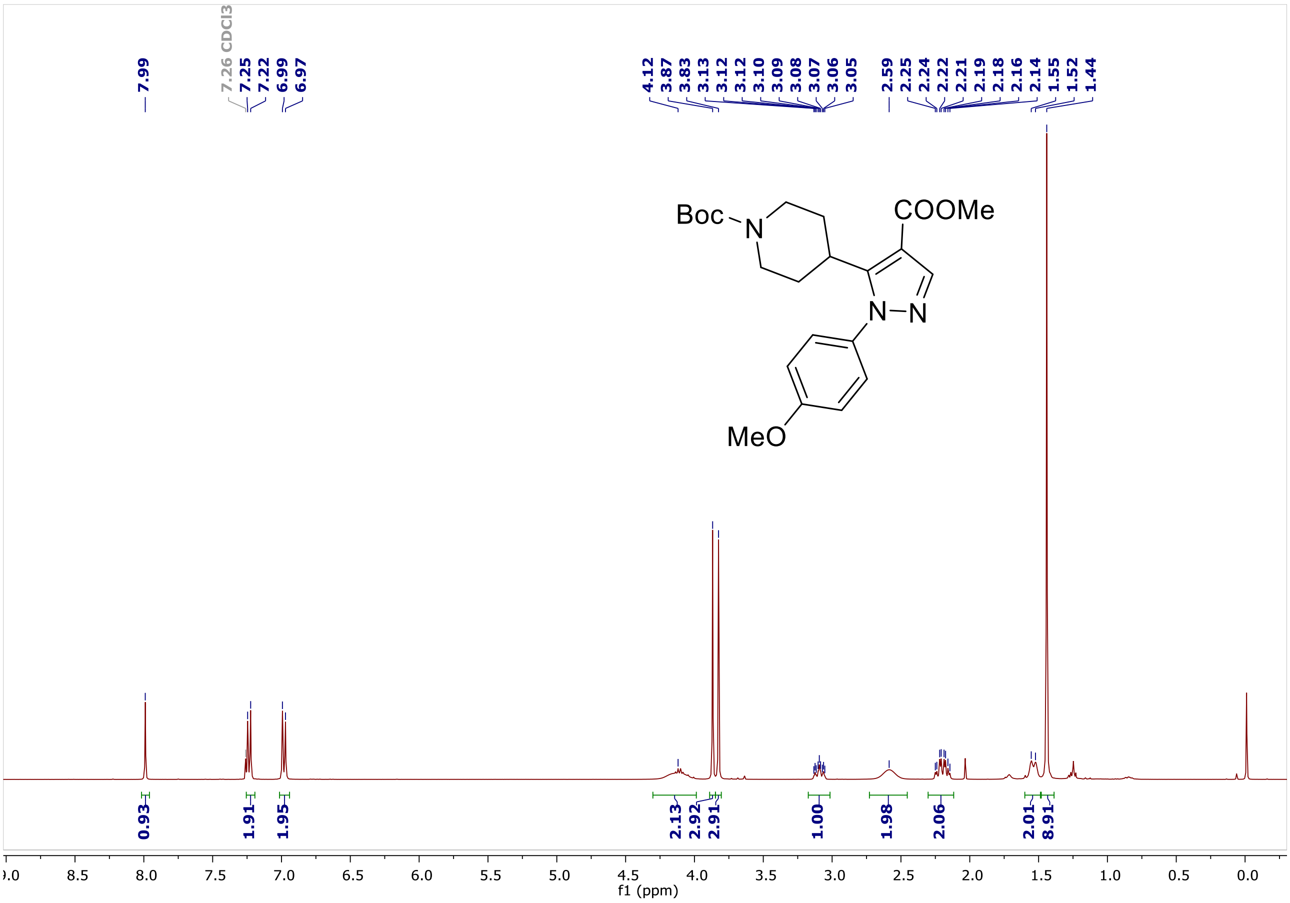

Figure S29. ***tert*-Butyl 4-[4-(methoxycarbonyl)-1-(4-methoxyphenyl)-1*H*-pyrazol-5-yl]piperidine-1-carboxylate (5f). ^{1}H NMR spectrum (400 MHz, $CDCl_3$).**

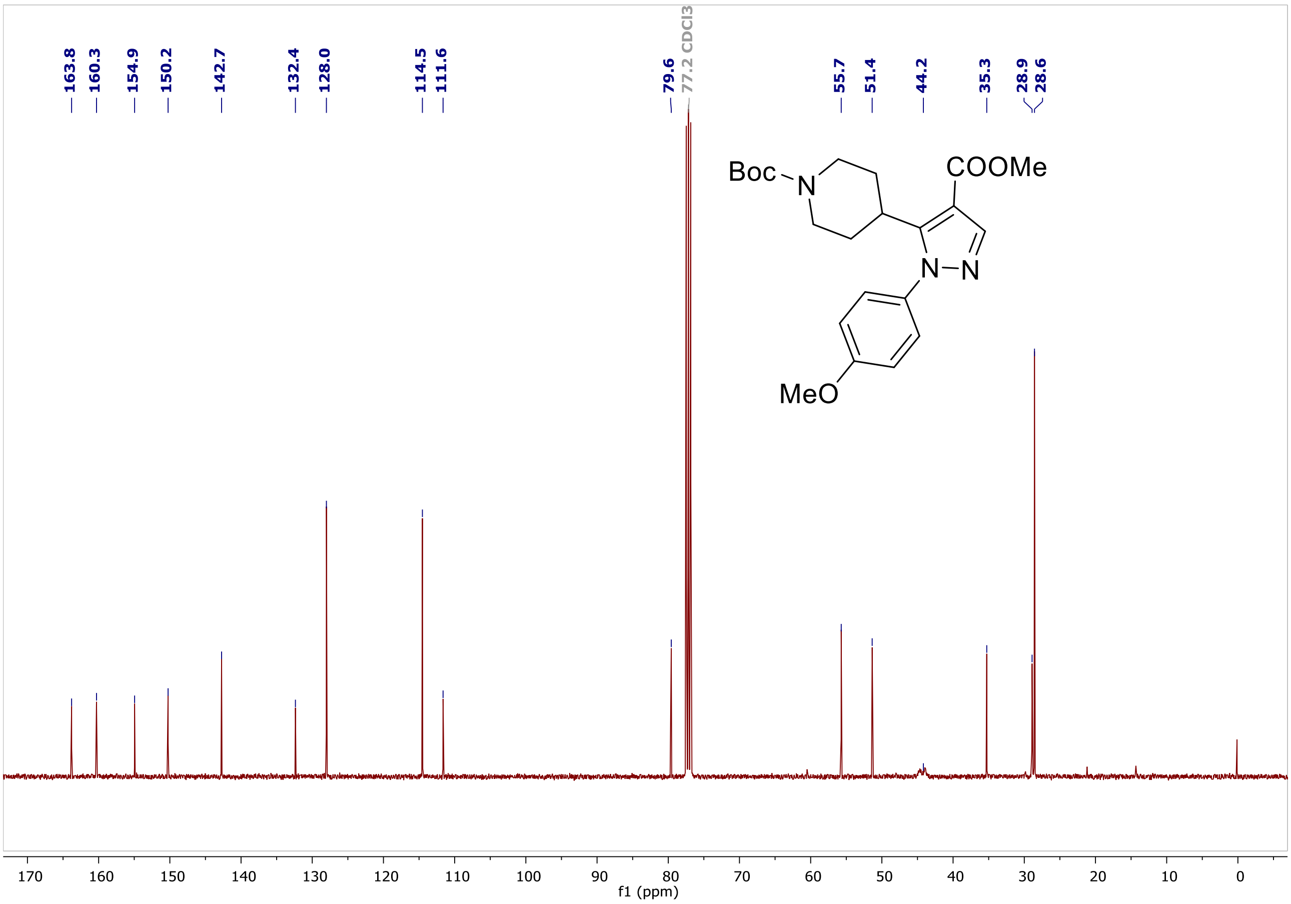

Figure S30. ***tert*-Butyl 4-[4-(methoxycarbonyl)-1-(4-methoxyphenyl)-1*H*-pyrazol-5-yl]piperidine-1-carboxylate (5f). ^{13}C NMR spectrum (176 MHz, $CDCl_3$).**

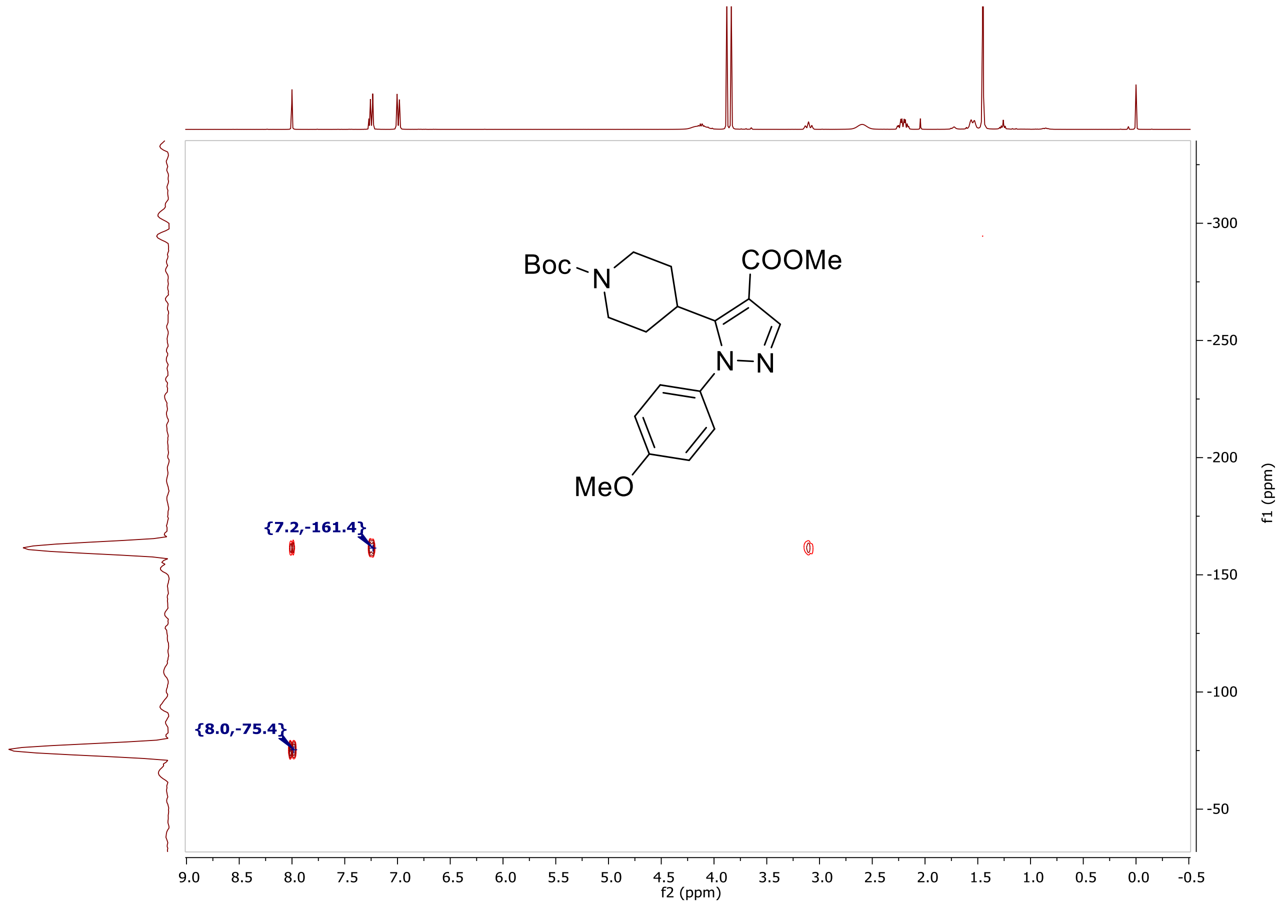

Figure S31. ***tert*-Butyl 4-[4-(methoxycarbonyl)-1-(4-methoxyphenyl)-1*H*-pyrazol-5-yl]piperidine-1-carboxylate (5f). ^{15}N NMR spectrum (41 MHz, $CDCl_3$).**

Compound Spectrum SmartFormula Report

Analysis Info

Analysis Name	D:\Data\GMP-017.d	Acquisition Date	11/27/2018 2:08:37 PM
Method	DirectInfusion_TuneLow_pos.m	Operator	hplc
Sample Name	GMP-017	Instrument	micrOTOF-Q III 8228888.20448
Comment	SB		

Acquisition Parameter

Source Type	ESI	Ion Polarity	Positive	Set Nebulizer	0.4 Bar
Focus	Not active	Set Capillary	4500 V	Set Dry Heater	180 °C
Scan Begin	50 m/z	Set End Plate Offset	-500 V	Set Dry Gas	4.0 l/min
Scan End	1000 m/z	Set Collision Cell RF	140.0 Vpp	Set Divert Valve	Waste

#	RT [min]	Area	Int. Type	I	S/N	Chromatogram	Max. m/z	FWHM [min]
n.a.	0.2	n.a.	Single spectrum	n.a.	n.a.	n.a.	304.2631	n.a.
n.a.	2.2	n.a.	Single spectrum	n.a.	n.a.	n.a.	438.2000	n.a.

+MS, 2.2min #130

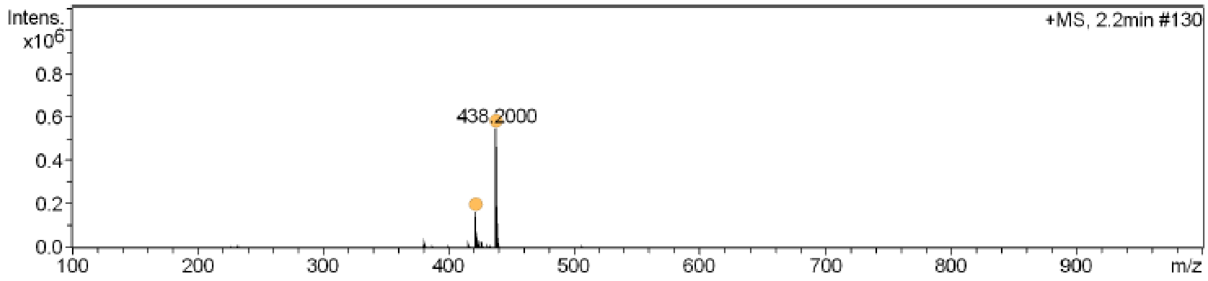

Meas. m/z	#	Ion Formula	m/z	err [ppm]	mSigma	# Sigma	Score	rdb	e⁻ Conf	N-Rule
422.2046	1	C22H29N3NaO4	422.2050	1.0	4.5	1	100.00	9.5	even	ok
438.2000	1	C22H29N3NaO5	438.1999	0.0	4.4	1	100.00	9.5	even	ok

Figure S32. ***tert*-Butyl 4-[4-(methoxycarbonyl)-1-(4-methoxyphenyl)-1*H*-pyrazol-5-yl]piperidine-1-carboxylate (5f). HRMS (ESI-TOF).**

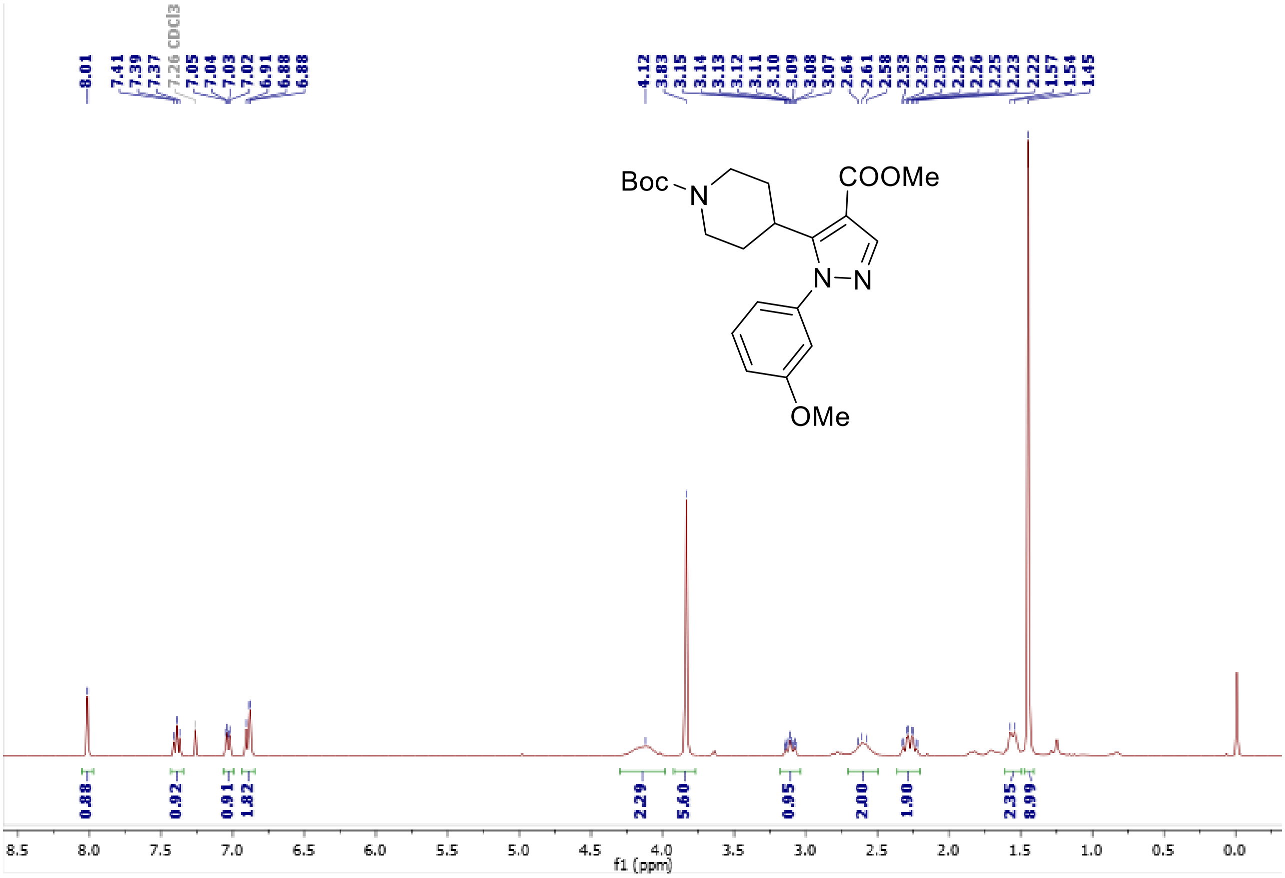

Figure S33. ***tert*-Butyl 4-[4-(methoxycarbonyl)-1-(3-methoxyphenyl)-1*H*-pyrazol-5-yl]piperidine-1-carboxylate (5g). ^{1}H NMR spectrum (400 MHz, CDCl$_3$).**

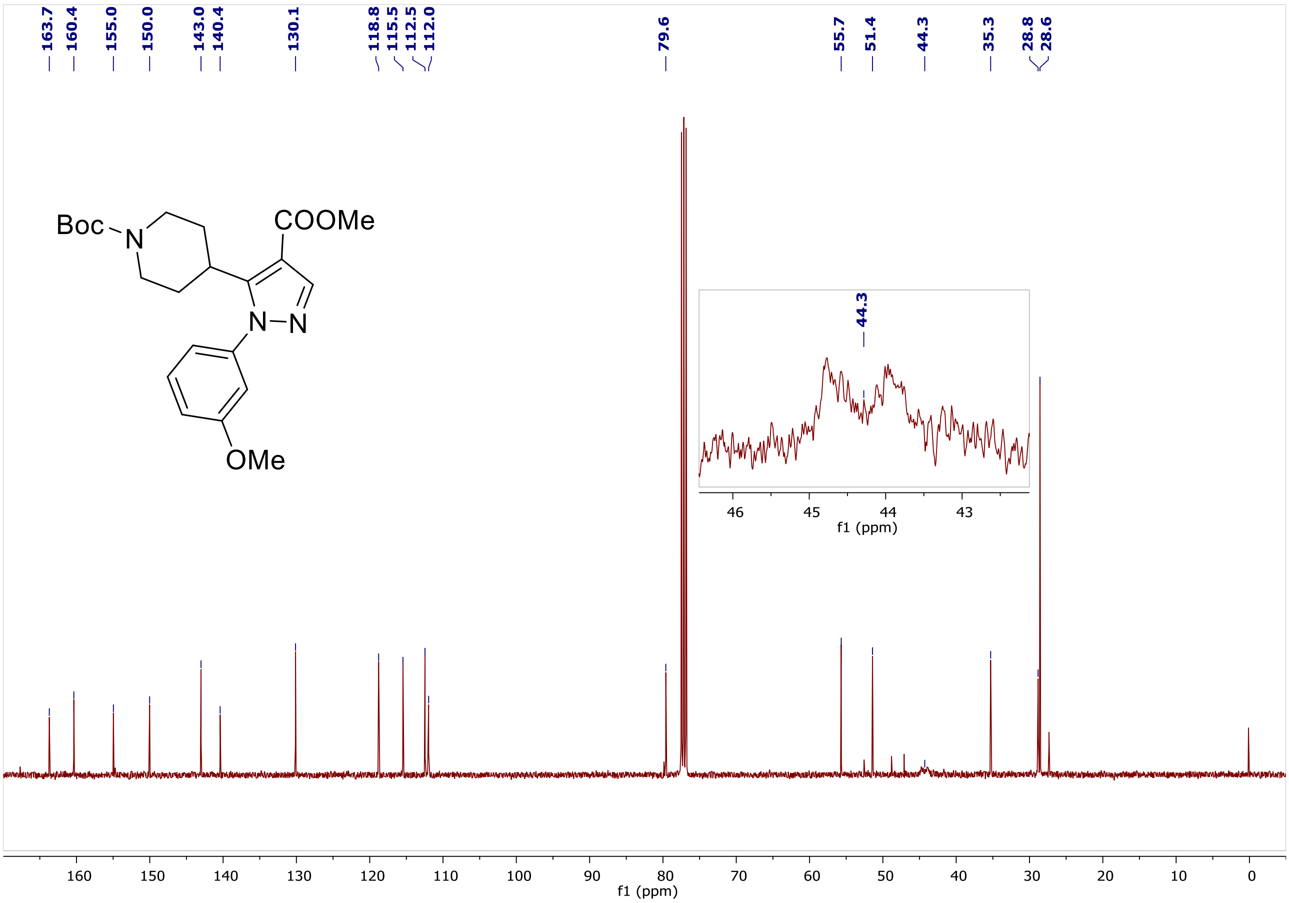

Figure S34. ***tert*-Butyl 4-[4-(methoxycarbonyl)-1-(3-methoxyphenyl)-1*H*-pyrazol-5-yl]piperidine-1-carboxylate (5g). ^{13}C NMR spectrum (101 MHz, CDCl$_3$).**

Compound Spectrum SmartFormula Report

Analysis Info

Analysis Name	D:\Data\GMP-16.d	Acquisition Date	2/4/2021 11:39:59 PM
Method	DirectInfusion_TuneLow_pos.m	Operator	hplc
Sample Name	GMP-16	Instrument	micrOTOF-Q III 8228888.20448
Comment	AB		

Acquisition Parameter

Source Type	ESI	Ion Polarity	Positive	Set Nebulizer	0.4 Bar
Focus	Not active	Set Capillary	4500 V	Set Dry Heater	180 °C
Scan Begin	50 m/z	Set End Plate Offset	-500 V	Set Dry Gas	4.0 l/min
Scan End	1000 m/z	Set Collision Cell RF	140.0 Vpp	Set Divert Valve	Waste

#	RT [min]	Area	Int. Type	I	S/N	Chromatogram	Max. m/z	FWHM [min]
n.a.	2.8	n.a.	Single spectrum	n.a.	n.a.	n.a.	438.2000	n.a.

+MS, 2.8min #165

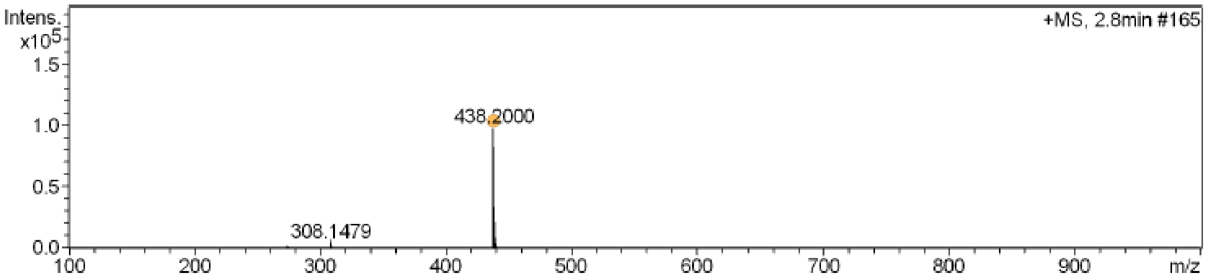

Meas. m/z	#	Ion Formula	m/z	err [ppm]	mSigma	# Sigma	Score	rdb	e⁻ Conf	N-Rule
438.2000	1	C22H29N3NaO5	438.1999	-0.0	2.3	1	100.00	9.5	even	ok

Figure S35. ***tert*****-Butyl 4-[4-(methoxycarbonyl)-1-(3-methoxyphenyl)-1*H*-pyrazol-5-yl]piperidine-1-carboxylate (5g). HRMS (ESI-TOF).**

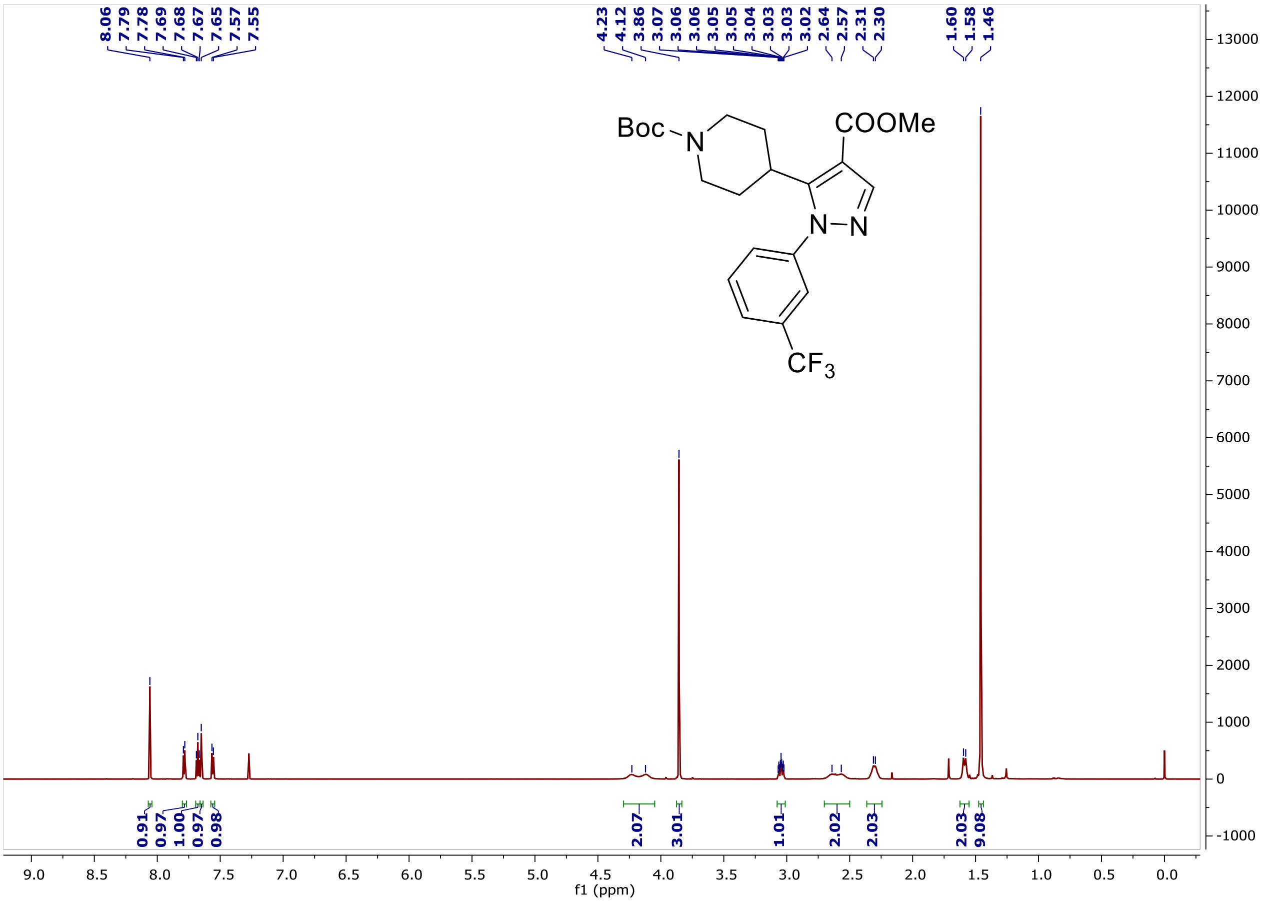

Figure S36. ***tert*-Butyl 4-{4-(methoxycarbonyl)-1-[3-(trifluoromethyl)phenyl]-1*H*-pyrazol-5-yl}piperidine-1-carboxylate (5h). ^{1}H NMR spectrum (700 MHz, $CDCl_3$).**

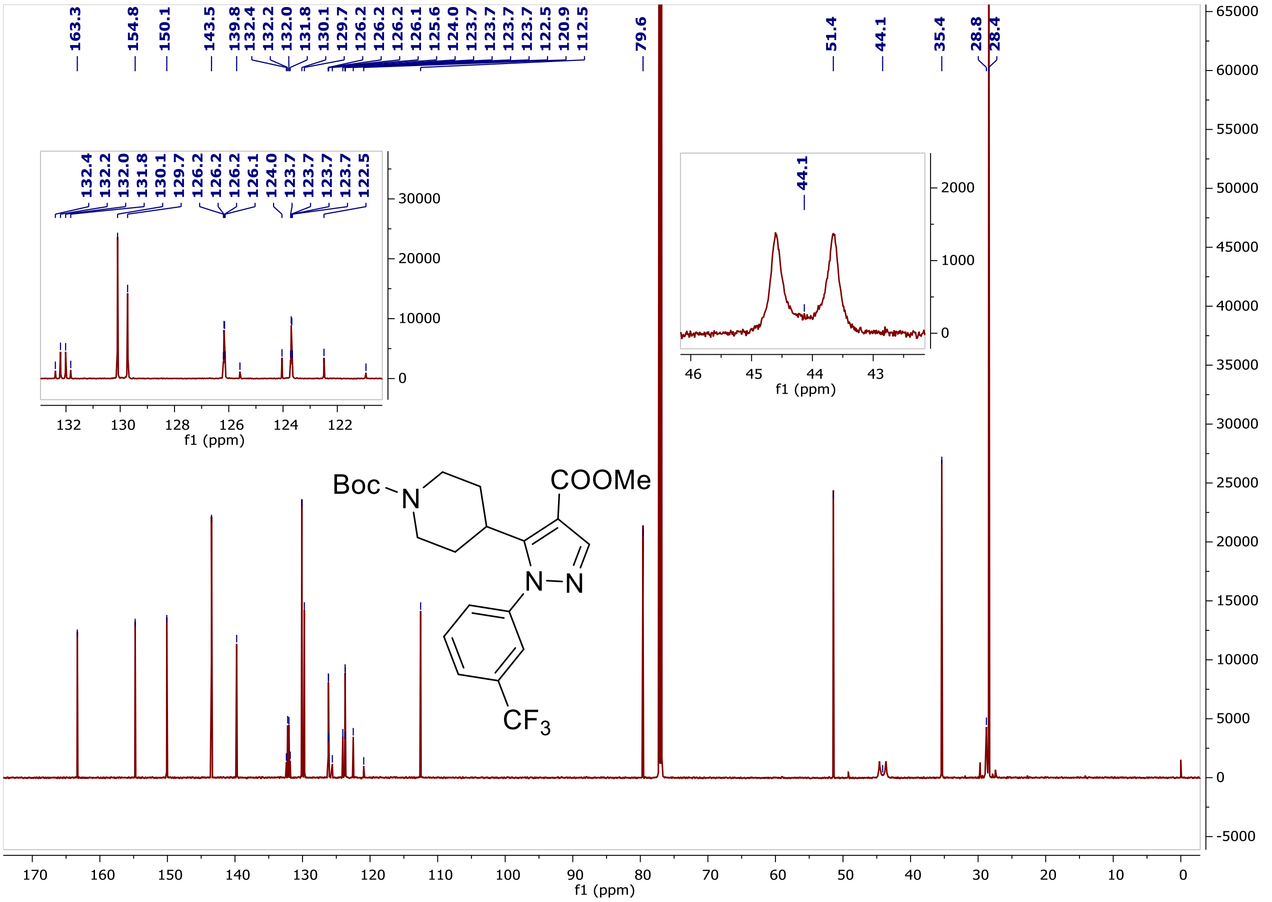

Figure S37. ***tert*-Butyl 4-{4-(methoxycarbonyl)-1-[3-(trifluoromethyl)phenyl]-1*H*-pyrazol-5-yl}piperidine-1-carboxylate (5h). ^{13}C NMR spectrum (176 MHz, $CDCl_3$).**

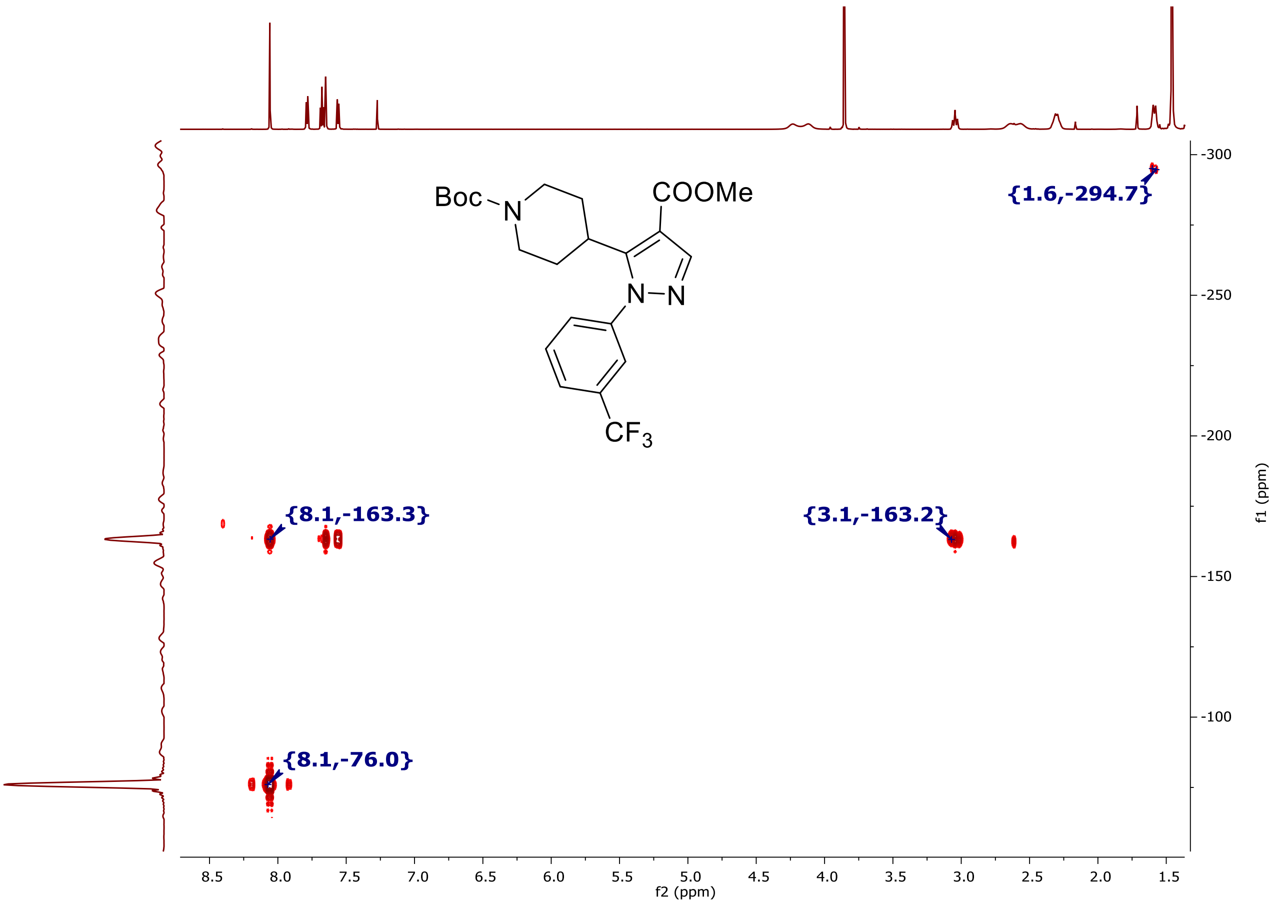

Figure S38. ***tert*****-Butyl 4-{4-(methoxycarbonyl)-1-[3-(trifluoromethyl)phenyl]-1*H*-pyrazol-5-yl}piperidine-1-carboxylate (5h). ^{1}H-^{15}N HMBC spectrum (71 MHz, $CDCl_3$).**

Compound Spectrum SmartFormula Report

Analysis Info

Analysis Name	D:\Data\GMP-035.d	Acquisition Date	11/29/2018 11:19:05 AM
Method	DirectInfusion_TuneLow_pos.m	Operator	hplc
Sample Name	GMP-035	Instrument	micrOTOF-Q III 8228888.20448
Comment	SB		

Acquisition Parameter

Source Type	ESI	Ion Polarity	Positive	Set Nebulizer	0.4 Bar
Focus	Not active	Set Capillary	4500 V	Set Dry Heater	180 °C
Scan Begin	50 m/z	Set End Plate Offset	-500 V	Set Dry Gas	4.0 l/min
Scan End	1000 m/z	Set Collision Cell RF	140.0 Vpp	Set Divert Valve	Waste

#	RT [min]	Area	Int. Type	I	S/N	Chromatogram	Max. m/z	FWHM [min]
n.a.	0.5	n.a.	Single spectrum	n.a.	n.a.	n.a.	226.9518	n.a.
n.a.	3.5	n.a.	Single spectrum	n.a.	n.a.	n.a.	476.1768	n.a.

+MS, 3.5min #212

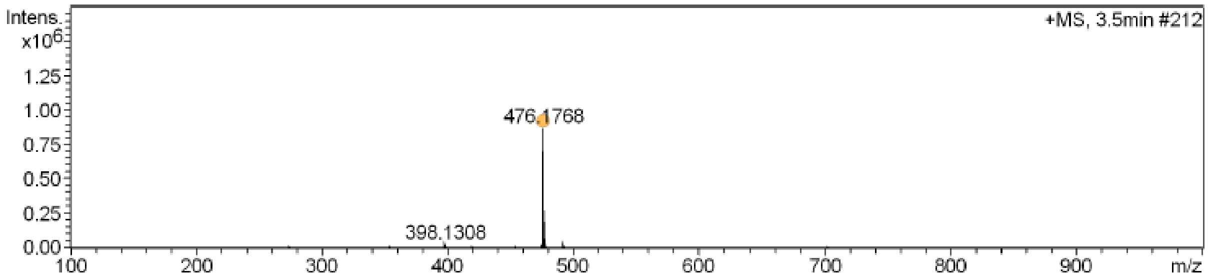

Meas. m/z	#	Ion Formula	m/z	err [ppm]	mSigma	# Sigma	Score	rdb	e¯ Conf	N-Rule
476.1768	1	C22H26F3N3NaO4	476.1768	-0.1	7.6	1	100.00	9.5	even	ok

Figure S39. ***tert*-Butyl 4-{4-(methoxycarbonyl)-1-[3-(trifluoromethyl)phenyl]-1*H*-pyrazol-5-yl}piperidine-1-carboxylate (5h). HRMS (ESI-TOF).**

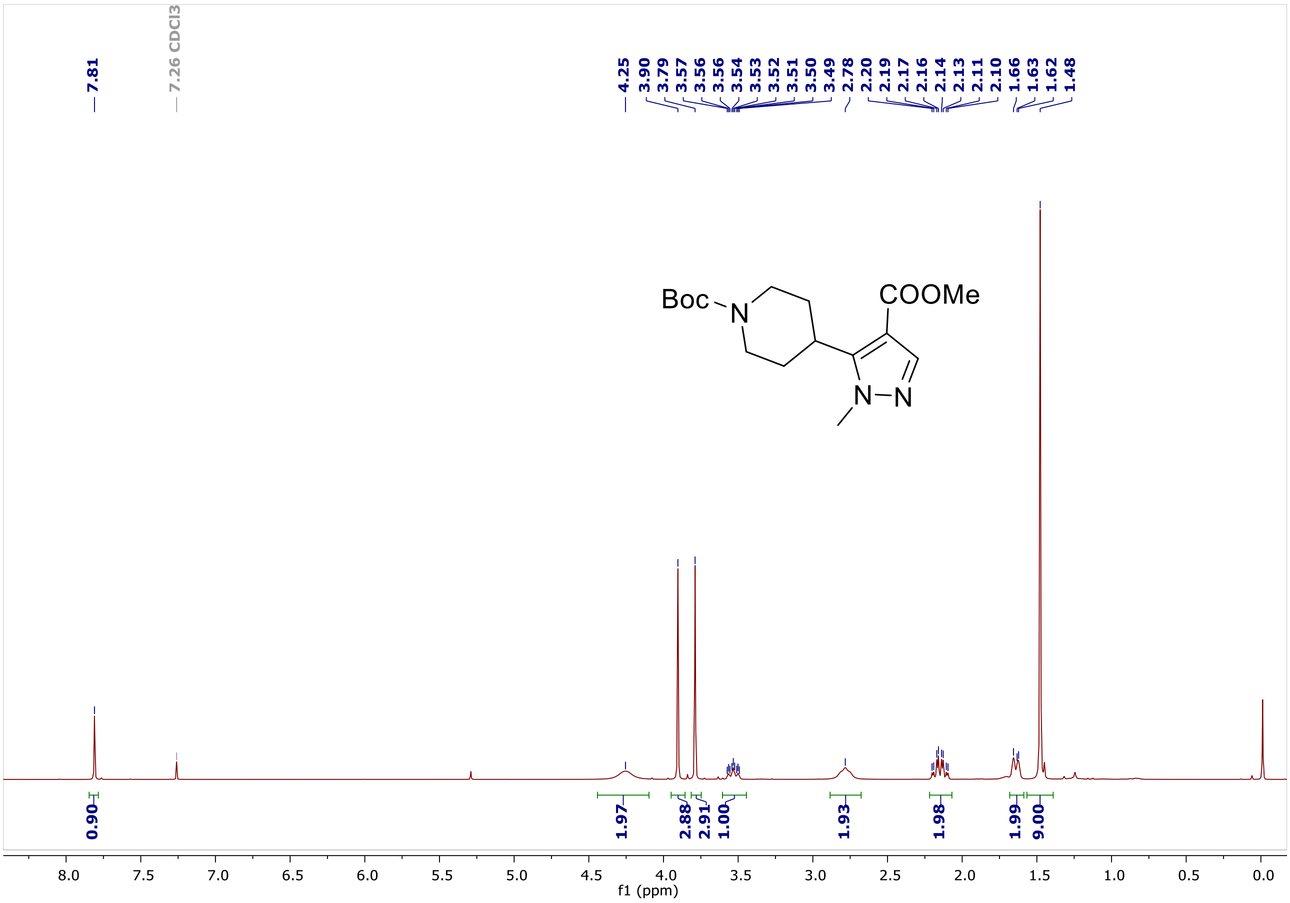

Figure S40. ***tert*-Butyl 4-[4-(methoxycarbonyl)-1-methyl-1*H*-pyrazol-5-yl]piperidine-1-carboxylate (5i). ^{1}H NMR spectrum (400 MHz, $CDCl_3$).**

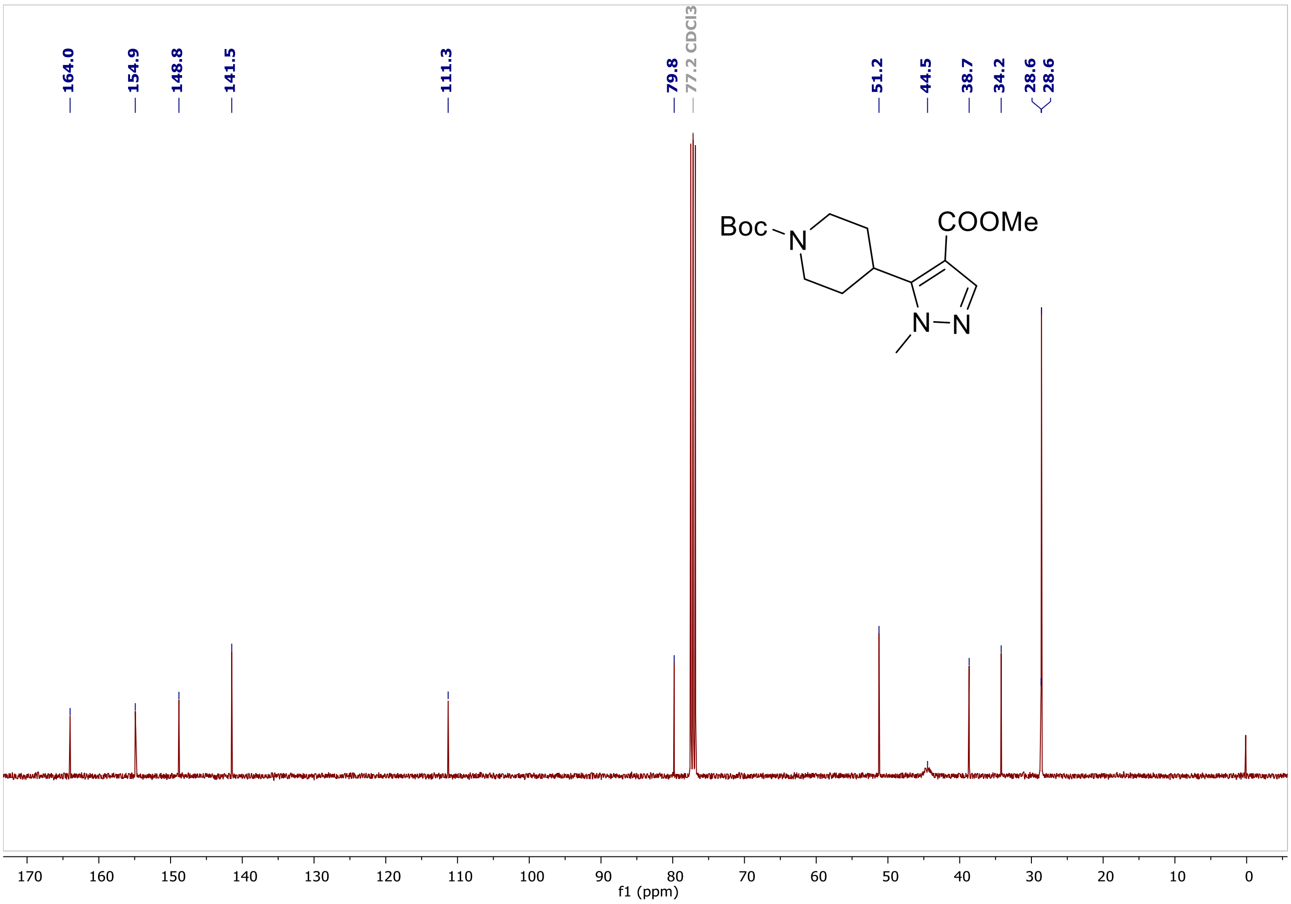

Figure S41. ***tert*-Butyl 4-[4-(methoxycarbonyl)-1-methyl-1*H*-pyrazol-5-yl]piperidine-1-carboxylate (5i). ^{13}C NMR spectrum (101 MHz, $CDCl_3$).**

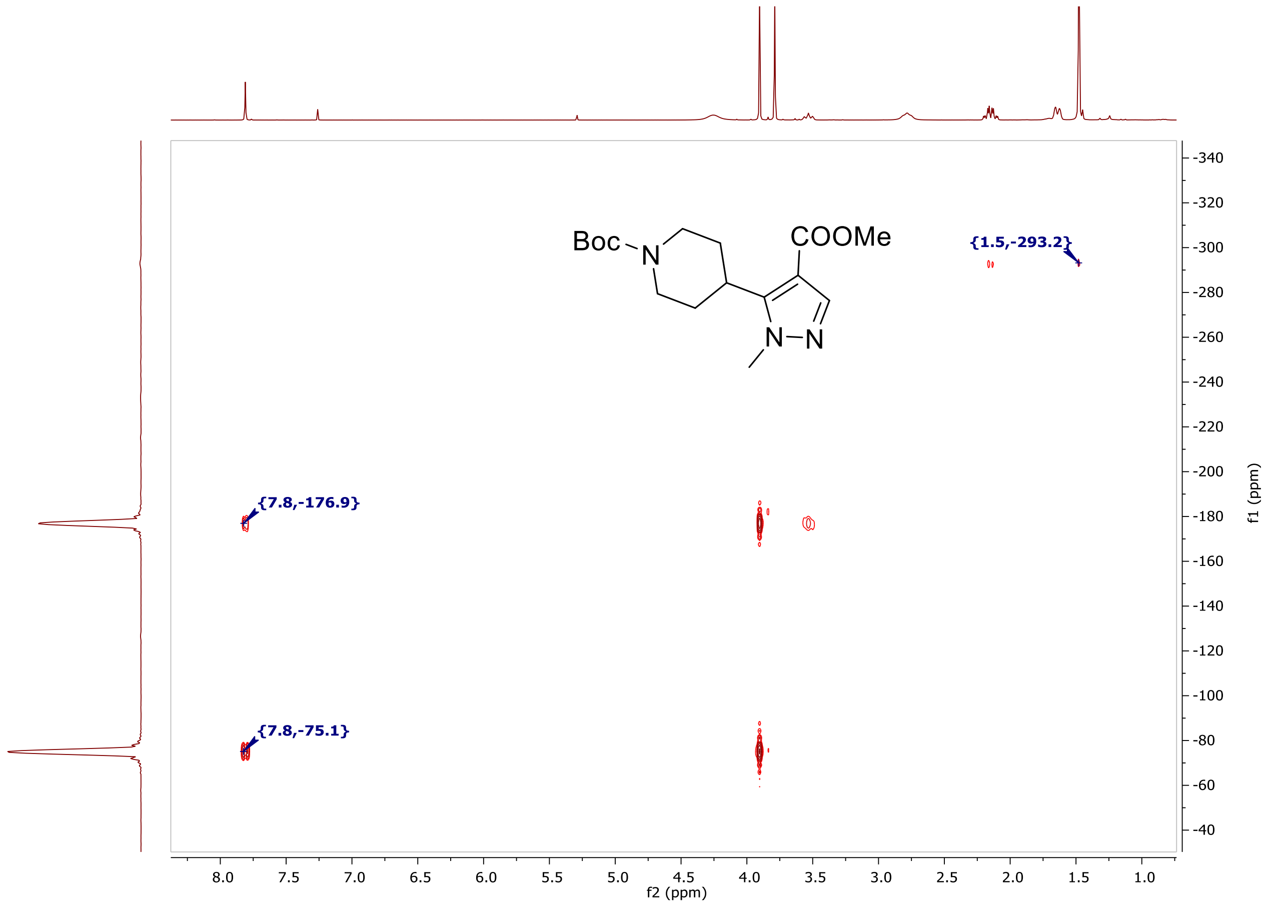

Figure S42. ***tert*-Butyl 4-[4-(methoxycarbonyl)-1-methyl-1*H*-pyrazol-5-yl]piperidine-1-carboxylate (5i). ^{1}H-^{15}N HMBC spectrum (41 MHz, $CDCl_3$).**

Compound Spectrum SmartFormula Report

Analysis Info

Analysis Name	D:\Data\GMP-012.d	Acquisition Date	11/27/2018 11:31:36 AM
Method	DirectInfusion_TuneLow_pos.m	Operator	hplc
Sample Name	GMP-012	Instrument	micrOTOF-Q III 8228888.20448
Comment	SB		

Acquisition Parameter

Source Type	ESI	Ion Polarity	Positive	Set Nebulizer	0.4 Bar
Focus	Not active	Set Capillary	4500 V	Set Dry Heater	180 °C
Scan Begin	50 m/z	Set End Plate Offset	-500 V	Set Dry Gas	4.0 l/min
Scan End	1000 m/z	Set Collision Cell RF	140.0 Vpp	Set Divert Valve	Waste

#	RT [min]	Area	Int. Type	I	S/N	Chromatogram	Max. m/z	FWHM [min]
n.a.	0.2	n.a.	Single spectrum	n.a.	n.a.	n.a.	304.2632	n.a.
n.a.	4.5	n.a.	Single spectrum	n.a.	n.a.	n.a.	346.1737	n.a.

+MS, 4.5min #271

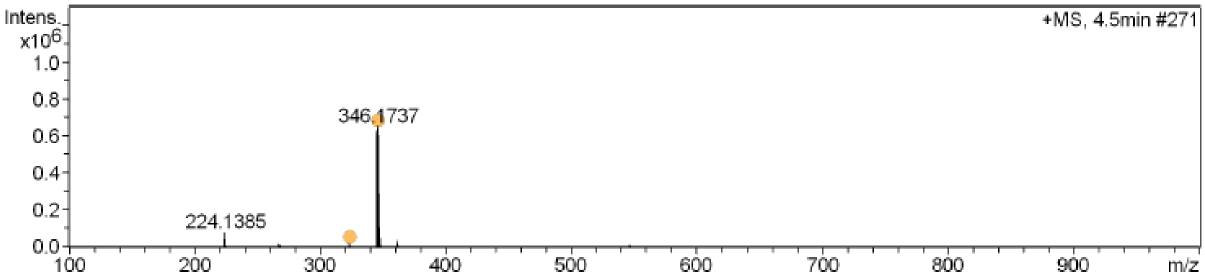

Meas. m/z	#	Ion Formula	m/z	err [ppm]	mSigma	# Sigma	Score	rdb	e⁻ Conf	N-Rule
324.1906	1	C16H26N3O4	324.1918	-3.5	21.5	1	100.00	5.5	even	ok
346.1737	1	C16H25N3NaO4	346.1737	0.1	2.4	1	100.00	5.5	even	ok

Figure S43. *tert*-Butyl 4-[4-(methoxycarbonyl)-1-methyl-1*H*-pyrazol-5-yl]piperidine-1-carboxylate (5i). HRMS (ESI-TOF).

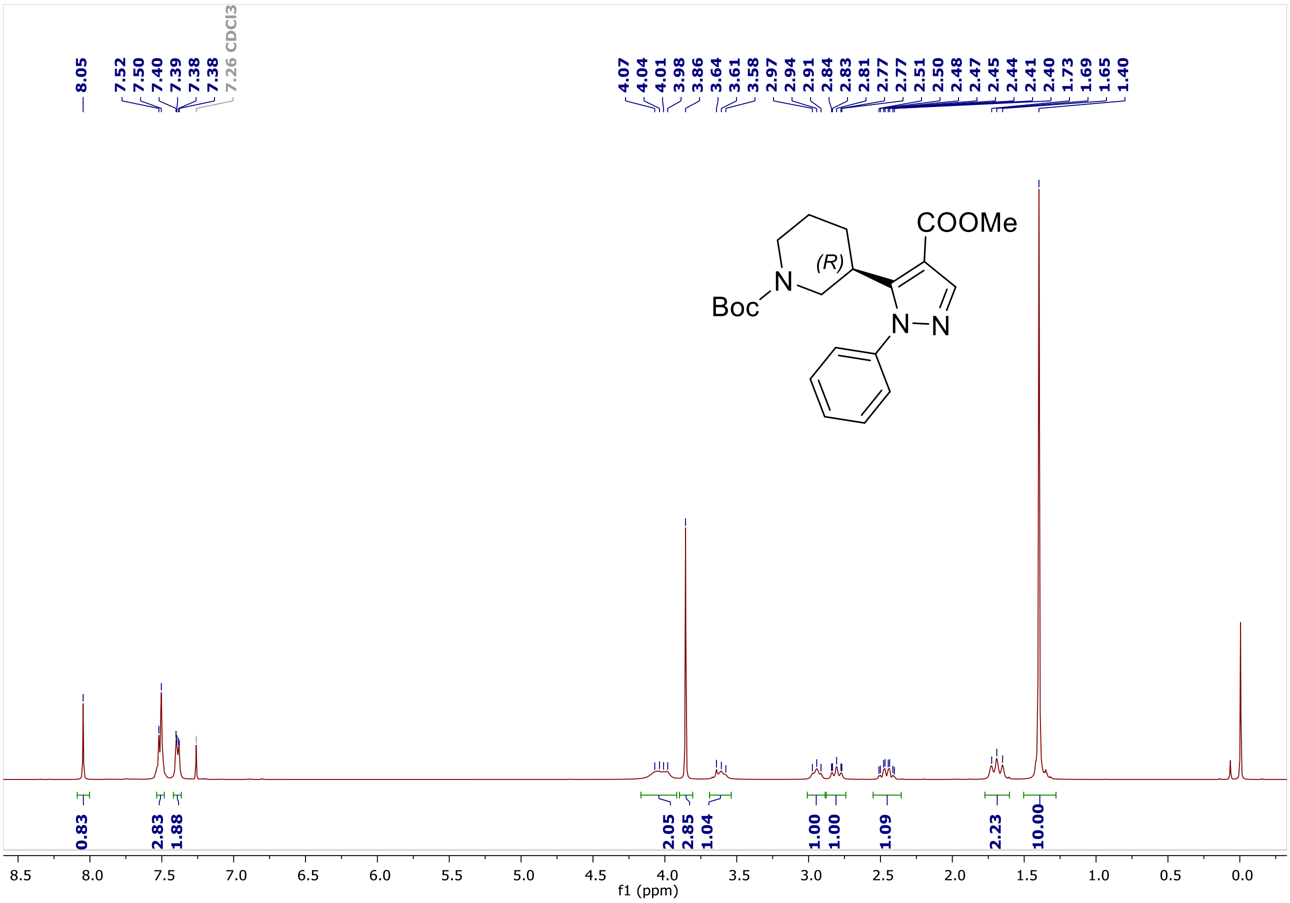

Figure S44. ***tert*-Butyl (3*R*)-3-[4-(methoxycarbonyl)-1-phenyl-1*H*-pyrazol-5-yl]piperidine-1-carboxylate (5j). ^{1}H NMR spectrum (400 MHz, $CDCl_3$).**

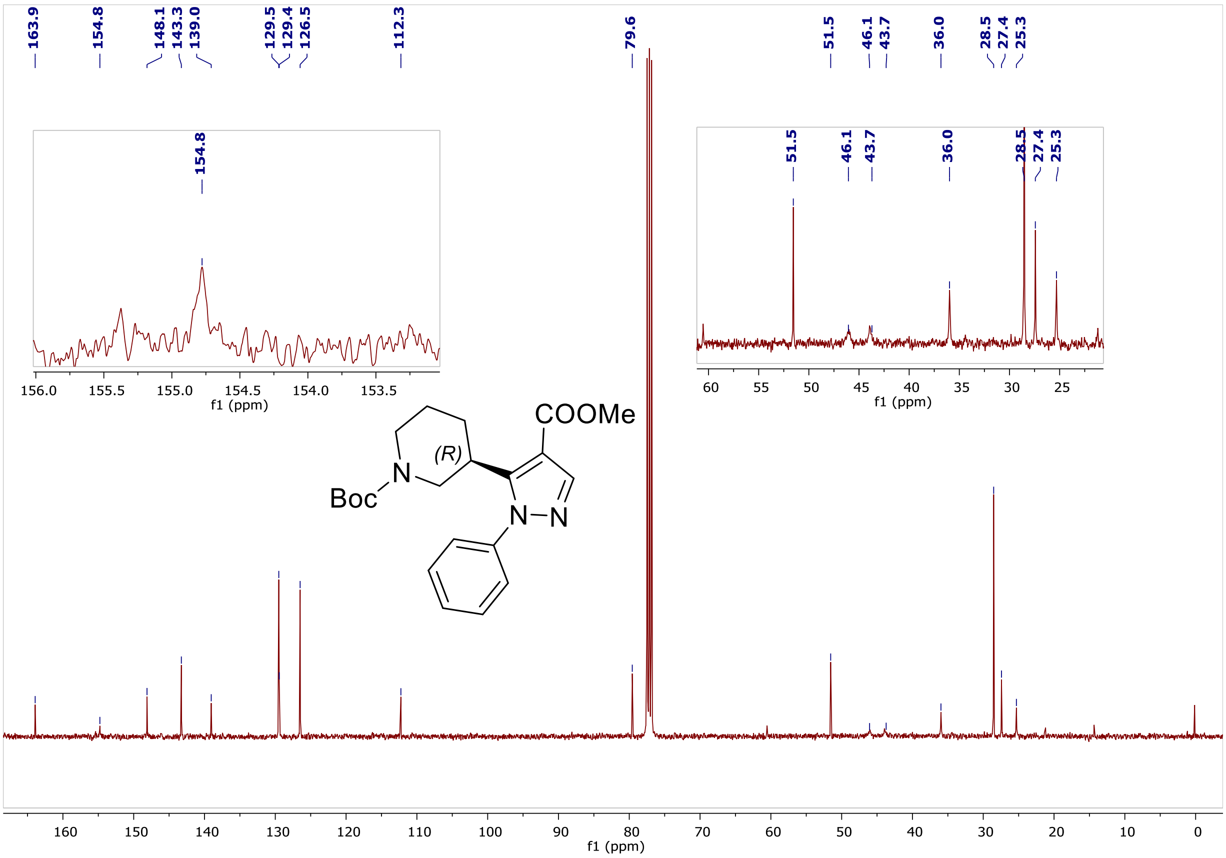

Figure S45. ***tert*-Butyl (3*R*)-3-[4-(methoxycarbonyl)-1-phenyl-1*H*-pyrazol-5-yl]piperidine-1-carboxylate (5j). ^{13}C NMR spectrum (101 MHz, $CDCl_3$).**

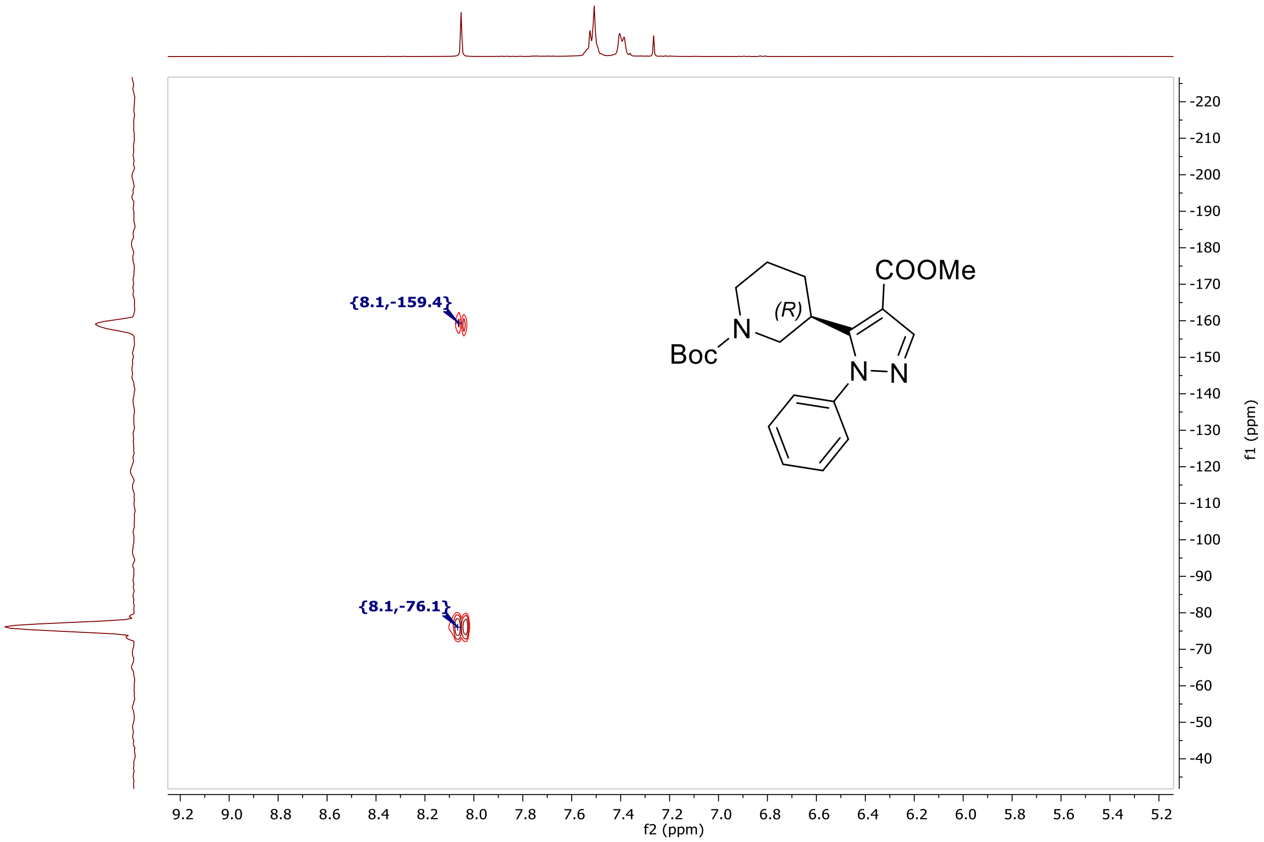

Figure S46. ***tert*-Butyl (3*R*)-3-[4-(methoxycarbonyl)-1-phenyl-1*H*-pyrazol-5-yl]piperidine-1-carboxylate (5j). ^{1}H-^{15}N HMBC spectrum (41 MHz, $CDCl_3$).**

Compound Spectrum SmartFormula Report

Analysis Info

Analysis Name	D:\Data\GMP-117.d	Acquisition Date	2/5/2021 12:32:27 AM
Method	DirectInfusion_TuneLow_pos.m	Operator	hplc
Sample Name	GMP-117	Instrument	micrOTOF-Q III 8228888.20448
Comment	AB		

Acquisition Parameter

Source Type	ESI	Ion Polarity	Positive	Set Nebulizer	0.4 Bar
Focus	Not active	Set Capillary	4500 V	Set Dry Heater	180 °C
Scan Begin	50 m/z	Set End Plate Offset	-500 V	Set Dry Gas	4.0 l/min
Scan End	1000 m/z	Set Collision Cell RF	140.0 Vpp	Set Divert Valve	Waste

#	RT [min]	Area	Int. Type	I	S/N	Chromatogram	Max. m/z	FWHM [min]
n.a.	4.3	n.a.	Single spectrum	n.a.	n.a.	n.a.	408.1893	n.a.

+MS, 4.3min #255

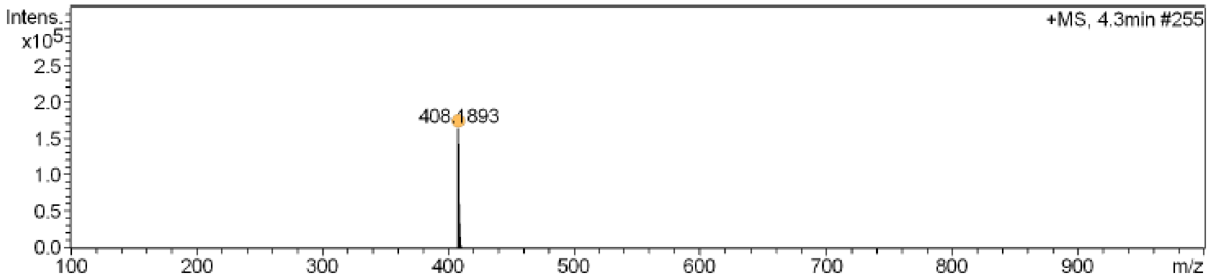

Meas. m/z	#	Ion Formula	m/z	err [ppm]	mSigma	# Sigma	Score	rdb	e⁻ Conf	N-Rule
408.1893	1	C21H27N3NaO4	408.1894	-0.1	2.9	1	100.00	9.5	even	ok

Figure S47. *tert*-Butyl (3*R*)-3-[4-(methoxycarbonyl)-1-phenyl-1*H*-pyrazol-5-yl]piperidine-1-carboxylate (5j). HRMS (ESI-TOF).

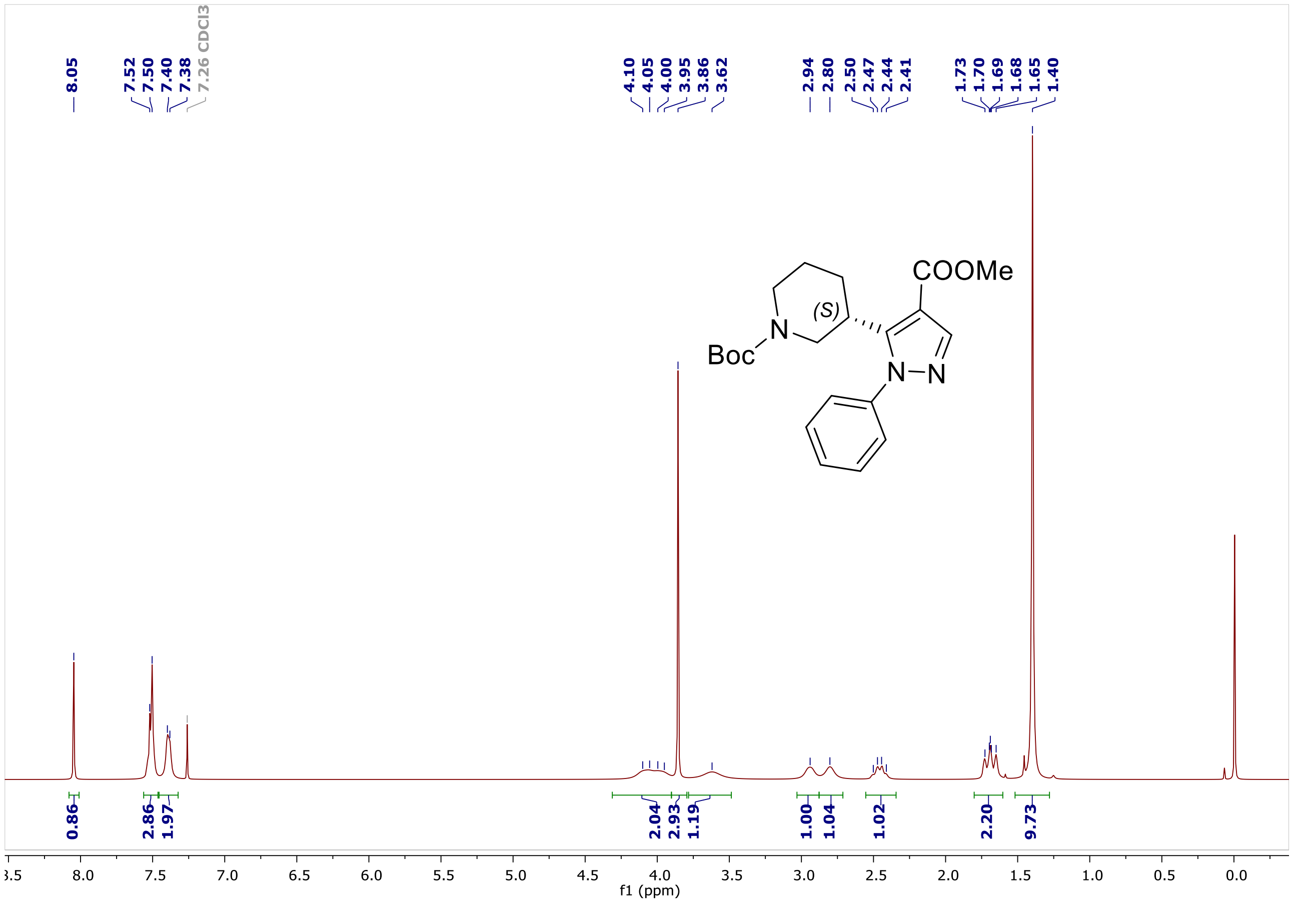

Figure S48. ***tert*-Butyl (3*S*)-3-[4-(methoxycarbonyl)-1-phenyl-1*H*-pyrazol-5-yl]piperidine-1-carboxylate (5k). ^{1}H NMR spectrum (400 MHz, $CDCl_3$).**

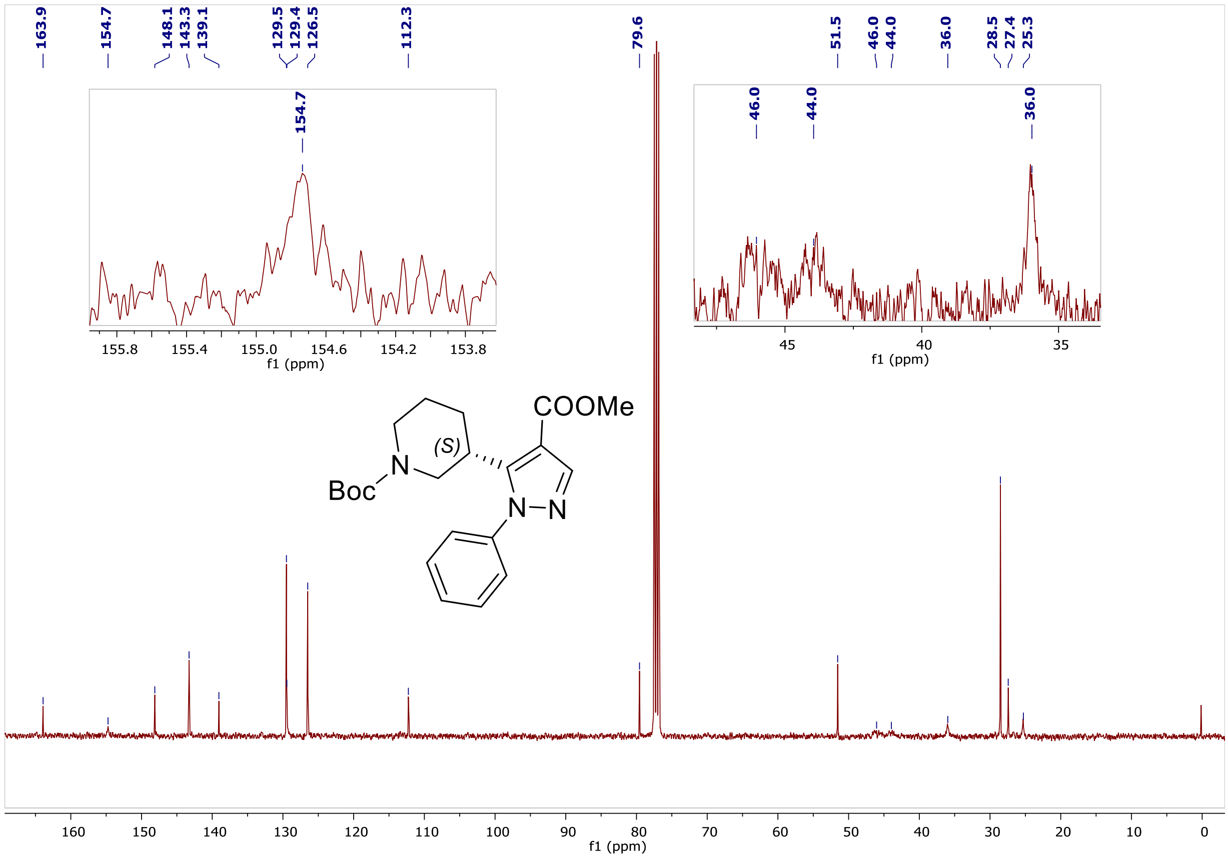

Figure S49. ***tert*-Butyl (3*S*)-3-[4-(methoxycarbonyl)-1-phenyl-1*H*-pyrazol-5-yl]piperidine-1-carboxylate (5k). ^{13}C NMR spectrum (101 MHz, $CDCl_3$).**

Compound Spectrum SmartFormula Report

Analysis Info

Analysis Name	D:\Data\GMP-146.d	Acquisition Date	2/5/2021 12:21:40 AM
Method	DirectInfusion_TuneLow_pos.m	Operator	hplc
Sample Name	GMP-146	Instrument	microTOF-Q III 8228888.20448
Comment	AB		

Acquisition Parameter

Source Type	ESI	Ion Polarity	Positive	Set Nebulizer	0.4 Bar
Focus	Not active	Set Capillary	4500 V	Set Dry Heater	180 °C
Scan Begin	50 m/z	Set End Plate Offset	-500 V	Set Dry Gas	4.0 l/min
Scan End	1000 m/z	Set Collision Cell RF	140.0 Vpp	Set Divert Valve	Waste

#	RT [min]	Area	Int. Type	I	S/N	Chromatogram	Max. m/z	FWHM [min]
n.a.	2.3	n.a.	Single spectrum	n.a.	n.a.	n.a.	408.1892	n.a.

+MS, 2.3min #140

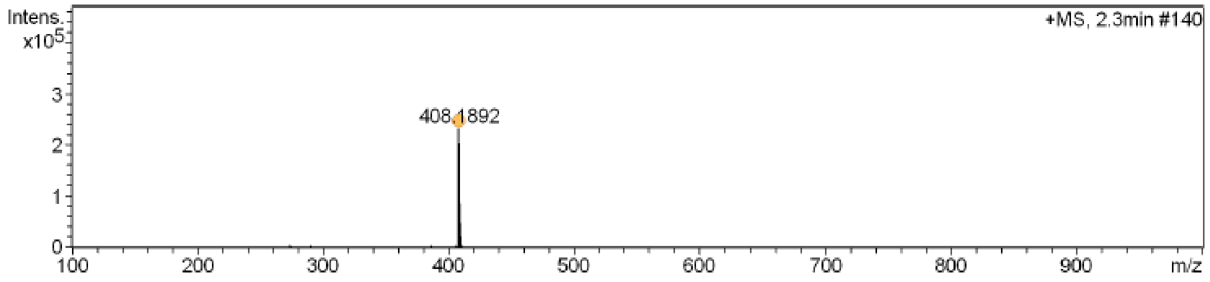

Meas. m/z	#	Ion Formula	m/z	err [ppm]	mSigma	# Sigma	Score	rdb	e⁻ Conf	N-Rule
408.1892	1	C21H27N3NaO4	408.1894	0.4	1.8	1	100.00	9.5	even	ok

Figure S50. *tert*-Butyl (3*S*)-3-[4-(methoxycarbonyl)-1-phenyl-1*H*-pyrazol-5-yl]piperidine-1-carboxylate (5k). HRMS (ESI-TOF).

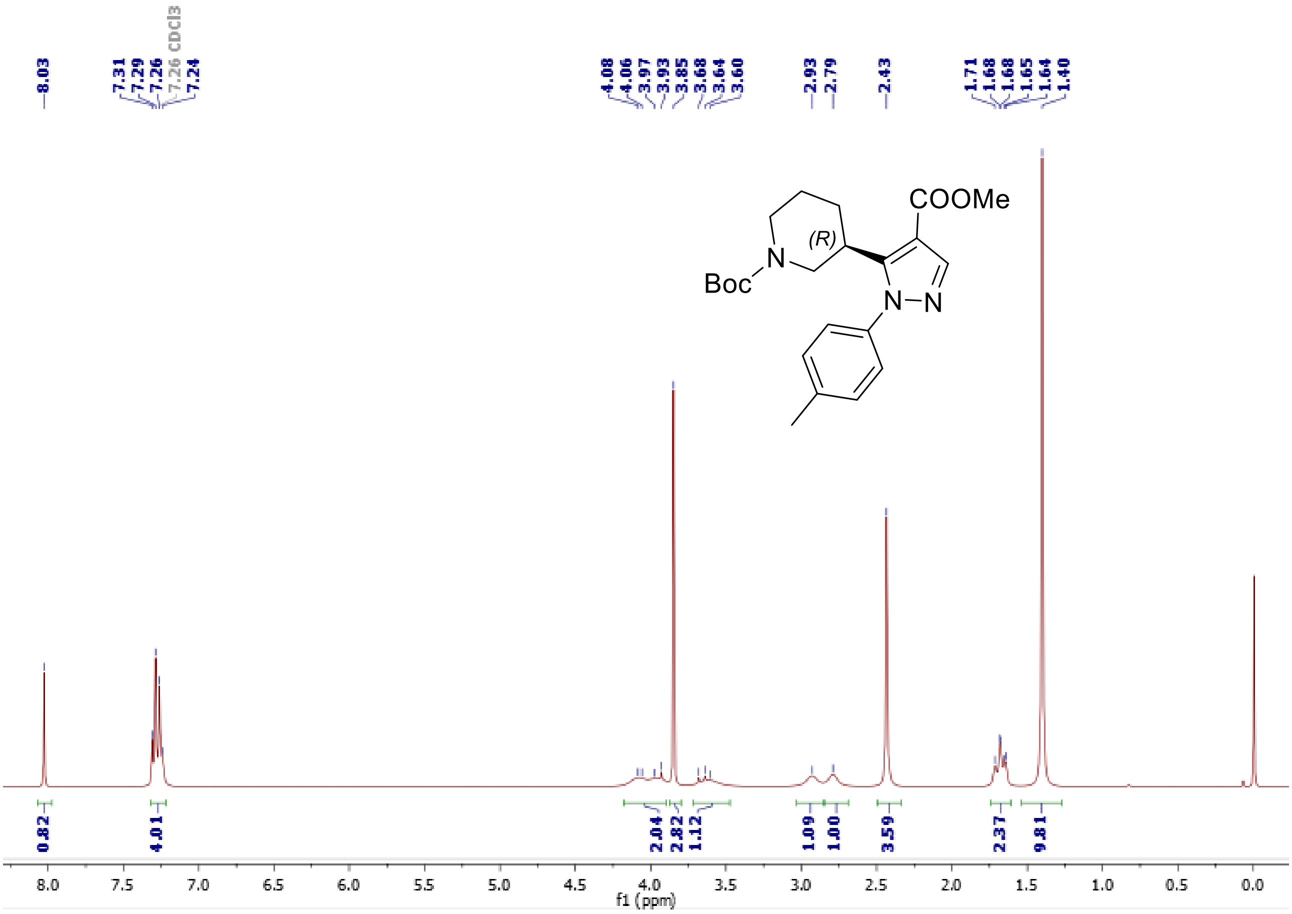

Figure S51. ***tert*-Butyl (3*R*)-3-[4-(methoxycarbonyl)-1-(4-methylphenyl)-1*H*-pyrazol-5-yl]piperidine-1-carboxylate (5l). ^{1}H NMR spectrum (400 MHz, $CDCl_3$).**

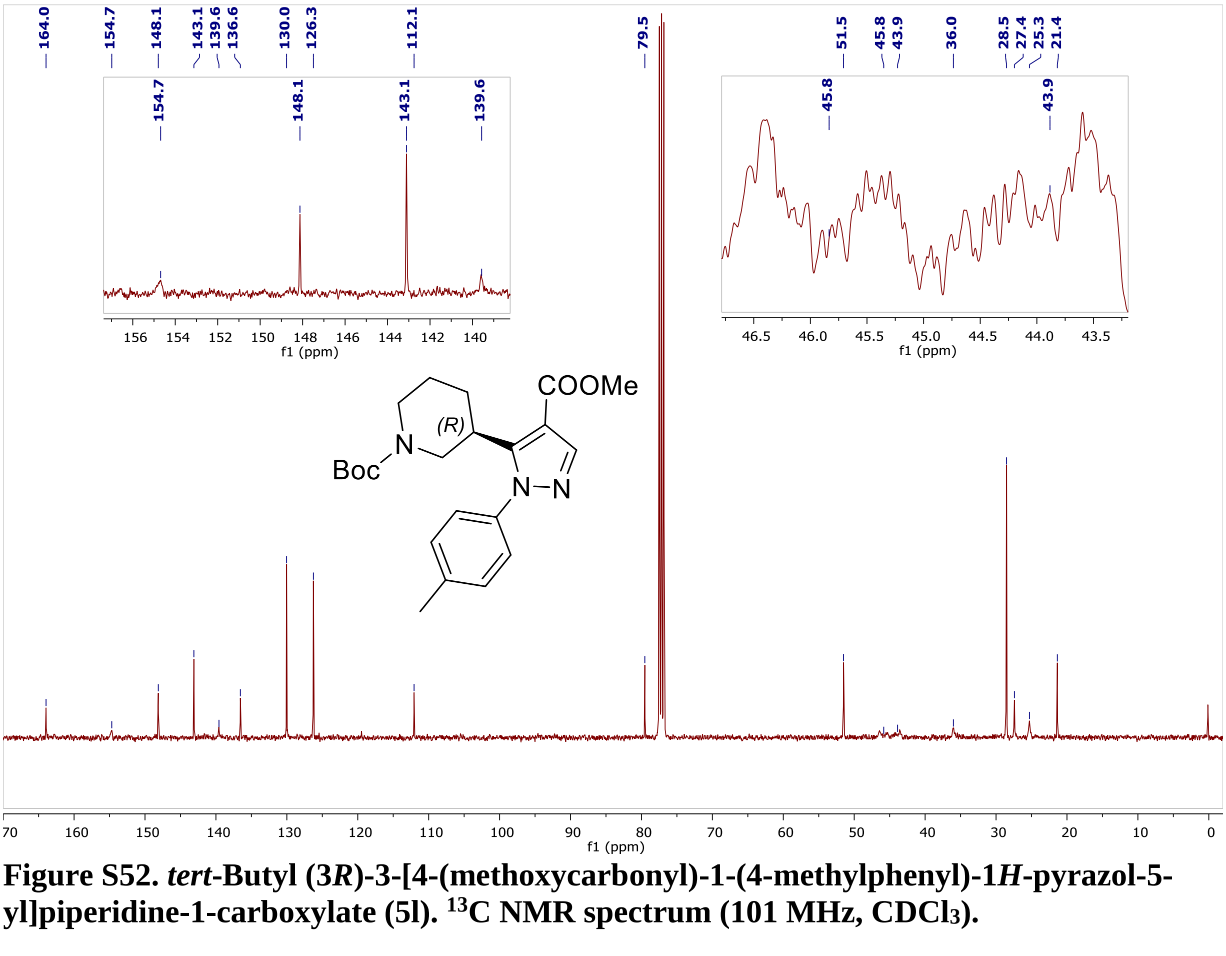

Figure S52. ***tert*-Butyl (3*R*)-3-[4-(methoxycarbonyl)-1-(4-methylphenyl)-1*H*-pyrazol-5-yl]piperidine-1-carboxylate (5l). ^{13}C NMR spectrum (101 MHz, $CDCl_3$).**

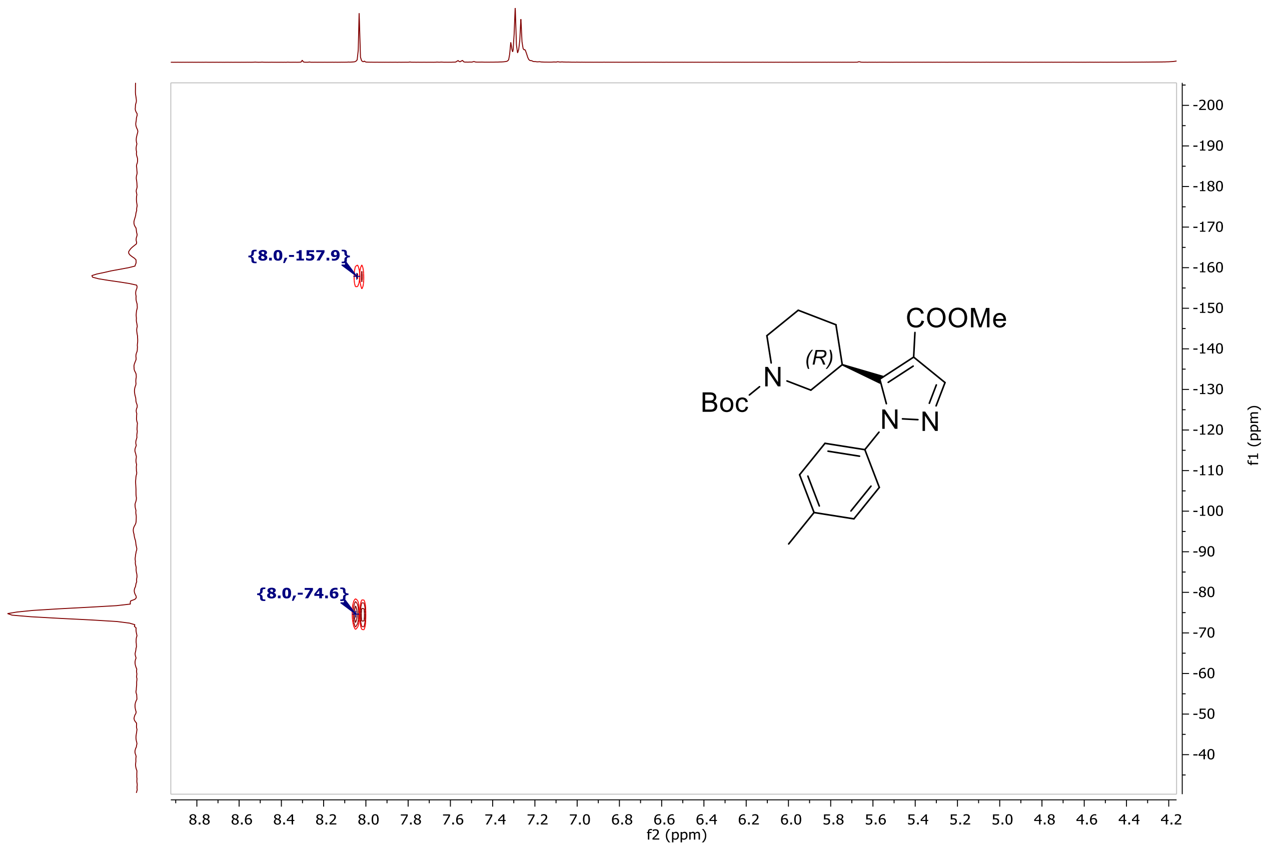

Figure S53. ***tert*-Butyl (3*R*)-3-[4-(methoxycarbonyl)-1-(4-methylphenyl)-1*H*-pyrazol-5-yl]piperidine-1-carboxylate (5l). ^{1}H-^{15}N HMBC spectrum (41 MHz, $CDCl_3$).**

Compound Spectrum SmartFormula Report

Analysis Info

Analysis Name	D:\Data\GMP-116.d	Acquisition Date	2/5/2021 12:09:00 AM
Method	DirectInfusion_TuneLow_pos.m	Operator	hplc
Sample Name	GMP-116	Instrument	micrOTOF-Q III 8228888.20448
Comment	AB		

Acquisition Parameter

Source Type	ESI	Ion Polarity	Positive	Set Nebulizer	0.4 Bar
Focus	Not active	Set Capillary	4500 V	Set Dry Heater	180 °C
Scan Begin	50 m/z	Set End Plate Offset	-500 V	Set Dry Gas	4.0 l/min
Scan End	1000 m/z	Set Collision Cell RF	140.0 Vpp	Set Divert Valve	Waste

#	RT [min]	Area	Int. Type	I	S/N	Chromatogram	Max. m/z	FWHM [min]
n.a.	3.3	n.a.	Single spectrum	n.a.	n.a.	n.a.	422.2052	n.a.

+MS, 3.3min #196

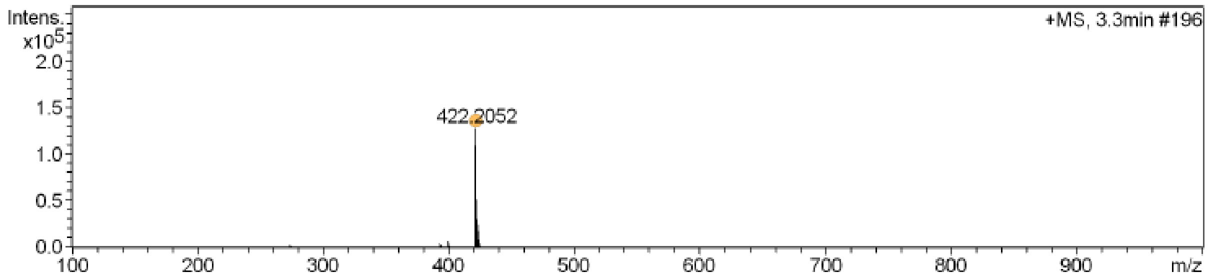

Meas. m/z	#	Ion Formula	m/z	err [ppm]	mSigma	# Sigma	Score	rdb	e⁻ Conf	N-Rule
422.2052	1	C22H29N3NaO4	422.2050	-0.3	3.3	1	100.00	9.5	even	ok

Figure S54. *tert*-Butyl (3*R*)-3-[4-(methoxycarbonyl)-1-(4-methylphenyl)-1*H*-pyrazol-5-yl]piperidine-1-carboxylate (5l). HRMS (ESI-TOF).

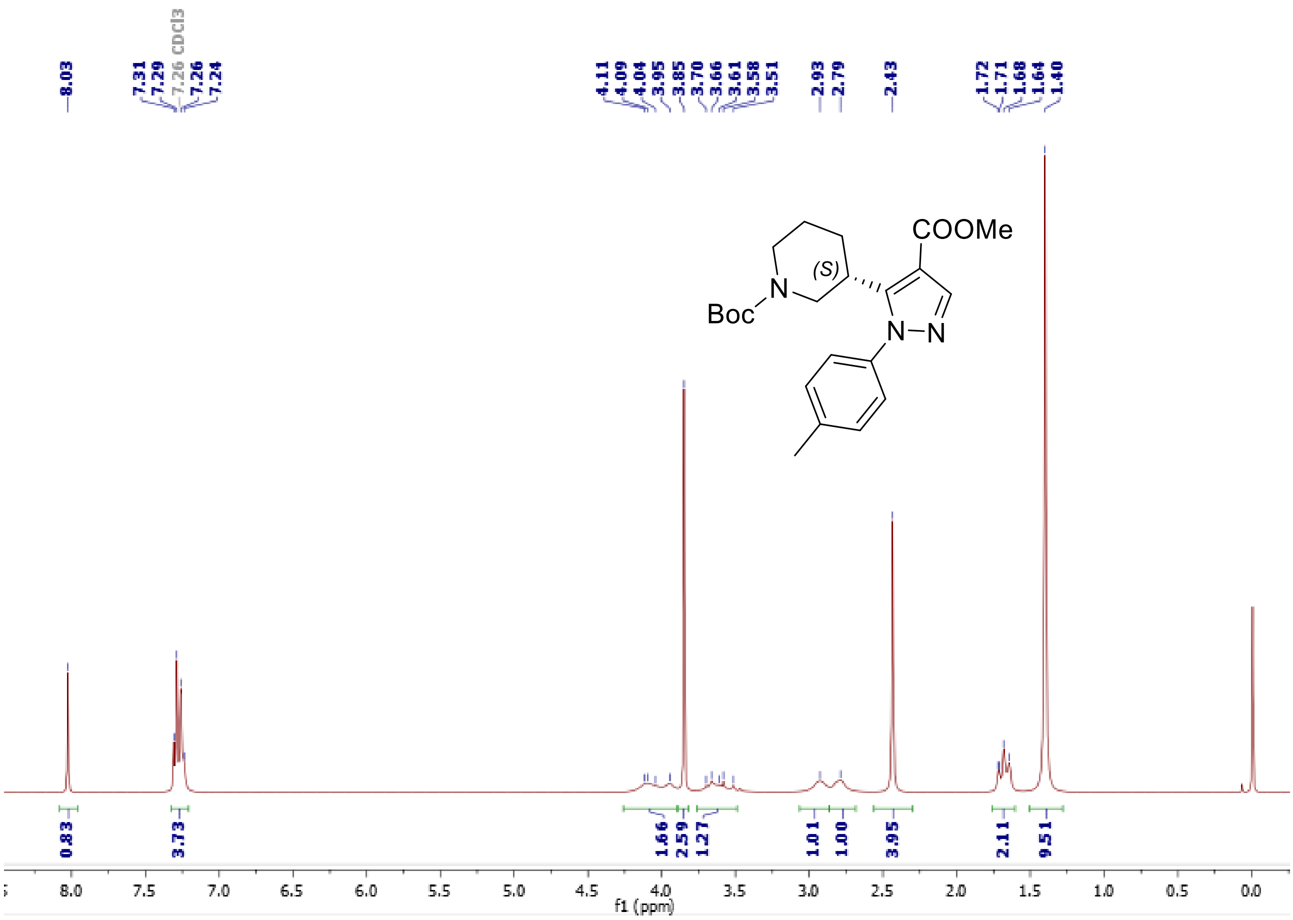

Figure S55. ***tert*-Butyl (3*S*)-3-[4-(methoxycarbonyl)-1-(4-methylphenyl)-1*H*-pyrazol-5-yl]piperidine-1-carboxylate (5m). ^{1}H NMR spectrum (400 MHz, $CDCl_3$).**

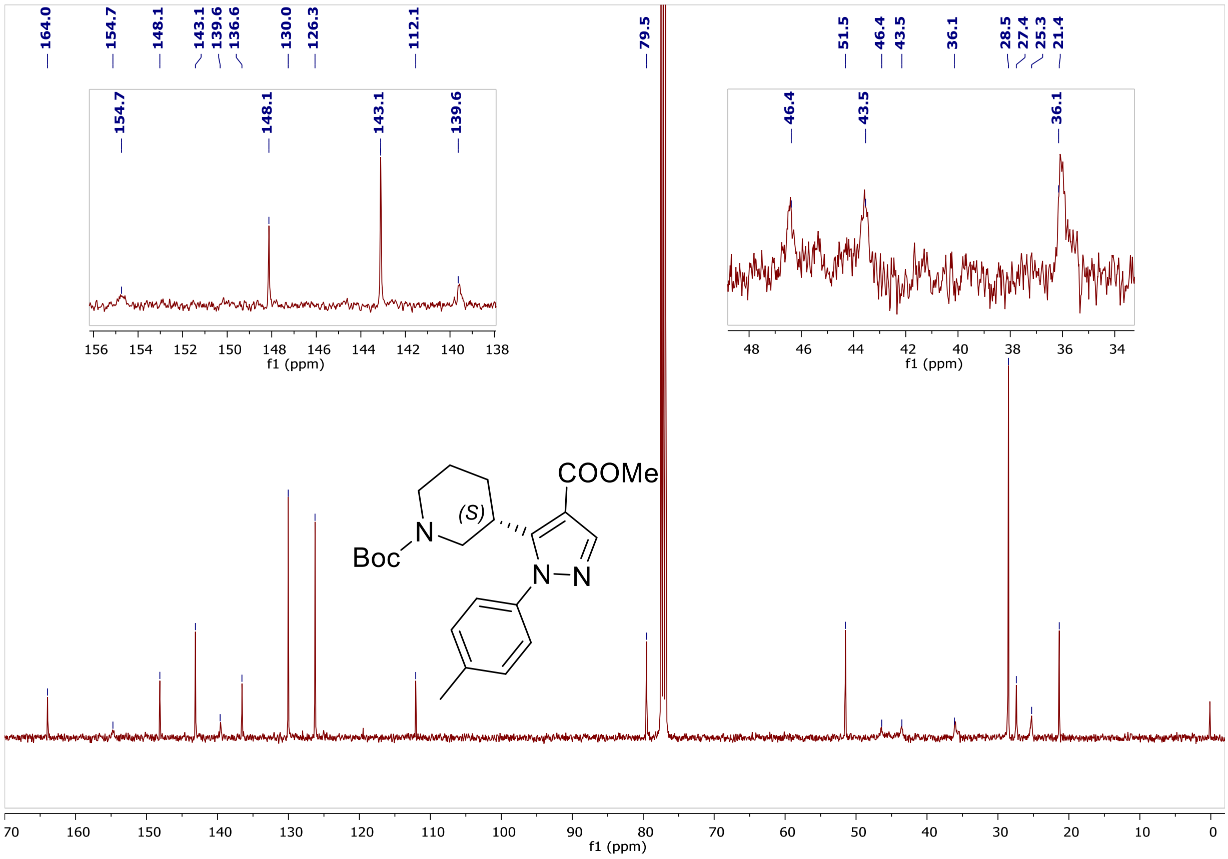

Figure S56. ***tert*-Butyl (3*S*)-3-[4-(methoxycarbonyl)-1-(4-methylphenyl)-1*H*-pyrazol-5-yl]piperidine-1-carboxylate (5m). ^{13}C NMR spectrum (101 MHz, $CDCl_3$).**

Compound Spectrum SmartFormula Report

Analysis Info

Analysis Name	D:\Data\GMP-147.d	Acquisition Date	2/4/2021 11:52:08 PM
Method	DirectInfusion_TuneLow_pos.m	Operator	hplc
Sample Name	GMP-147	Instrument	micrOTOF-Q III 8228888.20448
Comment	AB		

Acquisition Parameter

Source Type	ESI	Ion Polarity	Positive	Set Nebulizer	0.4 Bar
Focus	Not active	Set Capillary	4500 V	Set Dry Heater	180 °C
Scan Begin	50 m/z	Set End Plate Offset	-500 V	Set Dry Gas	4.0 l/min
Scan End	1000 m/z	Set Collision Cell RF	140.0 Vpp	Set Divert Valve	Waste

#	RT [min]	Area	Int. Type	I	S/N	Chromatogram	Max. m/z	FWHM [min]
n.a.	2.1	n.a.	Single spectrum	n.a.	n.a.	n.a.	422.2051	n.a.

+MS, 2.1min #126

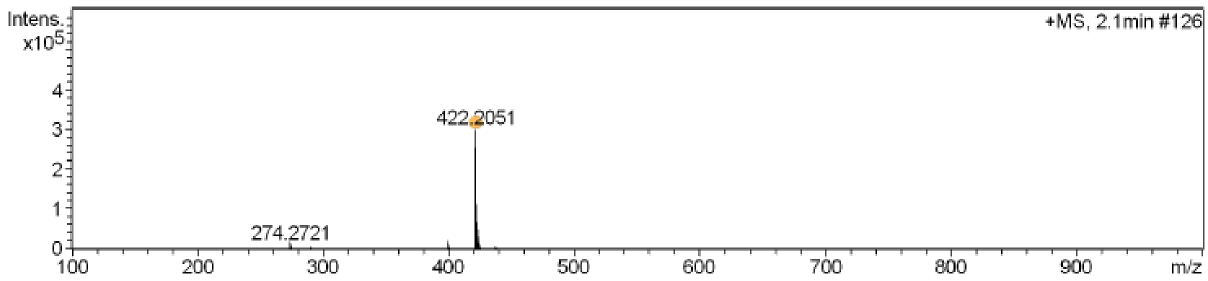

Meas. m/z	#	Ion Formula	m/z	err [ppm]	mSigma	# Sigma	Score	rdb	e⁻ Conf	N-Rule
422.2051	1	C22H29N3NaO4	422.2050	-0.3	4.4	1	100.00	9.5	even	ok

Figure S57. *tert*-Butyl (3*S*)-3-[4-(methoxycarbonyl)-1-(4-methylphenyl)-1*H*-pyrazol-5-yl]piperidine-1-carboxylate (5m). HRMS (ESI-TOF).

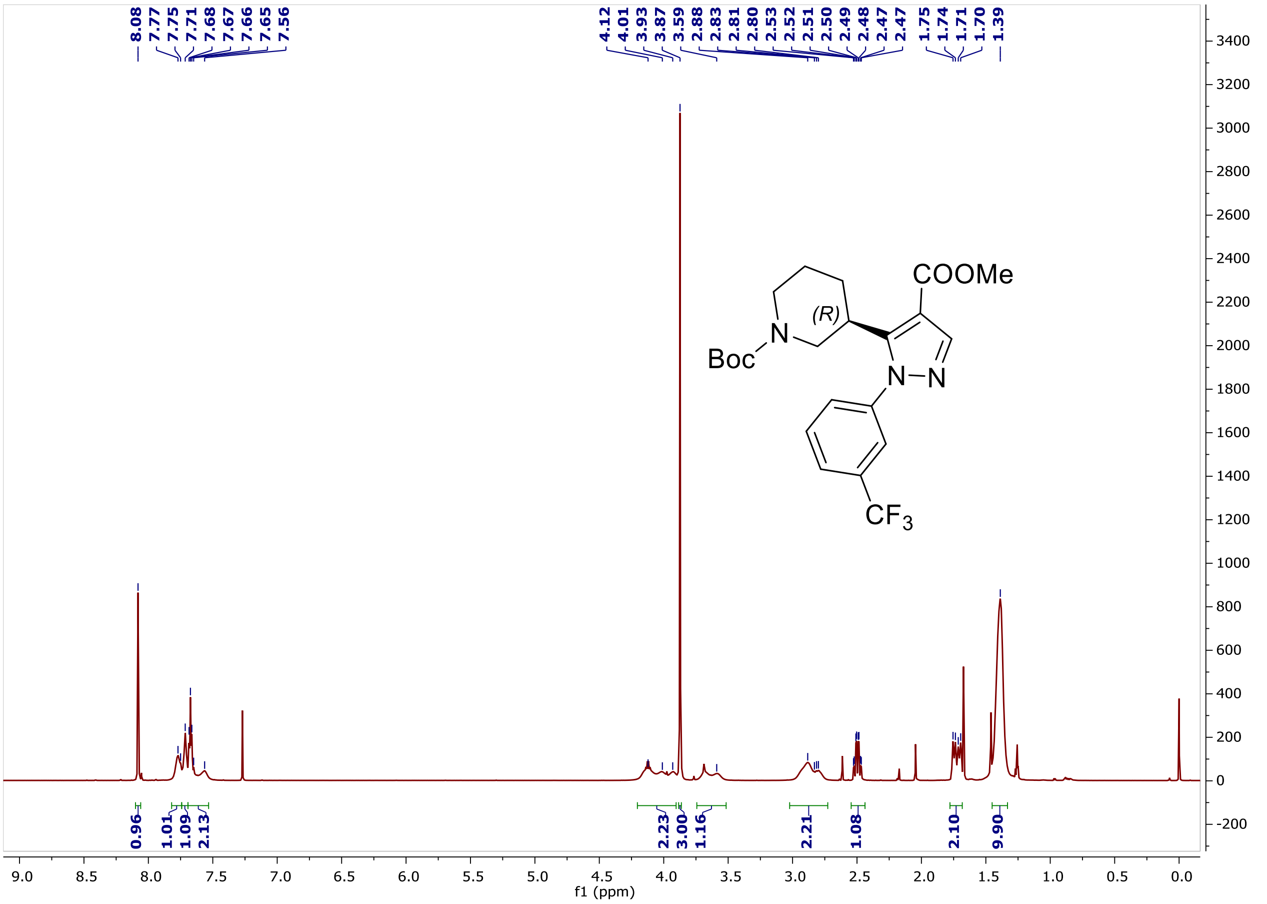

Figure S58. ***tert*-Butyl (3*R*)-3-{4-(methoxycarbonyl)-1-[3-(trifluoromethyl)phenyl]-1*H*-pyrazol-5-yl}piperidine-1-carboxylate (5n). ^{1}H NMR spectrum (700 MHz, $CDCl_3$).**

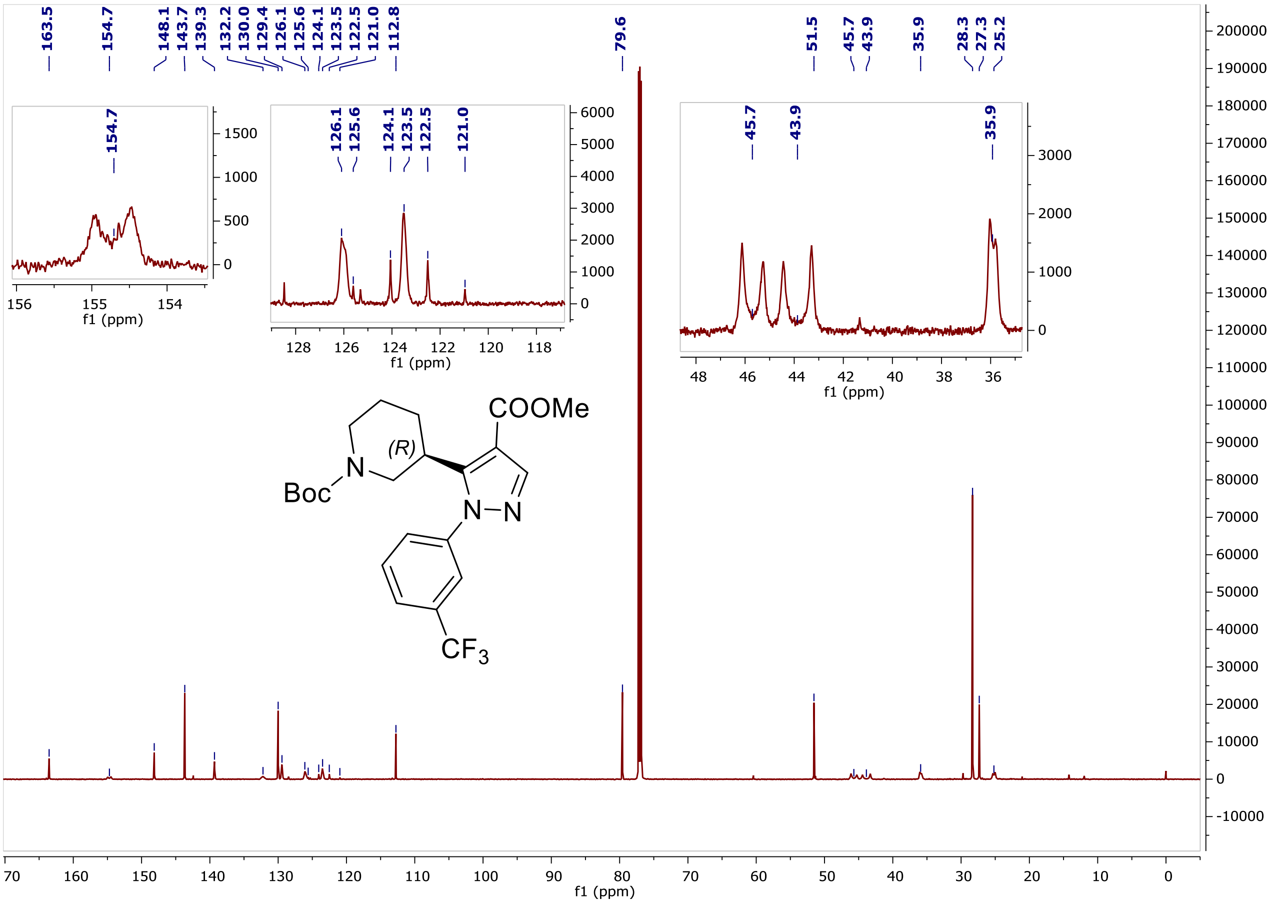

Figure S59. ***tert*-Butyl (3*R*)-3-{4-(methoxycarbonyl)-1-[3-(trifluoromethyl)phenyl]-1*H*-pyrazol-5-yl}piperidine-1-carboxylate (5n). ^{13}C NMR spectrum (176 MHz, $CDCl_3$).**

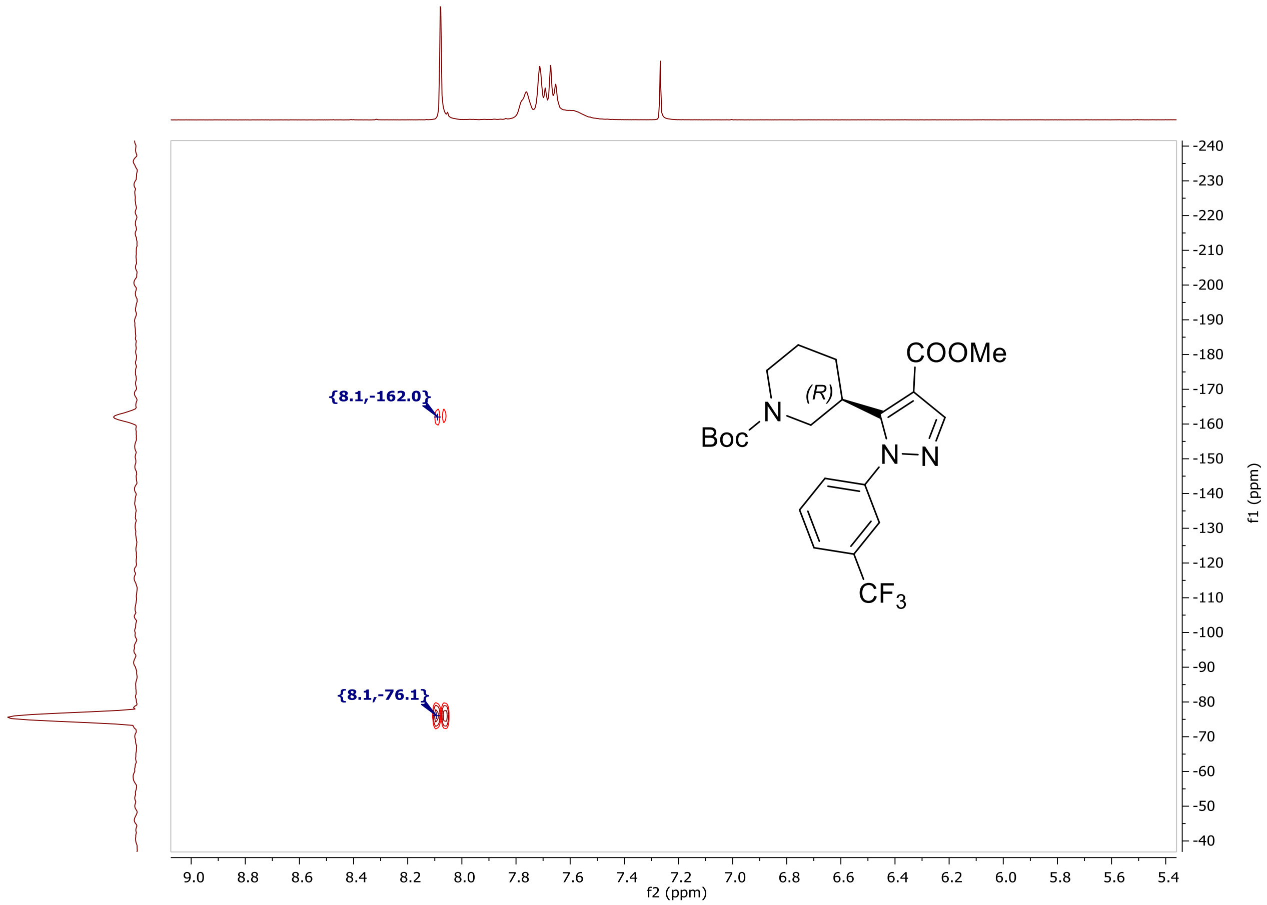

Figure S60. *tert*-Butyl (3*R*)-3-{4-(methoxycarbonyl)-1-[3-(trifluoromethyl)phenyl]-1*H*-pyrazol-5-yl}piperidine-1-carboxylate (5n). ^{1}H-^{15}N HMBC spectrum (41 MHz, $CDCl_3$).

Compound Spectrum SmartFormula Report

Analysis Info

Analysis Name	D:\Data\GMP-115.d	Acquisition Date	2/5/2021 1:01:16 AM
Method	DirectInfusion_TuneLow_pos.m	Operator	hplc
Sample Name	GMP-115	Instrument	micrOTOF-Q III 8228888.20448
Comment	AB		

Acquisition Parameter

Source Type	ESI	Ion Polarity	Positive	Set Nebulizer	0.4 Bar
Focus	Not active	Set Capillary	4500 V	Set Dry Heater	180 °C
Scan Begin	50 m/z	Set End Plate Offset	-500 V	Set Dry Gas	4.0 l/min
Scan End	1000 m/z	Set Collision Cell RF	140.0 Vpp	Set Divert Valve	Waste

#	RT [min]	Area	Int. Type	I	S/N	Chromatogram	Max. m/z	FWHM [min]
n.a.	9.4	n.a.	Single spectrum	n.a.	n.a.	n.a.	476.1769	n.a.

+MS, 9.4min #565

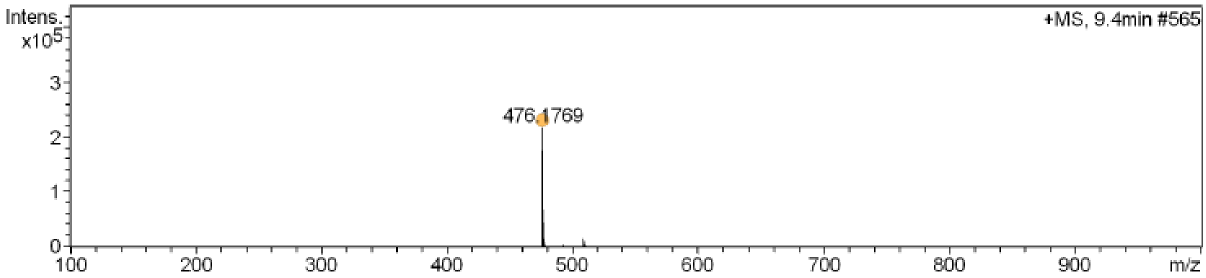

Meas. m/z	#	Ion Formula	m/z	err [ppm]	mSigma	# Sigma	Score	rdb	e⁻ Conf	N-Rule
476.1769	1	C22H26F3N3NaO4	476.1768	-0.3	3.4	3	100.00	9.5	even	ok

Figure S61. ***tert*-Butyl (3*R*)-3-{4-(methoxycarbonyl)-1-[3-(trifluoromethyl)phenyl]-1*H*-pyrazol-5-yl}piperidine-1-carboxylate (5n). HRMS (ESI-TOF).**

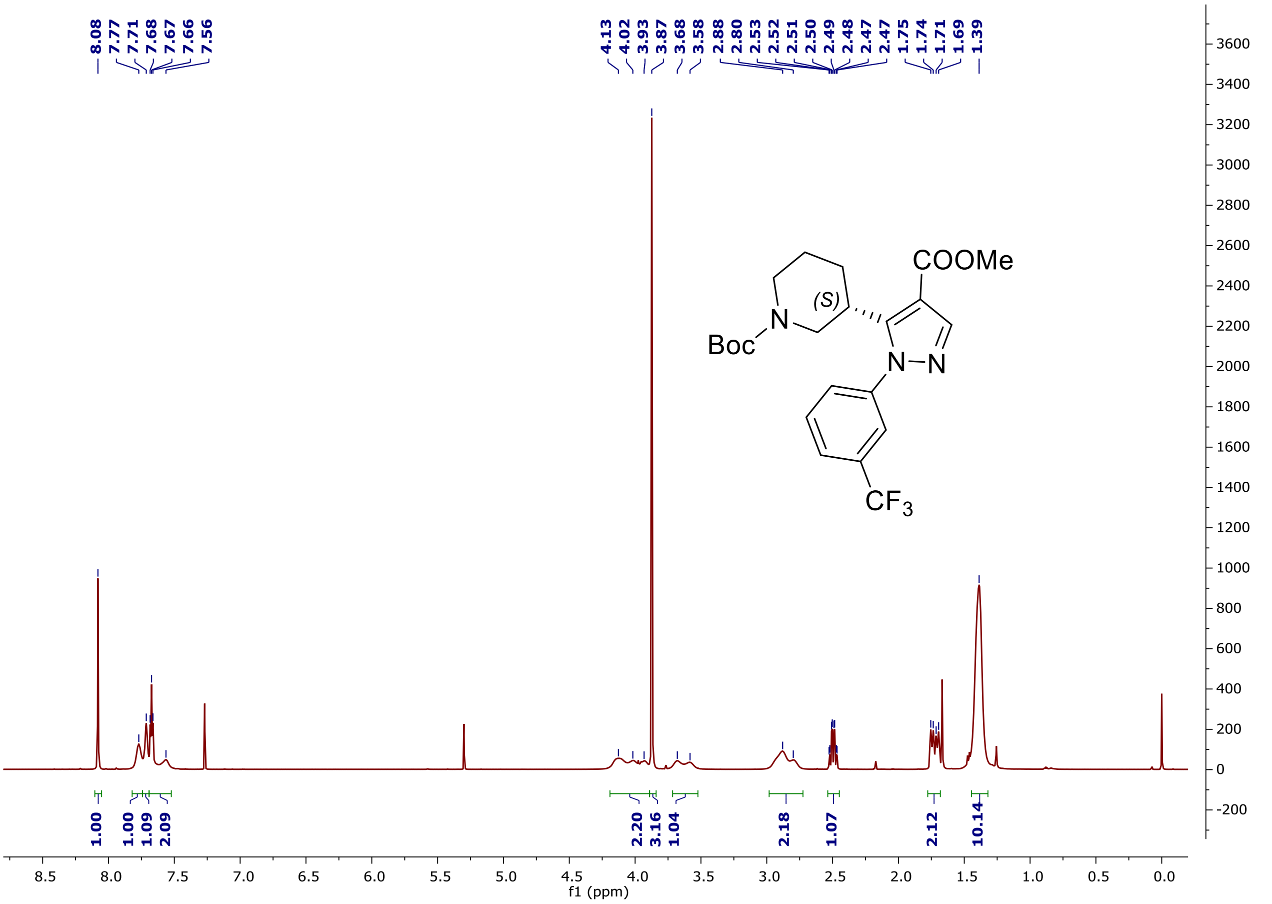

Figure S62. ***tert*-Butyl (3*S*)-3-{4-(methoxycarbonyl)-1-[3-(trifluoromethyl)phenyl]-1*H*-pyrazol-5-yl}piperidine-1-carboxylate (5o). ^{1}H NMR spectrum (700 MHz, CDCl$_3$).**

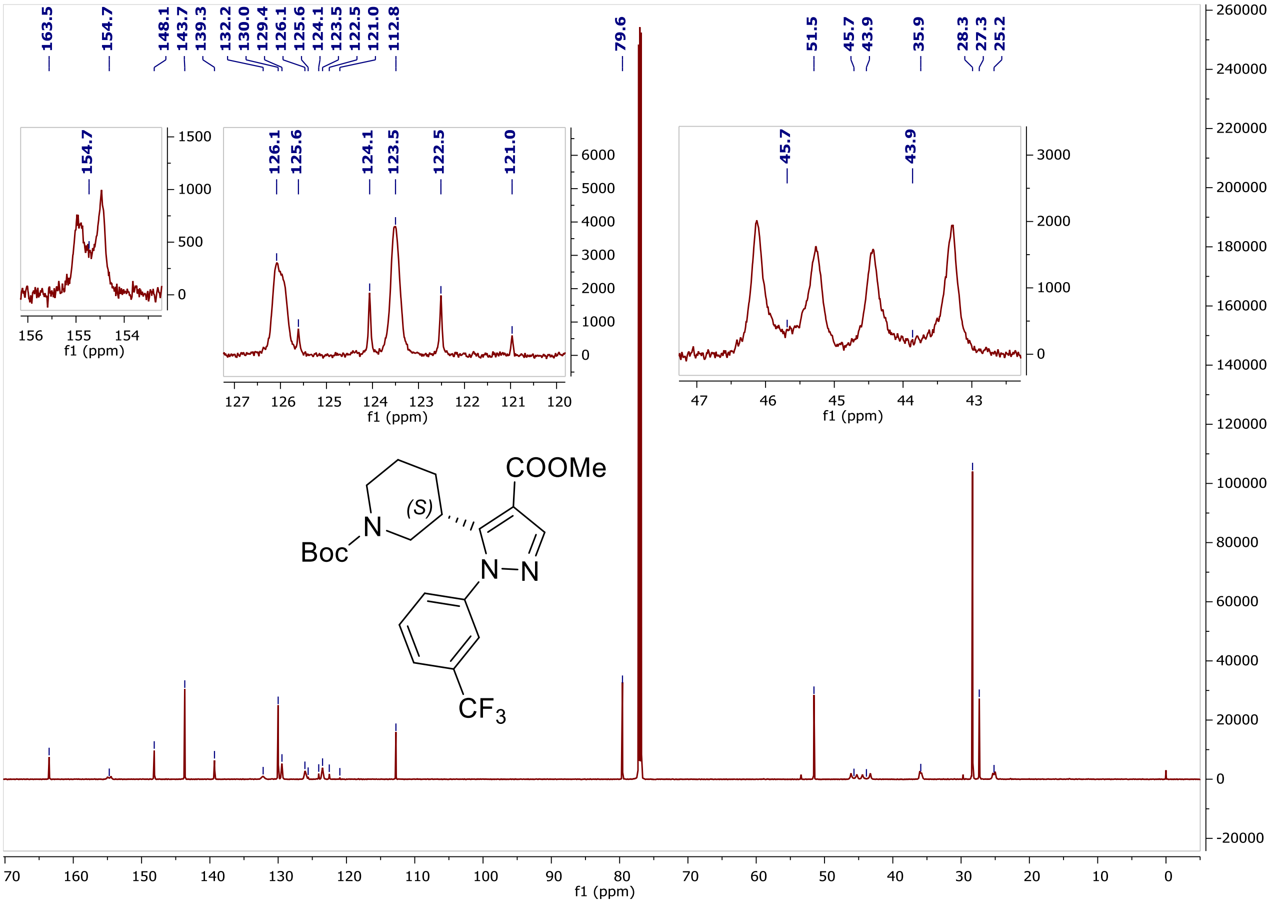

Figure S63. ***tert*-Butyl (3*S*)-3-{4-(methoxycarbonyl)-1-[3-(trifluoromethyl)phenyl]-1*H*-pyrazol-5-yl}piperidine-1-carboxylate (5o). ^{13}C NMR spectrum (176 MHz, CDCl$_3$).**

Compound Spectrum SmartFormula Report

Analysis Info

Analysis Name	D:\Data\GMP-148.d	Acquisition Date	2/4/2021 8:10:28 PM
Method	DirectInfusion_TuneLow_pos.m	Operator	hplc
Sample Name	GMP-148	Instrument	micrOTOF-Q III 8228888.20448
Comment	AB		

Acquisition Parameter

Source Type	ESI	Ion Polarity	Positive	Set Nebulizer	0.4 Bar
Focus	Not active	Set Capillary	4500 V	Set Dry Heater	180 °C
Scan Begin	50 m/z	Set End Plate Offset	-500 V	Set Dry Gas	4.0 l/min
Scan End	1000 m/z	Set Collision Cell RF	140.0 Vpp	Set Divert Valve	Waste

#	RT [min]	Area	Int. Type	I	S/N	Chromatogram	Max. m/z	FWHM [min]
n.a.	9.2	n.a.	Single spectrum	n.a.	n.a.	n.a.	476.1772	n.a.

+MS, 9.2min #552

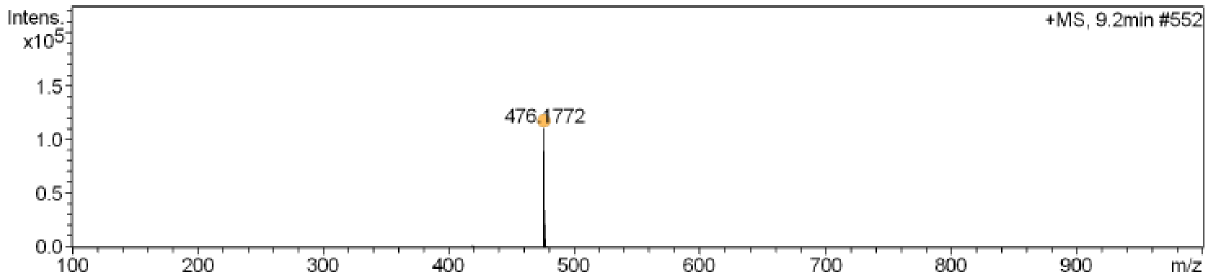

Meas. m/z	#	Ion Formula	m/z	err [ppm]	mSigma	# Sigma	Score	rdb	e¯ Conf	N-Rule
476.1772	1	C22H26F3N3NaO4	476.1768	-1.0	8.8	2	100.00	9.5	even	ok

Figure S64. ***tert*****-Butyl (3*S*)-3-{4-(methoxycarbonyl)-1-[3-(trifluoromethyl)phenyl]-1*H*-pyrazol-5-yl}piperidine-1-carboxylate (5o). HRMS (ESI-TOF).**

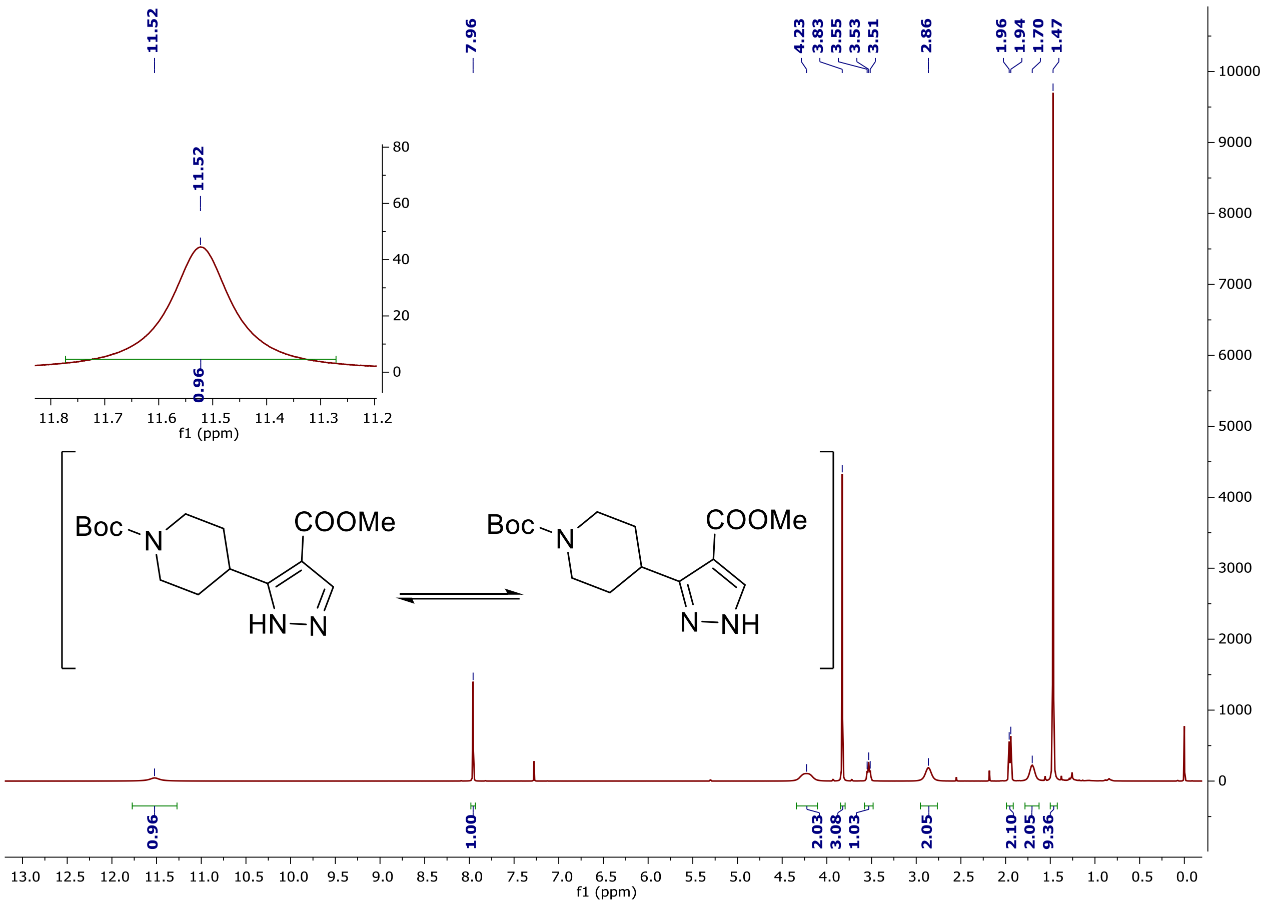

Figure S65. ***tert*-Butyl 4-[4-(methoxycarbonyl)-1*H*-pyrazolyl]piperidine-1-carboxylate (7). ^{1}H NMR spectrum (700 MHz, $CDCl_3$).**

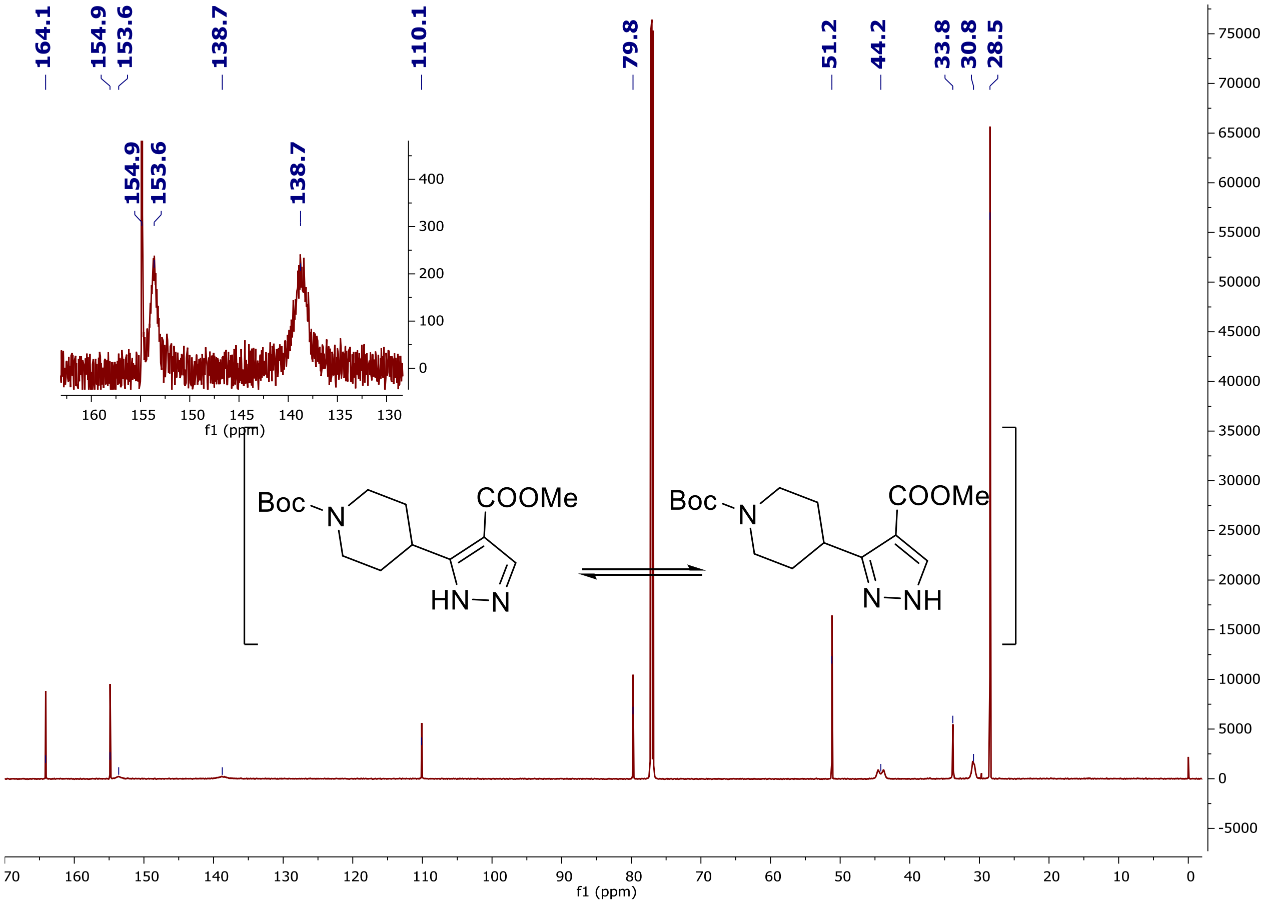

Figure S66. ***tert*-Butyl 4-[4-(methoxycarbonyl)-1*H*-pyrazolyl]piperidine-1-carboxylate (7). ^{13}C NMR spectrum (176 MHz, $CDCl_3$).**

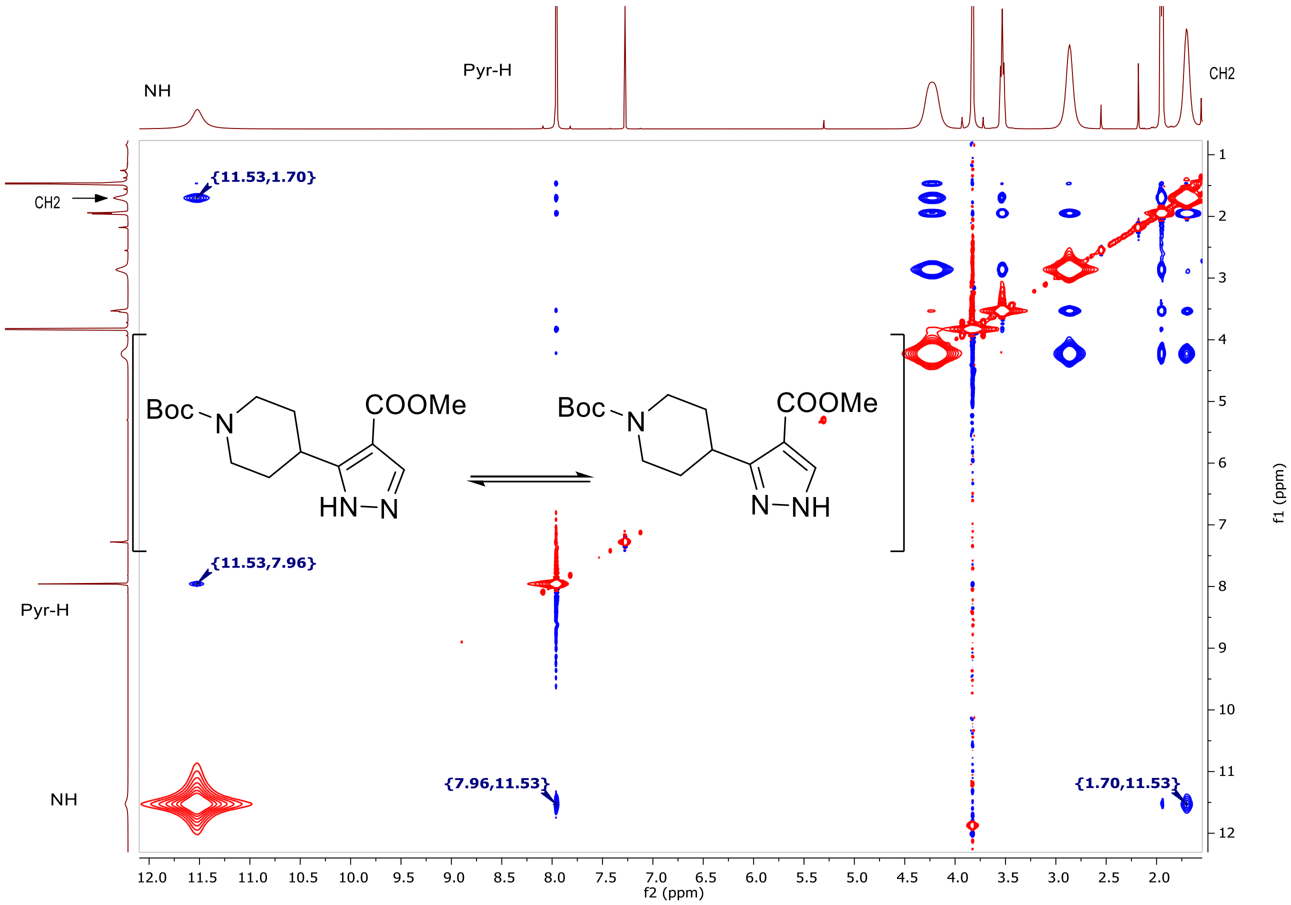

Figure S67. ***tert*-Butyl 4-[4-(methoxycarbonyl)-1*H*-pyrazolyl]piperidine-1-carboxylate (7). ^{1}H-^{1}H NOESY spectrum (CDCl$_3$).**

Compound Spectrum SmartFormula Report

Analysis Info

Analysis Name	D:\Data\GMP-018.d	Acquisition Date	11/27/2018 2:33:41 PM
Method	DirectInfusion_TuneLow_pos.m	Operator	hplc
Sample Name	GMP-018	Instrument	micrOTOF-Q III 8228888.20448
Comment	SB		

Acquisition Parameter

Source Type	ESI	Ion Polarity	Positive	Set Nebulizer	0.4 Bar
Focus	Not active	Set Capillary	4500 V	Set Dry Heater	180 °C
Scan Begin	50 m/z	Set End Plate Offset	-500 V	Set Dry Gas	4.0 l/min
Scan End	1000 m/z	Set Collision Cell RF	140.0 Vpp	Set Divert Valve	Waste

#	RT [min]	Area	Int. Type	I	S/N	Chromatogram	Max. m/z	FWHM [min]
n.a.	0.2	n.a.	Single spectrum	n.a.	n.a.	n.a.	304.2632	n.a.
n.a.	4.3	n.a.	Single spectrum	n.a.	n.a.	n.a.	332.1581	n.a.

+MS, 4.3min #255

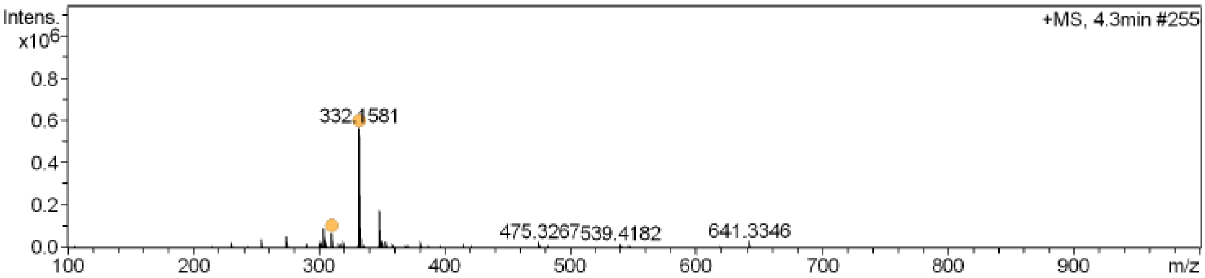

Meas. m/z	#	Ion Formula	m/z	err [ppm]	mSigma	# Sigma	Score	rdb	e⁻ Conf	N-Rule
310.1759	1	C15H24N3O4	310.1761	-0.8	4.2	1	100.00	5.5	even	ok
332.1581	1	C15H23N3NaO4	332.1581	0.1	1.0	1	100.00	5.5	even	ok

Figure S68. *tert*-Butyl 4-[4-(methoxycarbonyl)-1*H*-pyrazolyl]piperidine-1-carboxylate (7). HRMS (ESI-TOF).

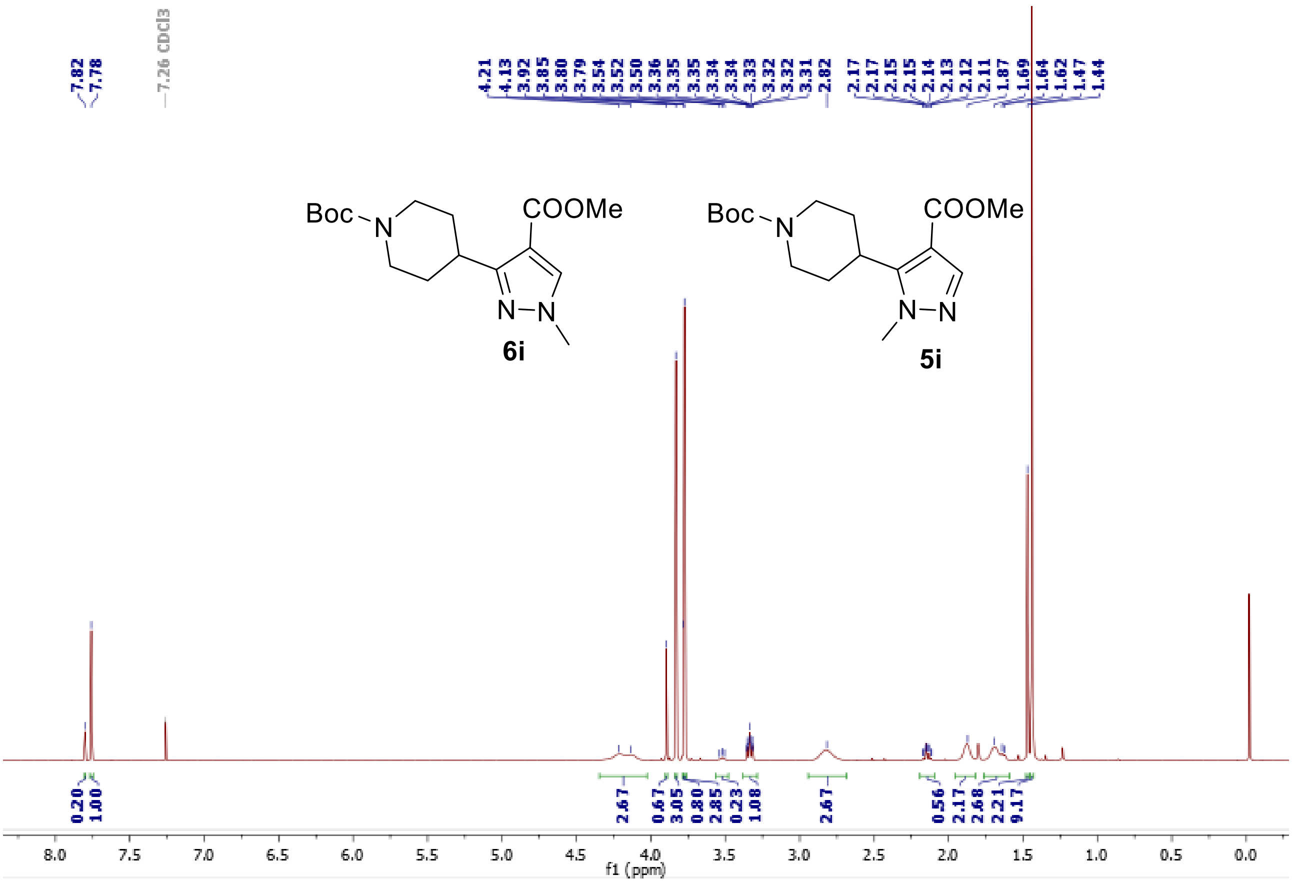

Figure S69. ***tert*-Butyl 4-[4-(methoxycarbonyl)-1-methyl-1*H*-pyrazol-3-yl]piperidine-1-carboxylate (6i) and *tert*-butyl 4-[4-(methoxycarbonyl)-1-methyl-1*H*-pyrazol-5-yl]piperidine-1-carboxylate (5i). ^{1}H NMR spectrum (700 MHz, $CDCl_3$).**

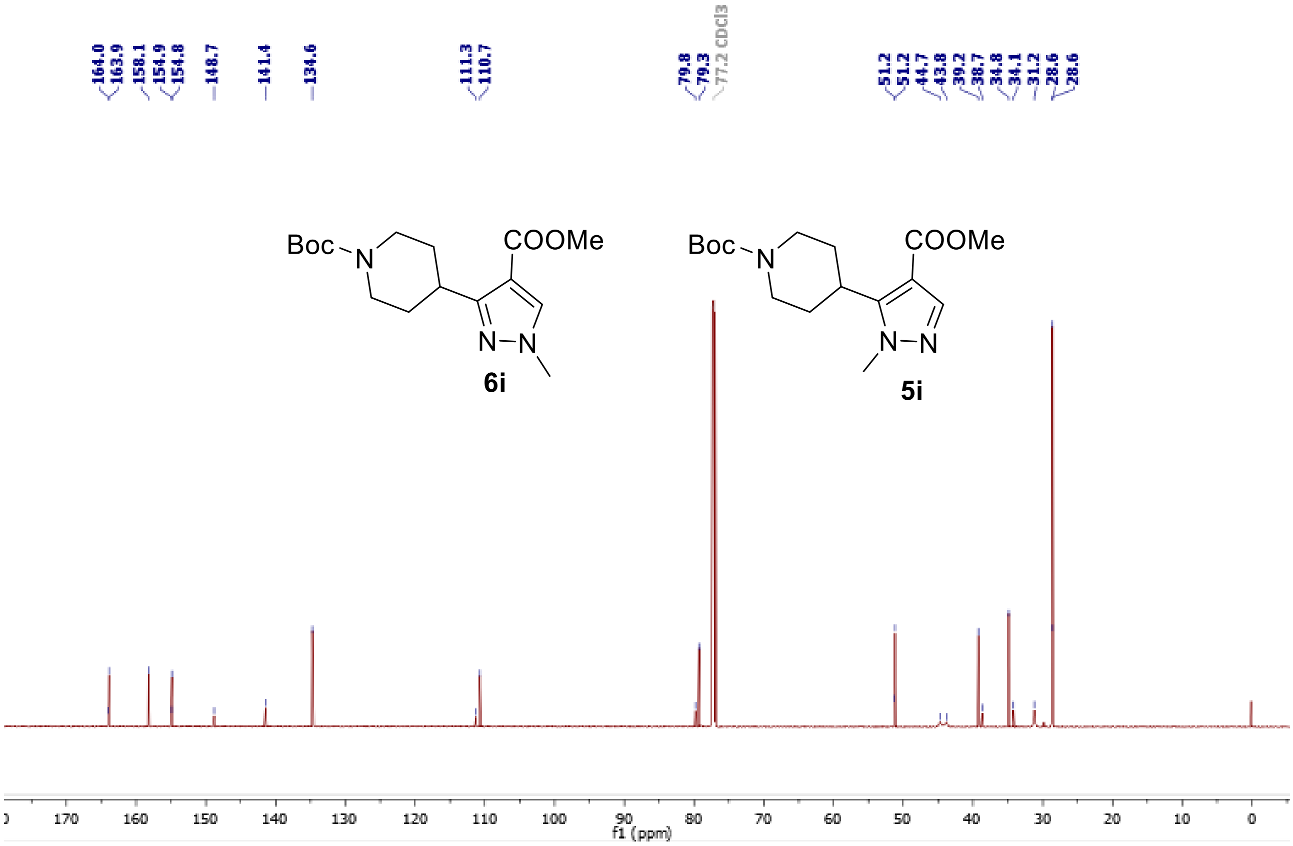

Figure S70. ***tert*-Butyl 4-[4-(methoxycarbonyl)-1-methyl-1*H*-pyrazol-3-yl]piperidine-1-carboxylate (6i) and *tert*-butyl 4-[4-(methoxycarbonyl)-1-methyl-1*H*-pyrazol-5-yl]piperidine-1-carboxylate (5i). ^{13}C NMR spectrum (176 MHz, $CDCl_3$).**

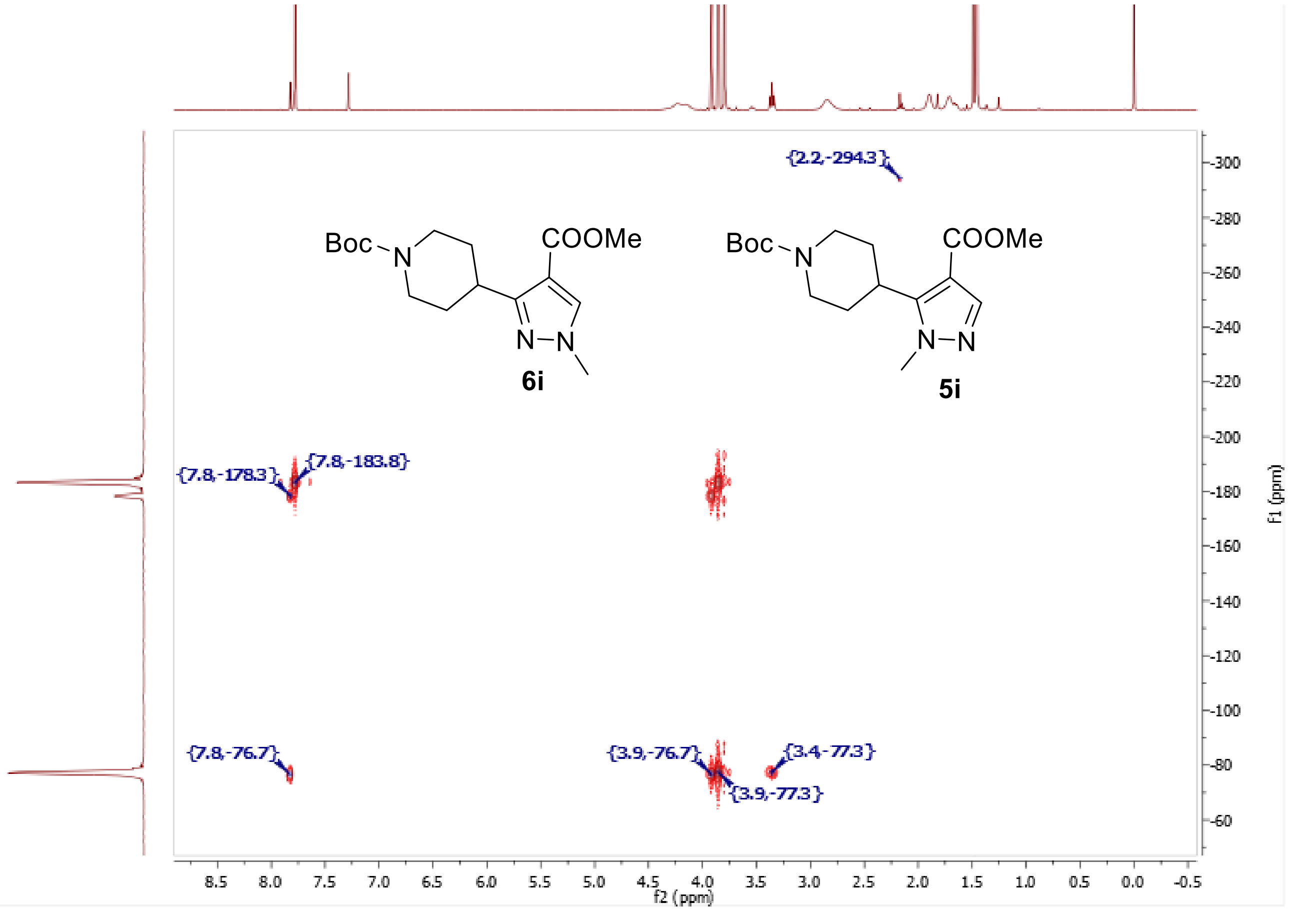

Figure S71. ***tert*-Butyl 4-[4-(methoxycarbonyl)-1-methyl-1*H*-pyrazol-3-yl]piperidine-1-carboxylate (6i) and *tert*-butyl 4-[4-(methoxycarbonyl)-1-methyl-1*H*-pyrazol-5-yl]piperidine-1-carboxylate (5i). ^{1}H-^{15}N HMBC spectrum (71 MHz, $CDCl_3$).**

Compound Spectrum SmartFormula Report

Analysis Info

Analysis Name	D:\Data\GMP-517.d	Acquisition Date	2/4/2021 11:12:05 PM
Method	DirectInfusion_TuneLow_pos.m	Operator	hplc
Sample Name	GMP-517	Instrument	micrOTOF-Q III 8228888.20448
Comment	AB		

Acquisition Parameter

Source Type	ESI	Ion Polarity	Positive	Set Nebulizer	0.4 Bar
Focus	Not active	Set Capillary	4500 V	Set Dry Heater	180 °C
Scan Begin	50 m/z	Set End Plate Offset	-500 V	Set Dry Gas	4.0 l/min
Scan End	1000 m/z	Set Collision Cell RF	140.0 Vpp	Set Divert Valve	Waste

#	RT [min]	Area	Int. Type	I	S/N	Chromatogram	Max. m/z	FWHM [min]
n.a.	6.9	n.a.	Single spectrum	n.a.	n.a.	n.a.	346.1737	n.a.

+MS, 6.9min #411

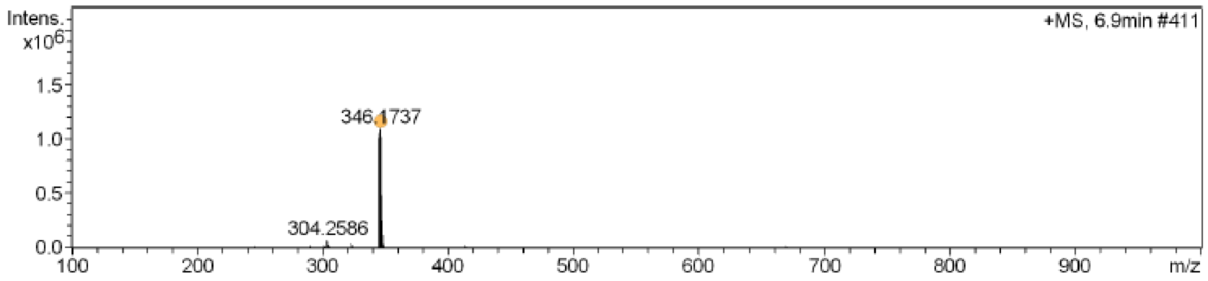

Meas. m/z	#	Ion Formula	m/z	err [ppm]	mSigma	# Sigma	Score	rdb	e⁻ Conf	N-Rule
346.1737	1	C16H25N3NaO4	346.1737	-0.1	37.5	1	100.00	5.5	even	ok

Figure S72. ***tert*-Butyl 4-[4-(methoxycarbonyl)-1-methyl-1*H*-pyrazol-3-yl]piperidine-1-carboxylate (6i) and *tert*-butyl 4-[4-(methoxycarbonyl)-1-methyl-1*H*-pyrazol-5-yl]piperidine-1-carboxylate (5i). HRMS (ESI-TOF).**

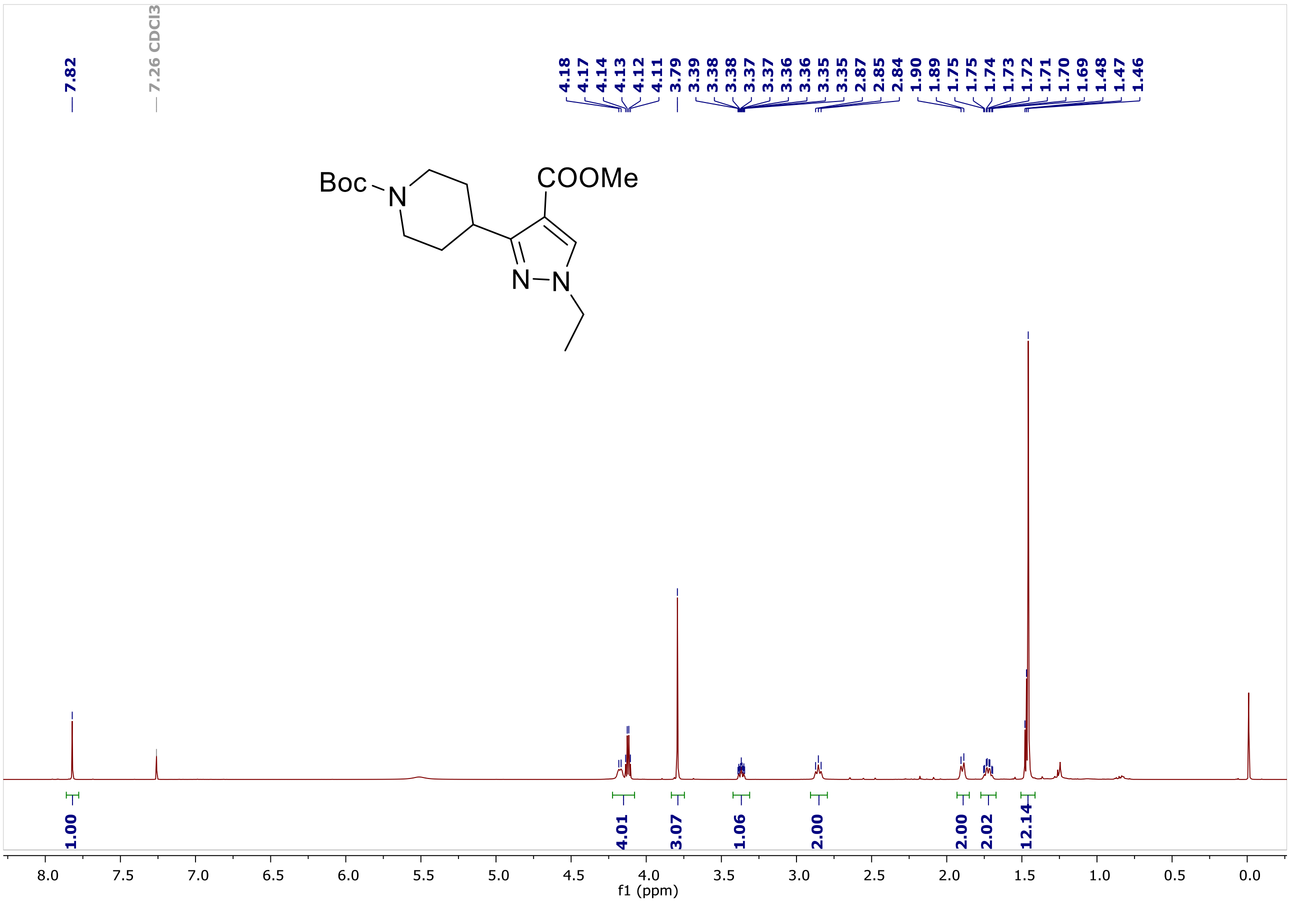

Figure S73. ***tert*-Butyl 4-[1-ethyl-4-(methoxycarbonyl)-1*H*-pyrazol-3-yl]piperidine-1-carboxylate (8). ^{1}H NMR spectrum (700 MHz, $CDCl_3$).**

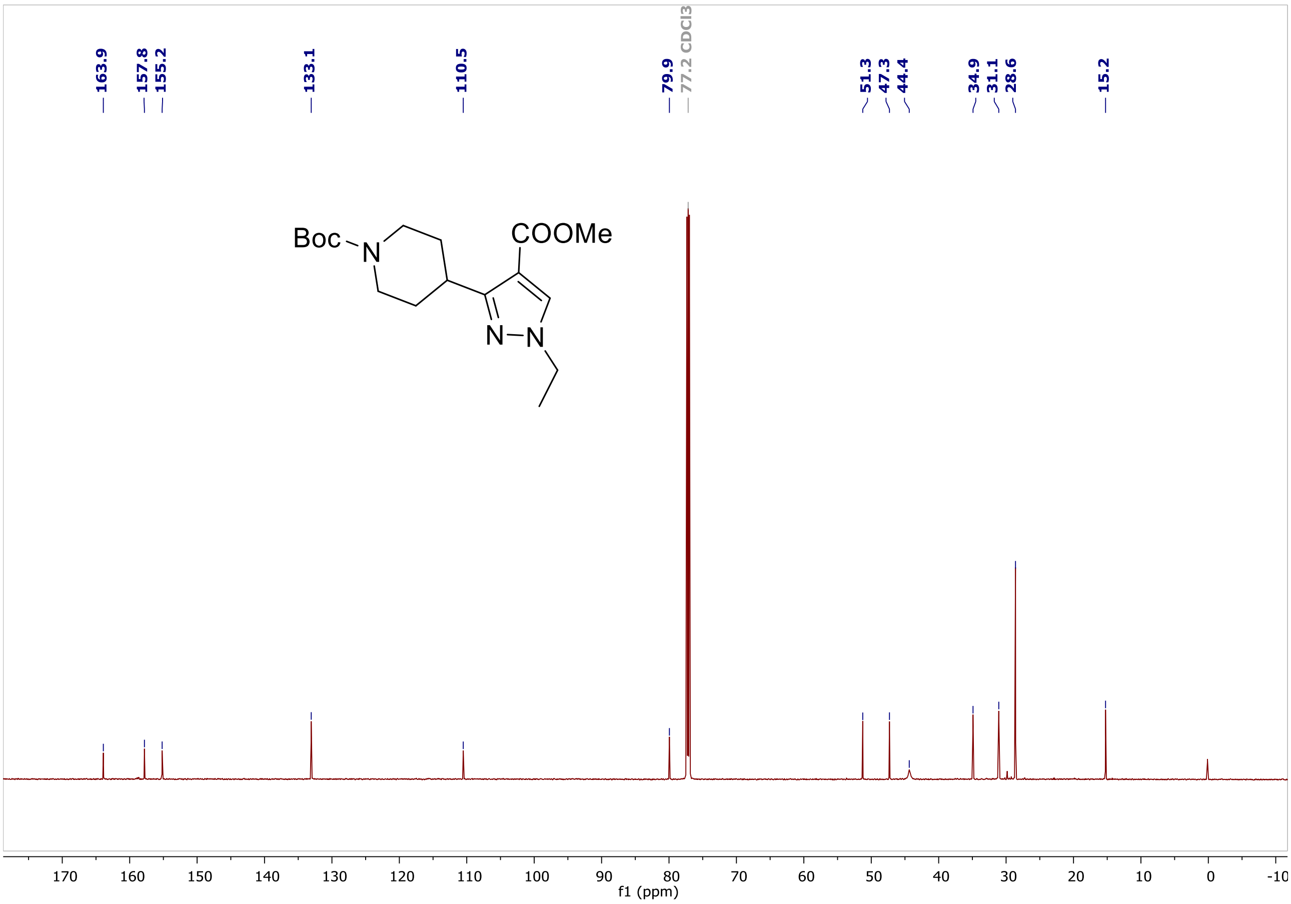

Figure S74. ***tert*-Butyl 4-[1-ethyl-4-(methoxycarbonyl)-1*H*-pyrazol-3-yl]piperidine-1-carboxylate (8). ^{13}C NMR spectrum (176 MHz, $CDCl_3$).**

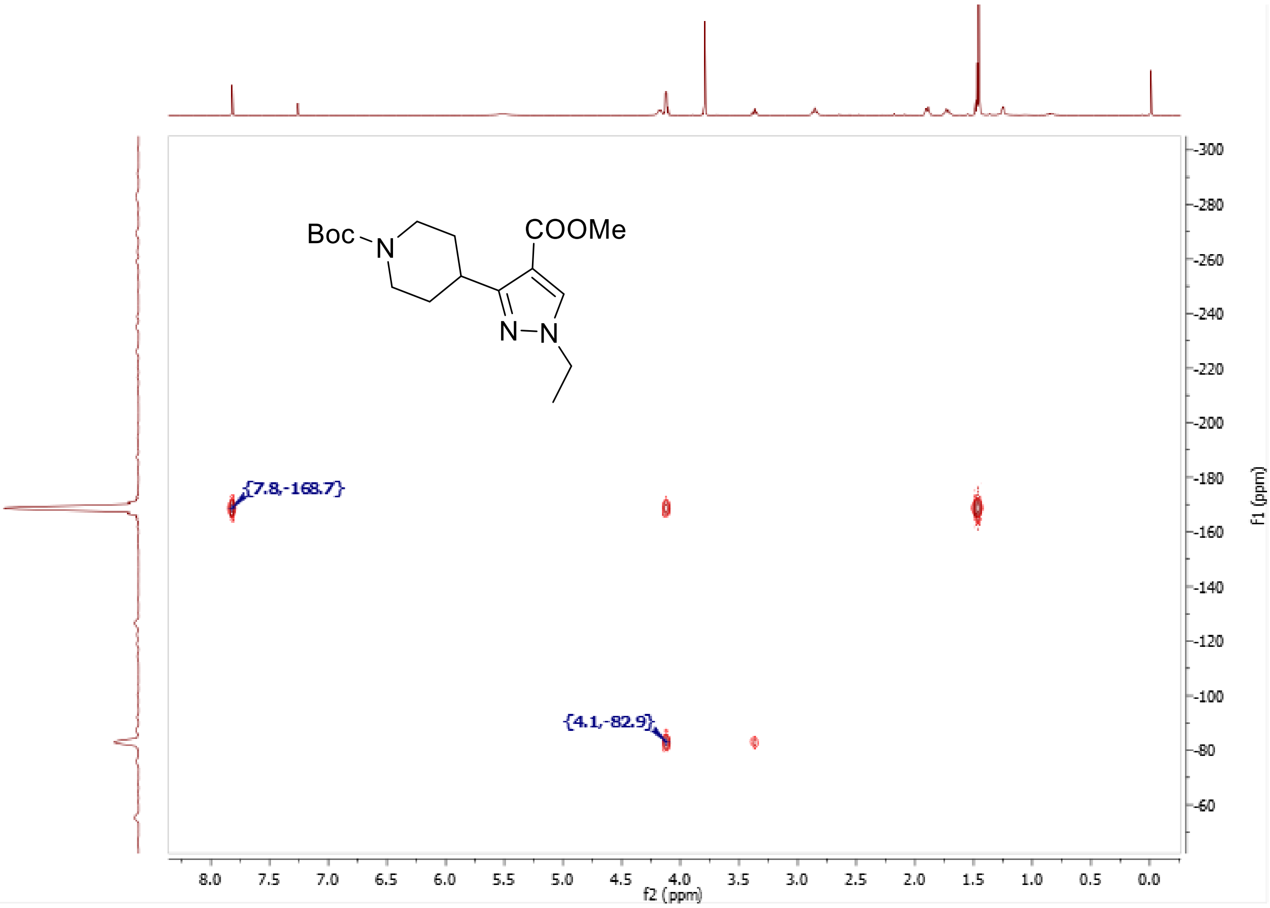

Figure S75. ***tert*-Butyl 4-[1-ethyl-4-(methoxycarbonyl)-1*H*-pyrazol-3-yl]piperidine-1-carboxylate (8). ^{1}H-^{15}N HMBC spectrum (71 MHz, CDCl$_3$).**

Compound Spectrum SmartFormula Report

Analysis Info

Analysis Name	D:\Data\GMP-019.d	Acquisition Date	11/27/2018 2:44:43 PM
Method	DirectInfusion_TuneLow_pos.m	Operator	hplc
Sample Name	GMP-019	Instrument	micrOTOF-Q III 8228888.20448
Comment	SB		

Acquisition Parameter

Source Type	ESI	Ion Polarity	Positive	Set Nebulizer	0.4 Bar
Focus	Not active	Set Capillary	4500 V	Set Dry Heater	180 °C
Scan Begin	50 m/z	Set End Plate Offset	-500 V	Set Dry Gas	4.0 l/min
Scan End	1000 m/z	Set Collision Cell RF	140.0 Vpp	Set Divert Valve	Waste

#	RT [min]	Area	Int. Type	I	S/N	Chromatogram	Max. m/z	FWHM [min]
n.a.	0.3	n.a.	Single spectrum	n.a.	n.a.	n.a.	304.2634	n.a.
n.a.	3.4	n.a.	Single spectrum	n.a.	n.a.	n.a.	360.1894	n.a.

+MS, 3.4min #206

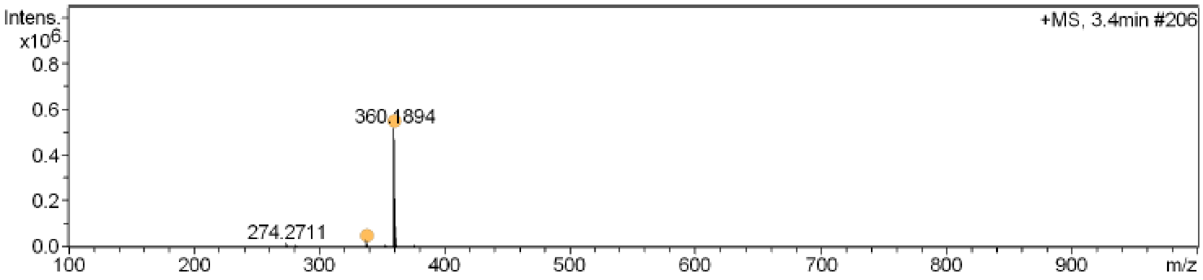

Meas. m/z	#	Ion Formula	m/z	err [ppm]	mSigma	# Sigma	Score	rdb	e⁻ Conf	N-Rule
338.2064	1	C17H28N3O4	338.2074	-3.1	16.8	1	100.00	5.5	even	ok
360.1894	1	C17H27N3NaO4	360.1894	-0.1	3.5	1	100.00	5.5	even	ok

Figure S76. *tert*-Butyl 4-[1-ethyl-4-(methoxycarbonyl)-1*H*-pyrazol-3-yl]piperidine-1-carboxylate (8). HRMS (ESI-TOF).

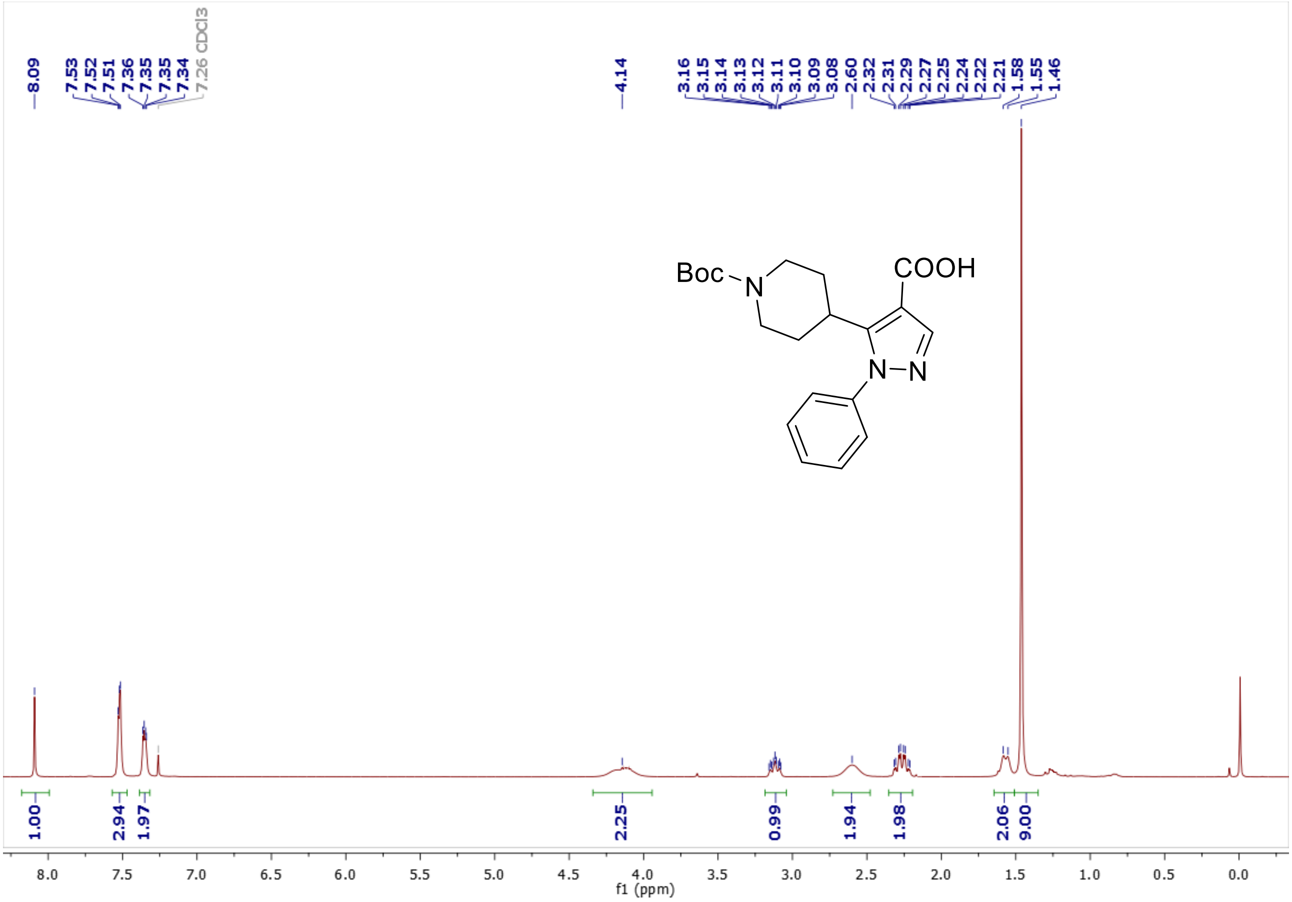

Figure S77. 5-[1-(*tert*-Butoxycarbonyl)piperidin-4-yl]-1-phenyl-1*H*-pyrazole-4-carboxylic acid (9a). ^{1}H NMR spectrum (400 MHz, $CDCl_3$).

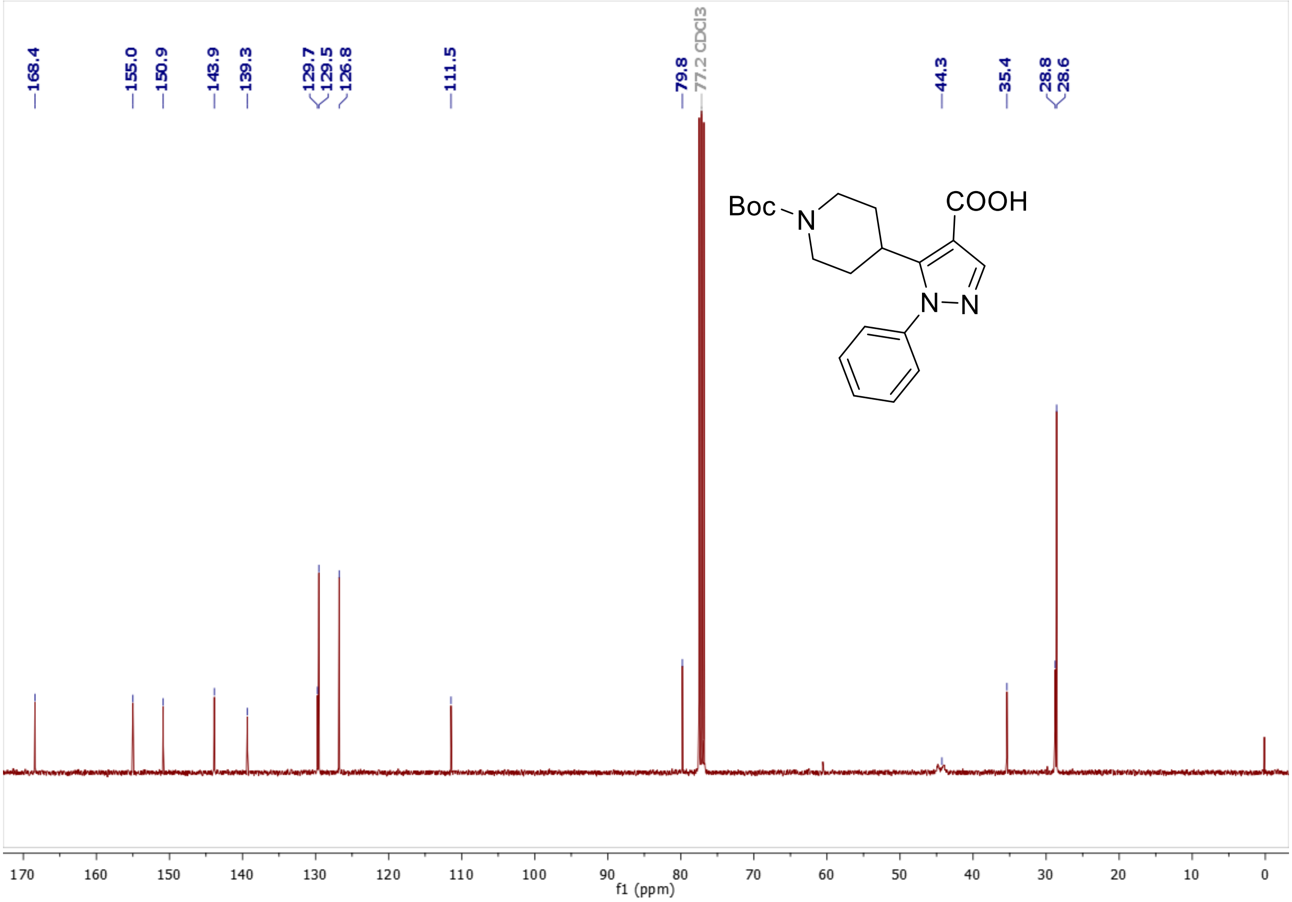

Figure S78. 5-[1-(*tert*-Butoxycarbonyl)piperidin-4-yl]-1-phenyl-1*H*-pyrazole-4-carboxylic acid (9a). ^{13}C NMR spectrum (101 MHz, $CDCl_3$).

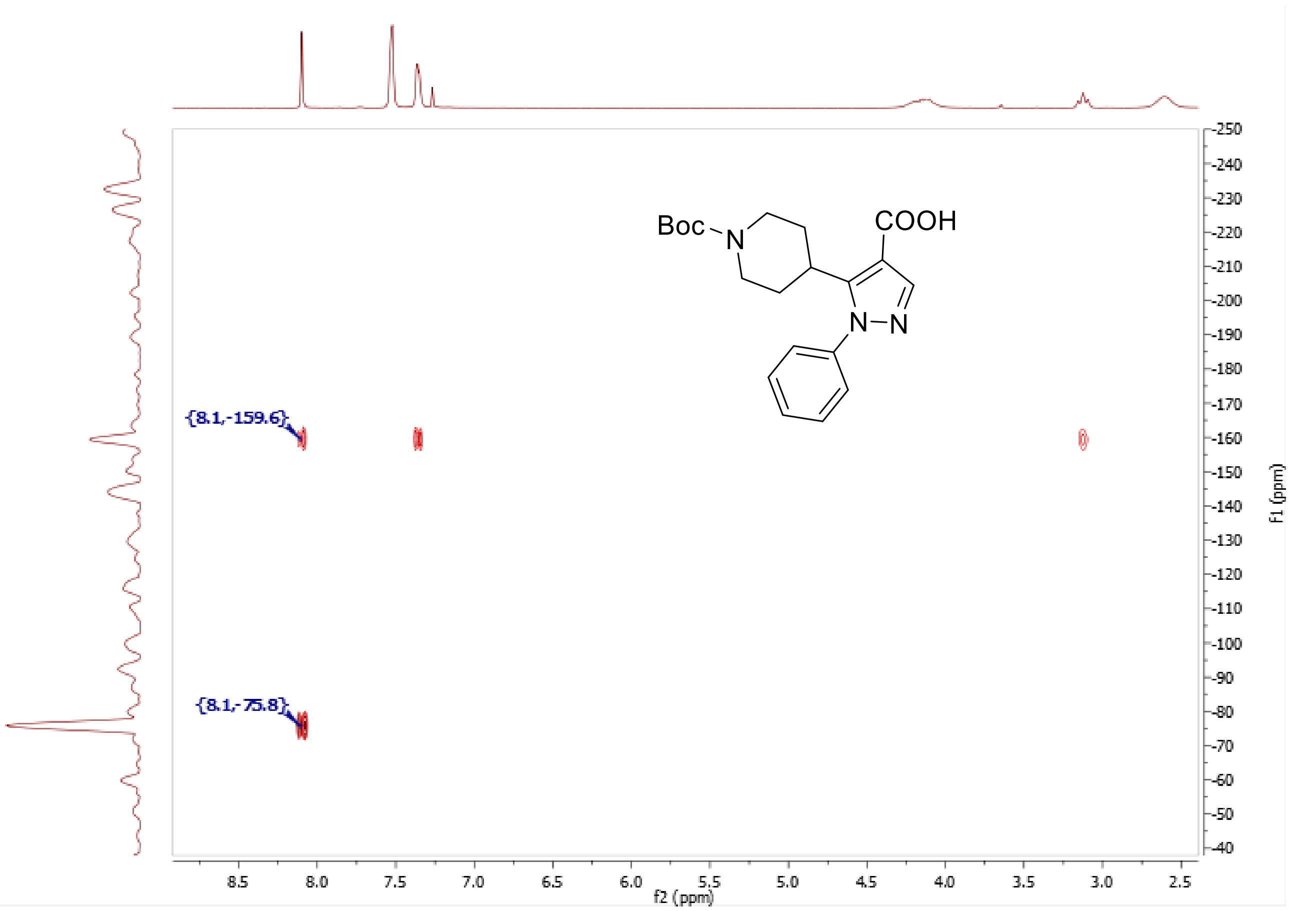

Figure S79. 5-[1-(*tert*-Butoxycarbonyl)piperidin-4-yl]-1-phenyl-1*H*-pyrazole-4-carboxylic acid (9a). ^{1}H-^{15}N HMBC spectrum (41 MHz, $CDCl_3$).

Compound Spectrum SmartFormula Report

Analysis Info

Analysis Name	D:\Data\GMP-121.d	Acquisition Date	2/4/2021 9:41:19 PM
Method	DirectInfusion_TuneLow_pos.m	Operator	hplc
Sample Name	GMP-121	Instrument	micrOTOF-Q III 8228888.20448
Comment	AB		

Acquisition Parameter

Source Type	ESI	Ion Polarity	Positive	Set Nebulizer	0.4 Bar
Focus	Not active	Set Capillary	4500 V	Set Dry Heater	180 °C
Scan Begin	50 m/z	Set End Plate Offset	-500 V	Set Dry Gas	4.0 l/min
Scan End	1000 m/z	Set Collision Cell RF	140.0 Vpp	Set Divert Valve	Waste

#	RT [min]	Area	Int. Type	I	S/N	Chromatogram	Max. m/z	FWHM [min]
n.a.	3.0	n.a.	Single spectrum	n.a.	n.a.	n.a.	394.1738	n.a.

+MS, 3.0min #180

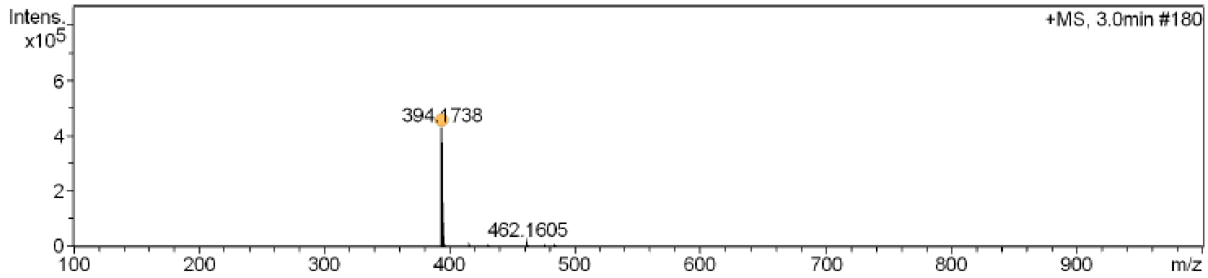

Meas. m/z	#	Ion Formula	m/z	err [ppm]	mSigma	# Sigma	Score	rdb	e⁻ Conf	N-Rule
394.1738	1	C20H25N3NaO4	394.1737	-0.2	1.2	1	100.00	9.5	even	ok

Figure S80. 5-[1-(*tert*-Butoxycarbonyl)piperidin-4-yl]-1-phenyl-1*H*-pyrazole-4-carboxylic acid (9a). HRMS (ESI-TOF).

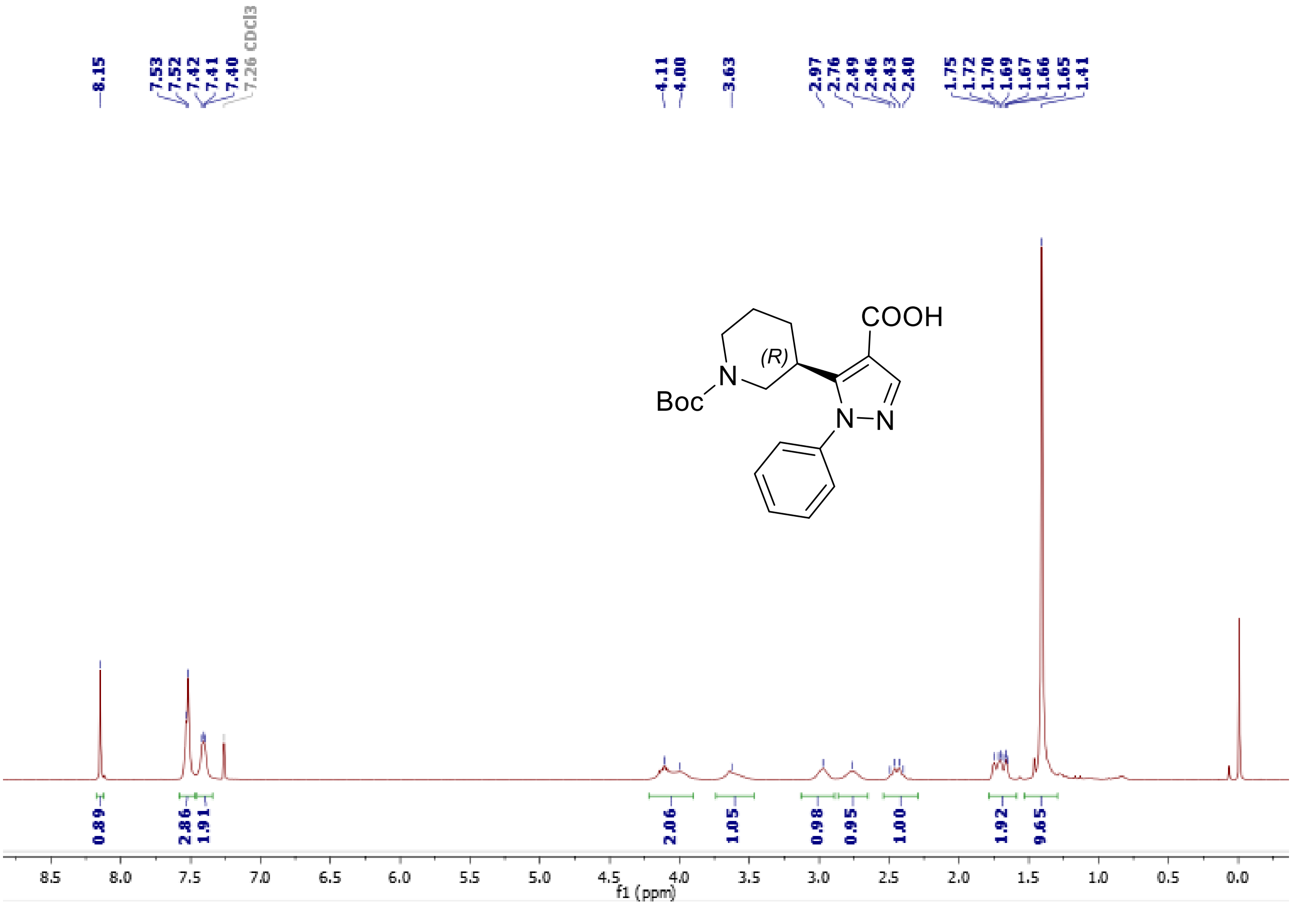

Figure S81. 5-[(3*R*)-1-(*tert*-Butoxycarbonyl)piperidin-3-yl]-1-phenyl-1*H*-pyrazole-4-carboxylic acid (9b). ^{1}H NMR spectrum (400 MHz, $CDCl_3$).

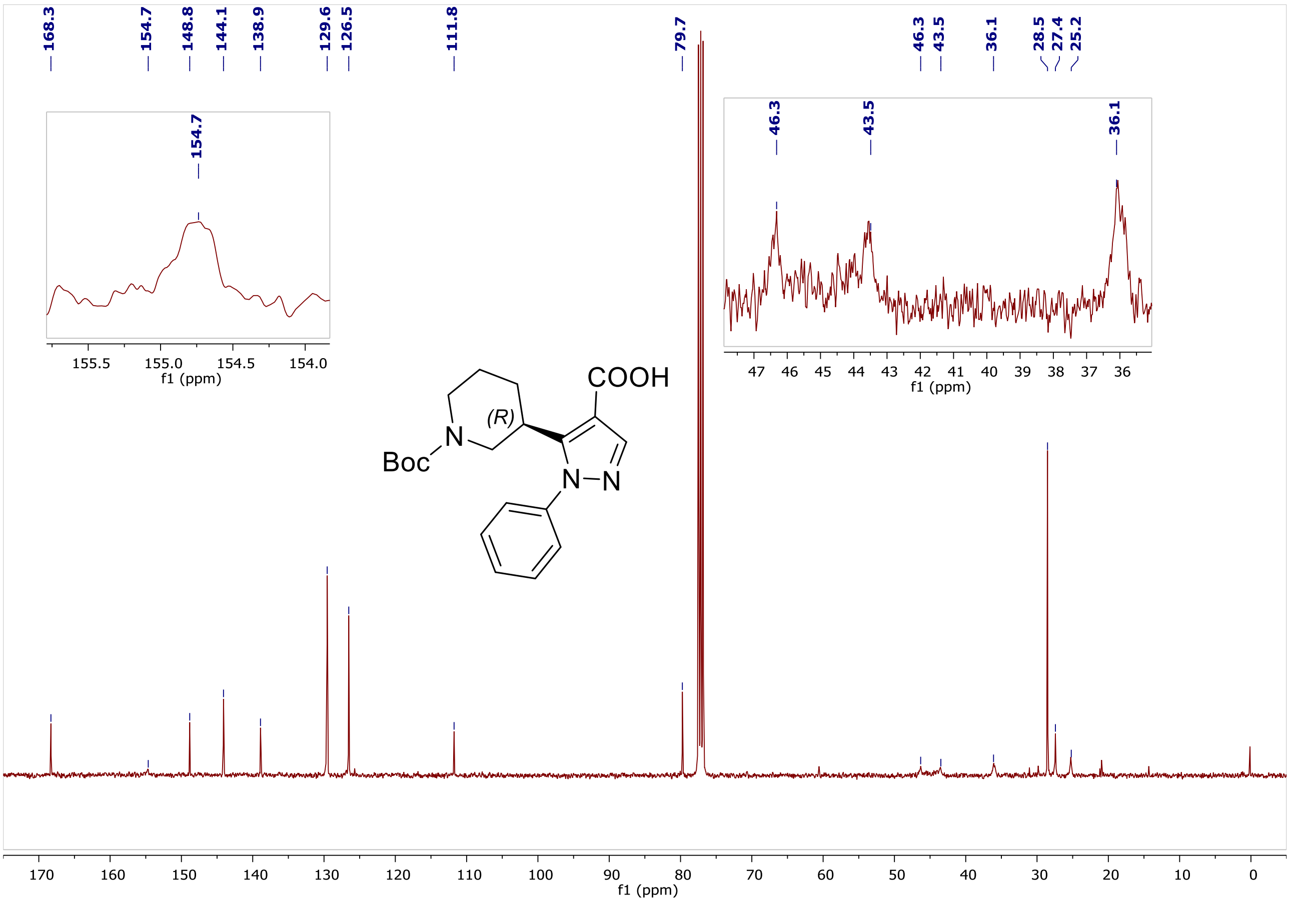

Figure S82. 5-[(3*R*)-1-(*tert*-Butoxycarbonyl)piperidin-3-yl]-1-phenyl-1*H*-pyrazole-4-carboxylic acid (9b). ^{13}C NMR spectrum (101 MHz, $CDCl_3$).

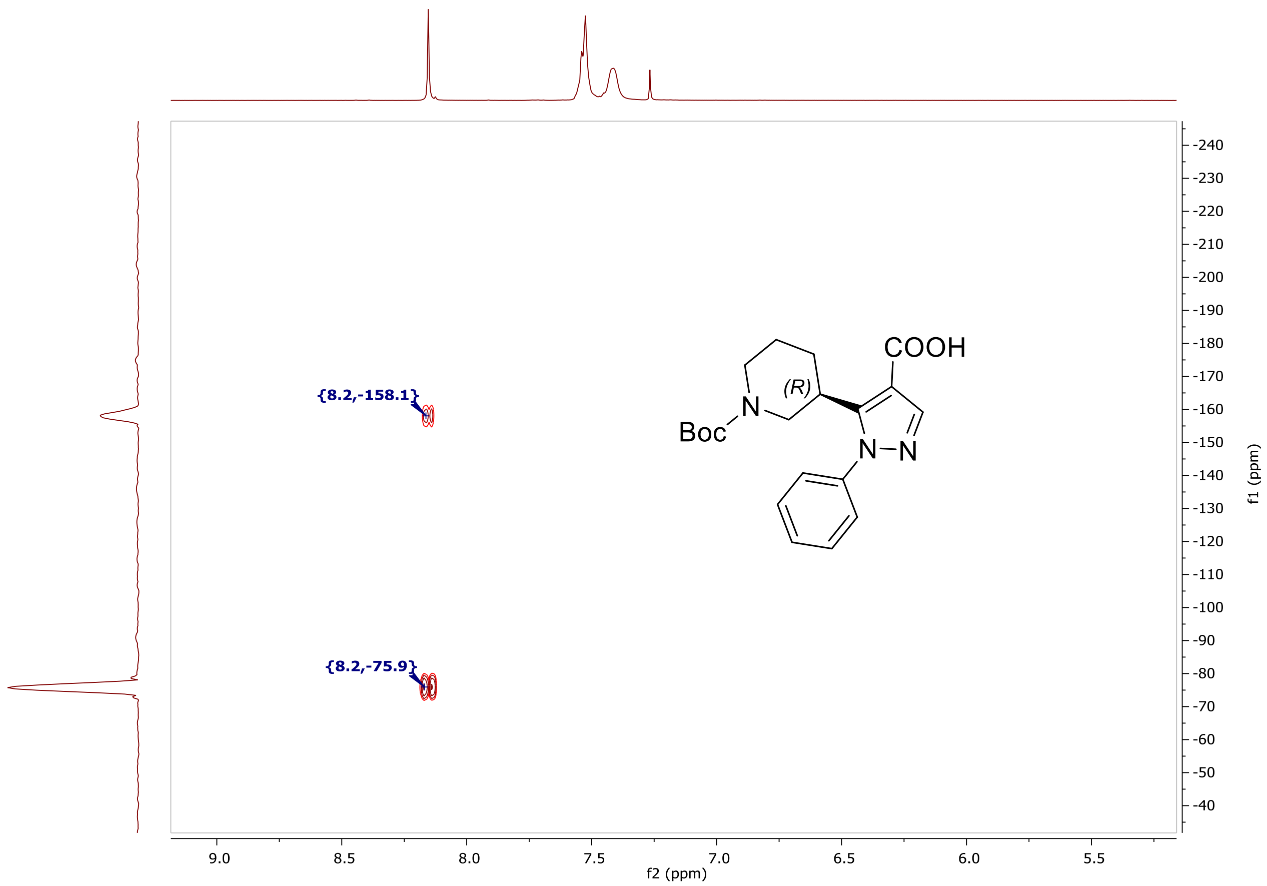

Figure S83. 5-[(3*R*)-1-(*tert*-Butoxycarbonyl)piperidin-3-yl]-1-phenyl-1*H*-pyrazole-4-carboxylic acid (9b). ^{1}H-^{15}N HMBC spectrum (41 MHz, $CDCl_3$).

Compound Spectrum SmartFormula Report

Analysis Info

Analysis Name	D:\Data\GMP-124.d	Acquisition Date	2/4/2021 9:18:32 PM
Method	DirectInfusion_TuneLow_pos.m	Operator	hplc
Sample Name	GMP-124	Instrument	micrOTOF-Q III 8228888.20448
Comment	AB		

Acquisition Parameter

Source Type	ESI	Ion Polarity	Positive	Set Nebulizer	0.4 Bar
Focus	Not active	Set Capillary	4500 V	Set Dry Heater	180 °C
Scan Begin	50 m/z	Set End Plate Offset	-500 V	Set Dry Gas	4.0 l/min
Scan End	1000 m/z	Set Collision Cell RF	140.0 Vpp	Set Divert Valve	Waste

#	RT [min]	Area	Int. Type	I	S/N	Chromatogram	Max. m/z	FWHM [min]
n.a.	4.0	n.a.	Single spectrum	n.a.	n.a.	n.a.	394.1738	n.a.

+MS, 4.0min #238

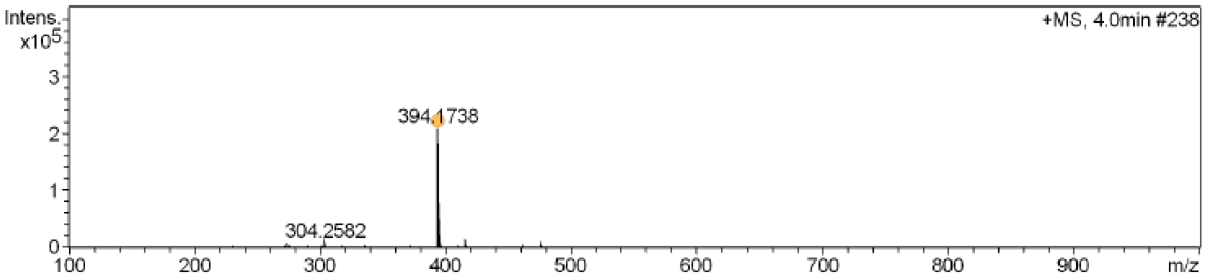

Meas. m/z	#	Ion Formula	m/z	err [ppm]	mSigma	# Sigma	Score	rdb	e⁻ Conf	N-Rule
394.1738	1	C20H25N3NaO4	394.1737	0.1	21.6	3	100.00	9.5	even	ok

Figure S84. 5-[(3*R*)-1-(*tert*-Butoxycarbonyl)piperidin-3-yl]-1-phenyl-1*H*-pyrazole-4-carboxylic acid (9b). HRMS (ESI-TOF).

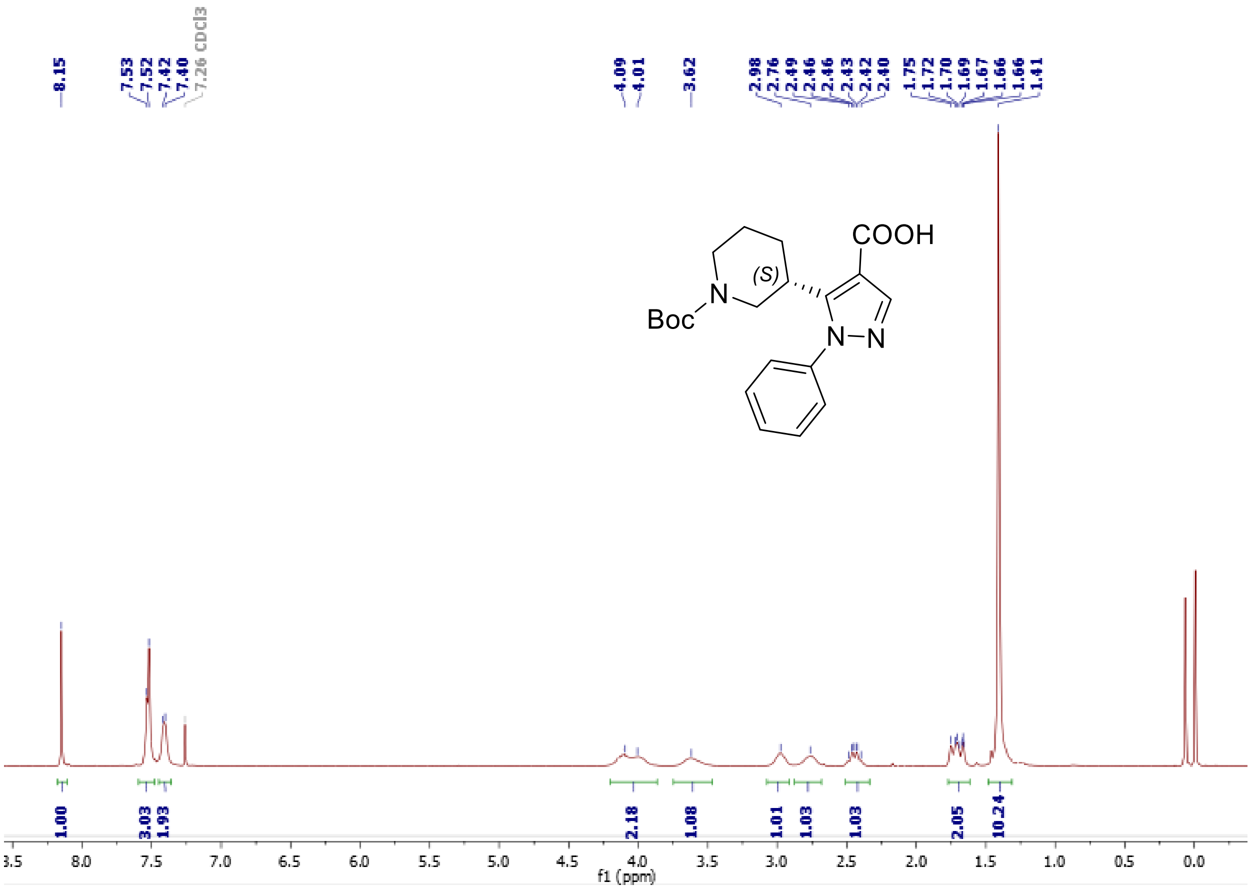

Figure S85. 5-[(3*S*)-1-(*tert*-Butoxycarbonyl)piperidin-3-yl]-1-phenyl-1*H*-pyrazole-4-carboxylic acid (9c). ^{1}H NMR spectrum (400 MHz, $CDCl_3$).

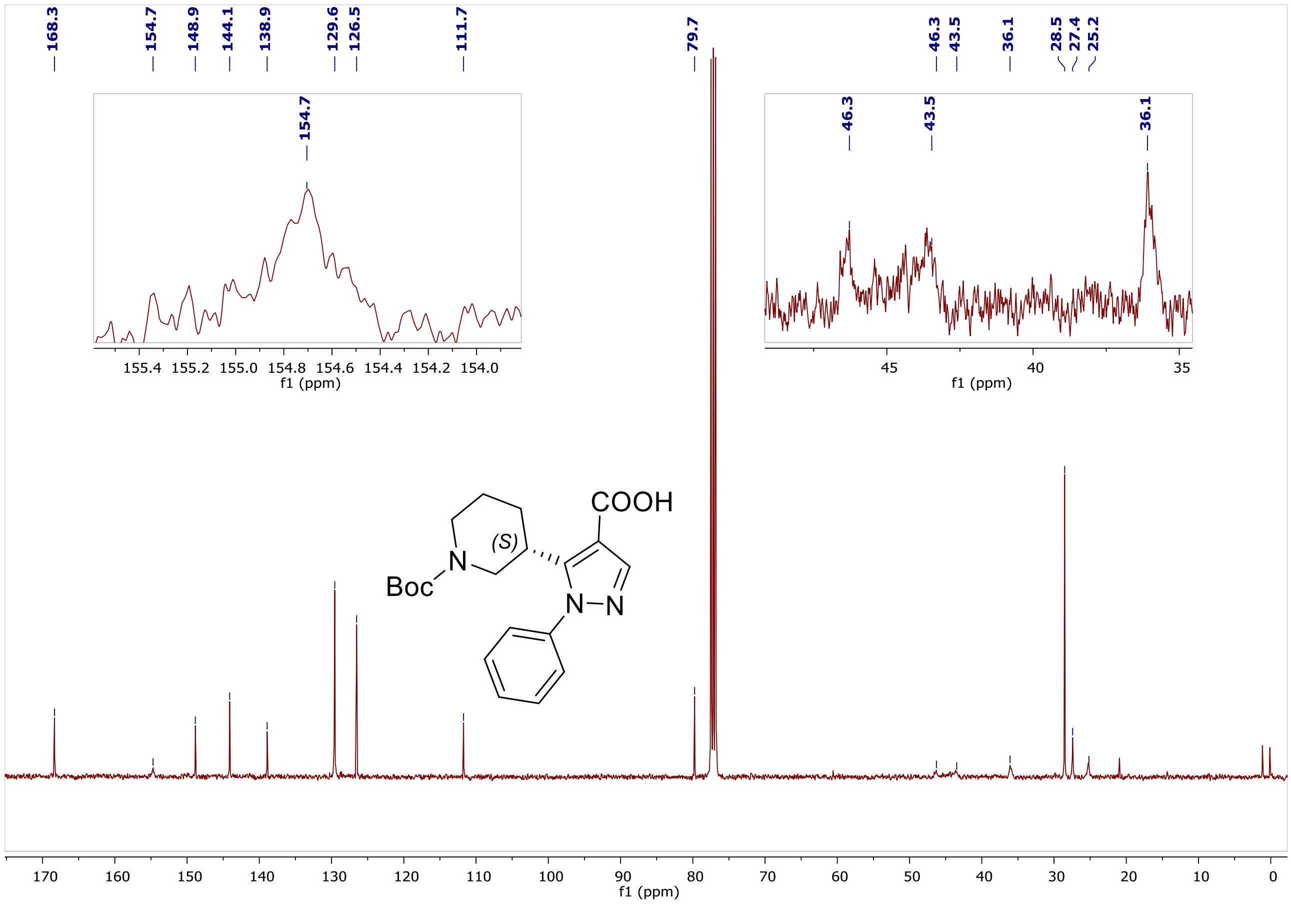

Figure S86. 5-[(3*S*)-1-(*tert*-Butoxycarbonyl)piperidin-3-yl]-1-phenyl-1*H*-pyrazole-4-carboxylic acid (9c). ^{13}C NMR spectrum (101 MHz, $CDCl_3$).

Compound Spectrum SmartFormula Report

Analysis Info

Analysis Name	D:\Data\GMP-152.d	Acquisition Date	2/4/2021 2:07:00 PM
Method	DirectInfusion_TuneLow_pos.m	Operator	hplc
Sample Name	GMP-152	Instrument	micrOTOF-Q III 8228888.20448
Comment	AB		

Acquisition Parameter

Source Type	ESI	Ion Polarity	Positive	Set Nebulizer	0.4 Bar
Focus	Not active	Set Capillary	4500 V	Set Dry Heater	180 °C
Scan Begin	50 m/z	Set End Plate Offset	-500 V	Set Dry Gas	4.0 l/min
Scan End	1000 m/z	Set Collision Cell RF	140.0 Vpp	Set Divert Valve	Waste

#	RT [min]	Area	Int. Type	I	S/N	Chromatogram	Max. m/z	FWHM [min]
n.a.	8.2	n.a.	Single spectrum	n.a.	n.a.	n.a.	394.1739	n.a.

+MS, 8.2min #491

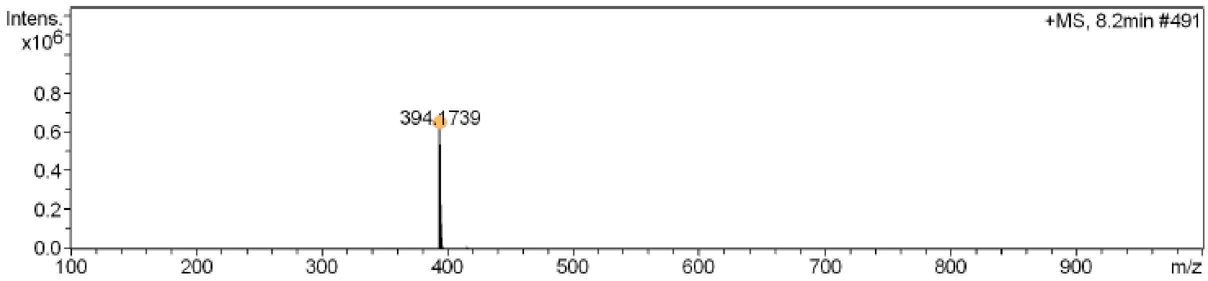

Meas. m/z	#	Ion Formula	m/z	err [ppm]	mSigma	# Sigma	Score	rdb	e⁻ Conf	N-Rule
394.1739	1	C20H25N3NaO4	394.1737	-0.3	7.5	1	100.00	9.5	even	ok

Figure S87. 5-[(3*S*)-1-(*tert*-Butoxycarbonyl)piperidin-3-yl]-1-phenyl-1*H*-pyrazole-4-carboxylic acid (9c). HRMS (ESI-TOF).

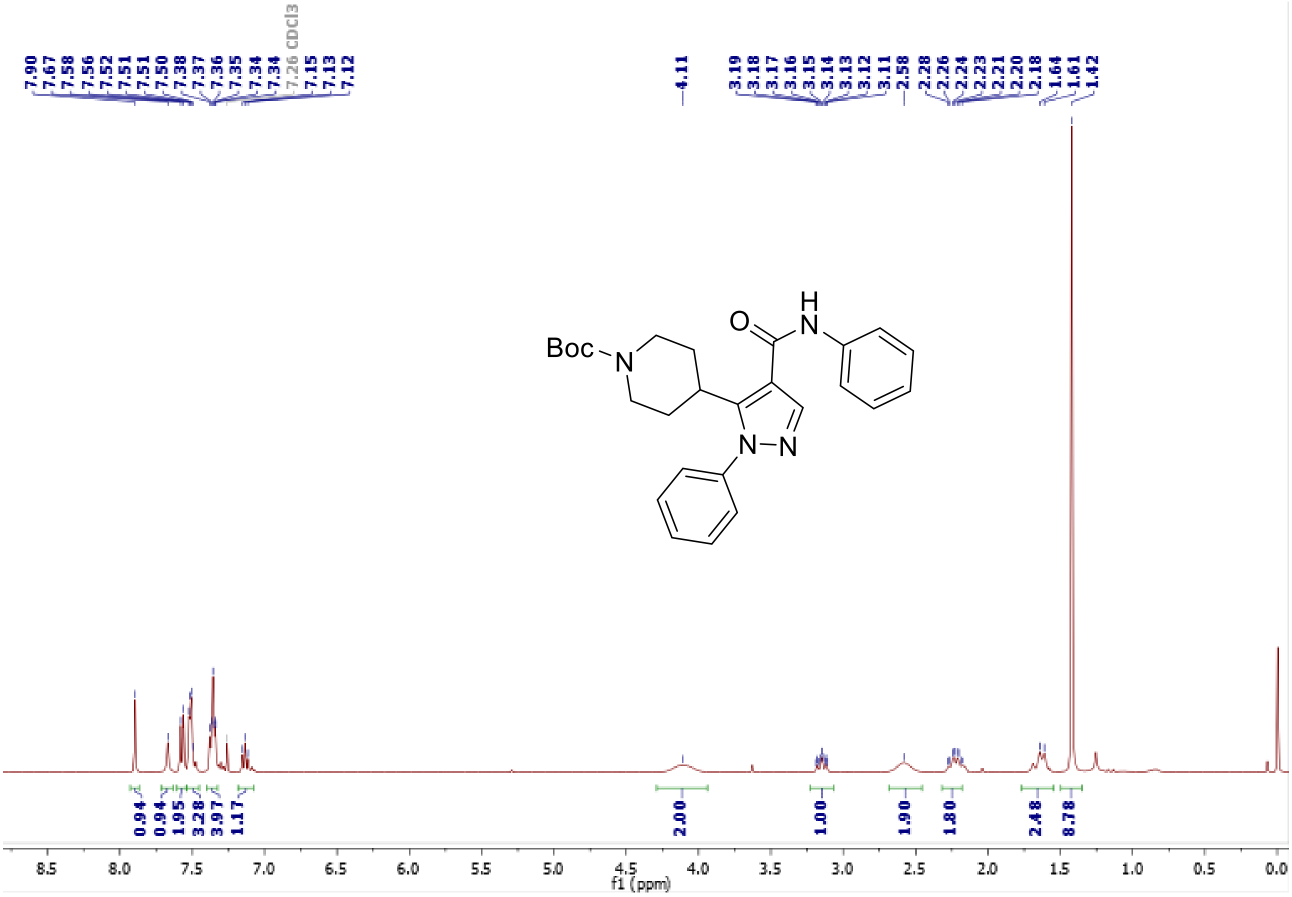

Figure S88. ***tert*-Butyl 4-[1-phenyl-4-(phenylcarbamoyl)-1*H*-pyrazol-5-yl]piperidine-1-carboxylate (10a). ^{1}H NMR spectrum (400 MHz, CDCl$_3$).**

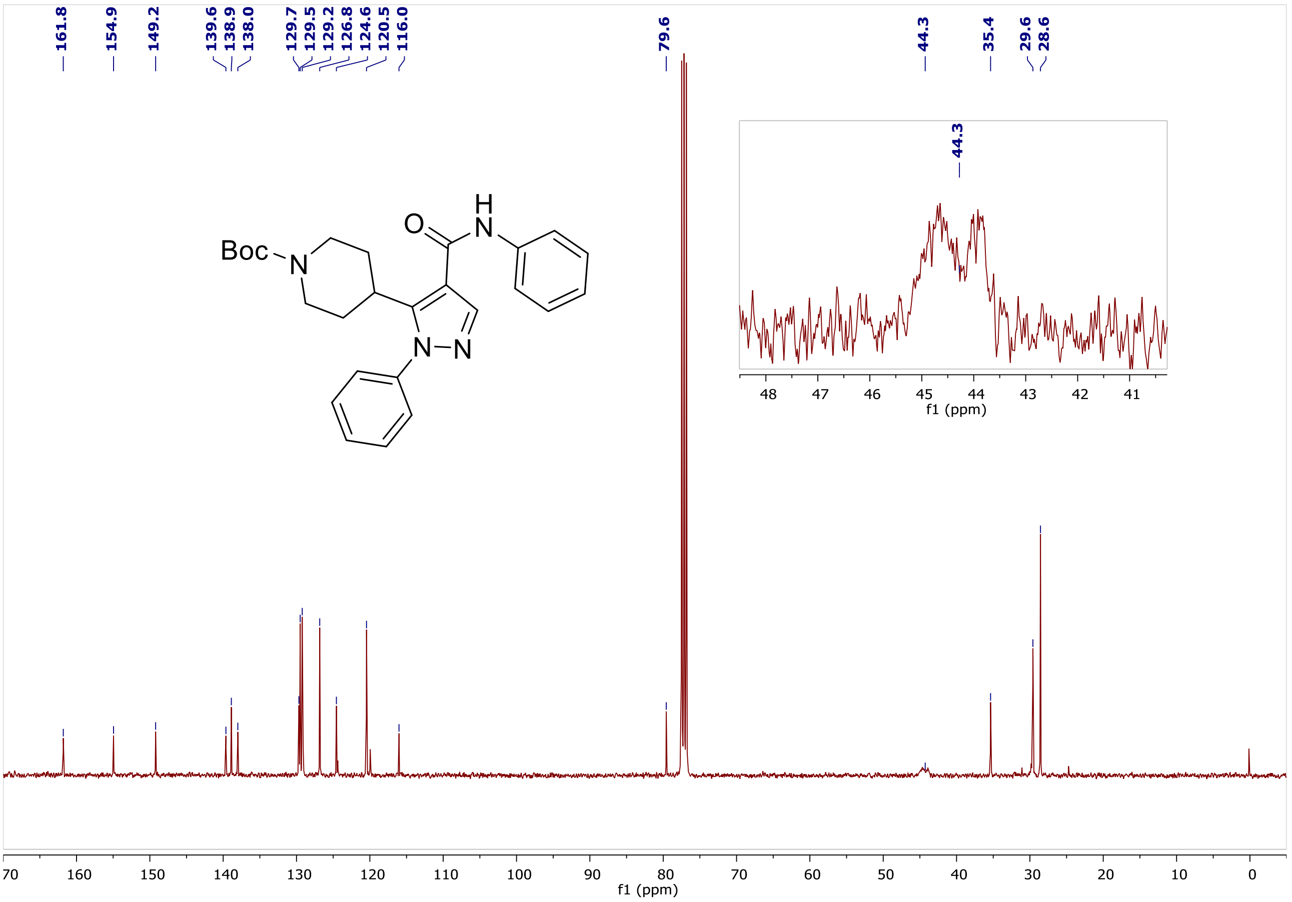

Figure S89. ***tert*-Butyl 4-[1-phenyl-4-(phenylcarbamoyl)-1*H*-pyrazol-5-yl]piperidine-1-carboxylate (10a). ^{13}C NMR spectrum (101 MHz, CDCl$_3$).**

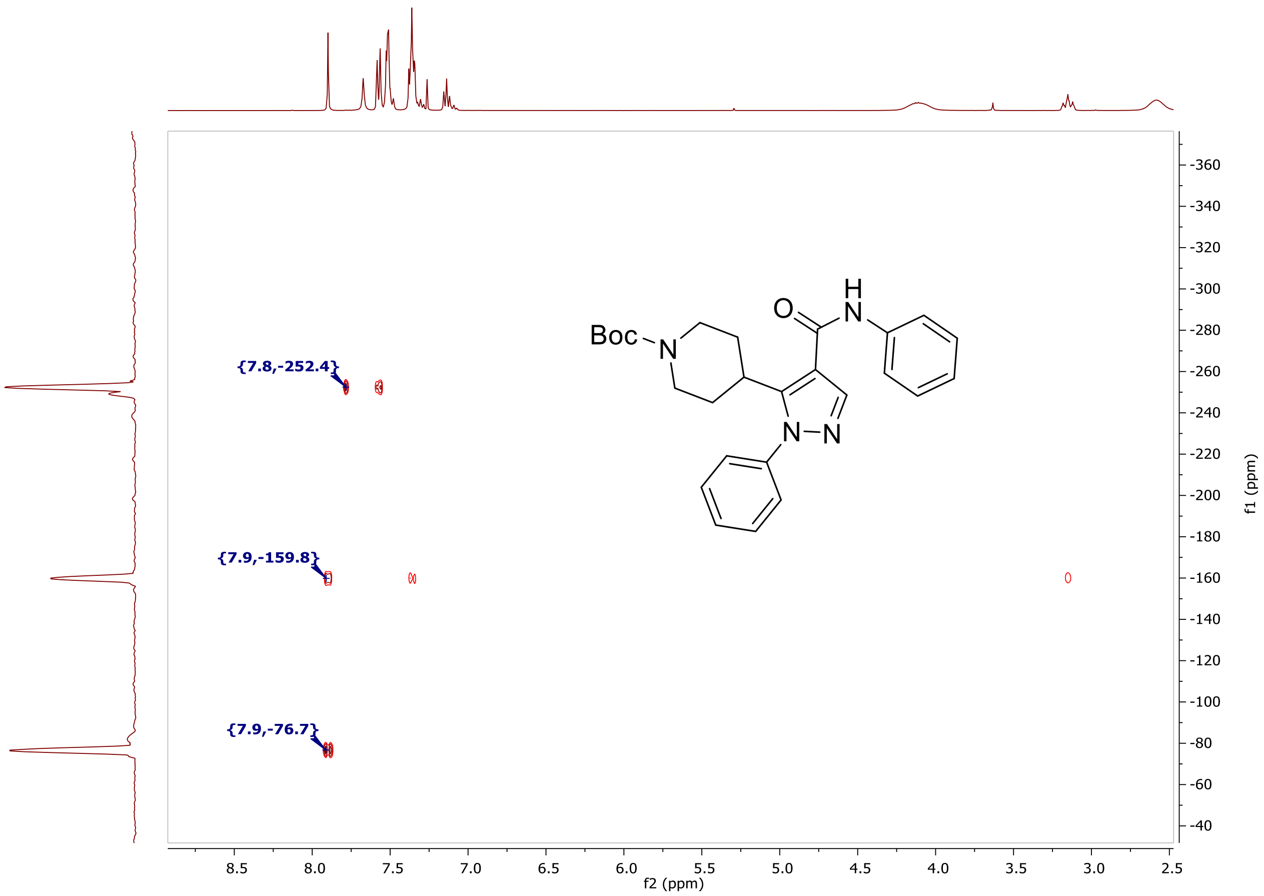

Figure S90. ***tert*-Butyl 4-[1-phenyl-4-(phenylcarbamoyl)-1*H*-pyrazol-5-yl]piperidine-1-carboxylate (10a). ^{1}H-^{15}N HMBC spectrum (41 MHz, $CDCl_3$).**

Compound Spectrum SmartFormula Report

Analysis Info

Analysis Name	D:\Data\GMP-126.d	Acquisition Date	2/4/2021 9:31:08 PM
Method	DirectInfusion_TuneLow_pos.m	Operator	hplc
Sample Name	GMP-126	Instrument	micrOTOF-Q III 8228888.20448
Comment	AB		

Acquisition Parameter

Source Type	ESI	Ion Polarity	Positive	Set Nebulizer	0.4 Bar
Focus	Not active	Set Capillary	4500 V	Set Dry Heater	180 °C
Scan Begin	50 m/z	Set End Plate Offset	-500 V	Set Dry Gas	4.0 l/min
Scan End	1000 m/z	Set Collision Cell RF	140.0 Vpp	Set Divert Valve	Waste

#	RT [min]	Area	Int. Type	I	S/N	Chromatogram	Max. m/z	FWHM [min]
n.a.	2.0	n.a.	Single spectrum	n.a.	n.a.	n.a.	469.2209	n.a.

+MS, 2.0min #118

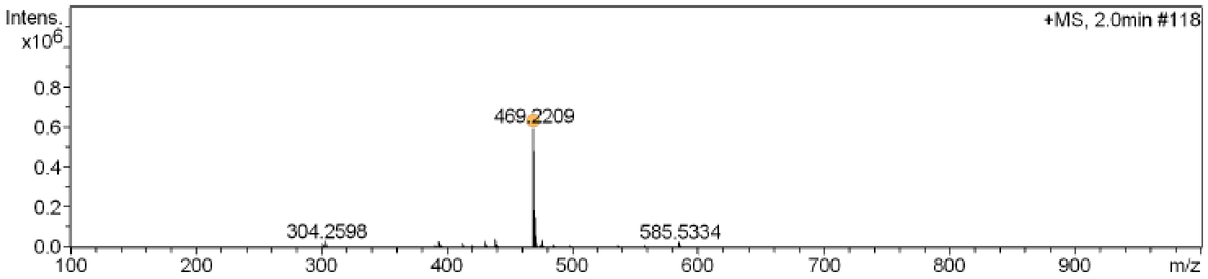

Meas. m/z	#	Ion Formula	m/z	err [ppm]	mSigma	# Sigma	Score	rdb	e⁻ Conf	N-Rule
469.2209	1	C26H30N4NaO3	469.2210	-0.2	3.0	1	100.00	13.5	even	ok

Figure S91. *tert*-Butyl 4-[1-phenyl-4-(phenylcarbamoyl)-1*H*-pyrazol-5-yl]piperidine-1-carboxylate (10a). HRMS (ESI-TOF).

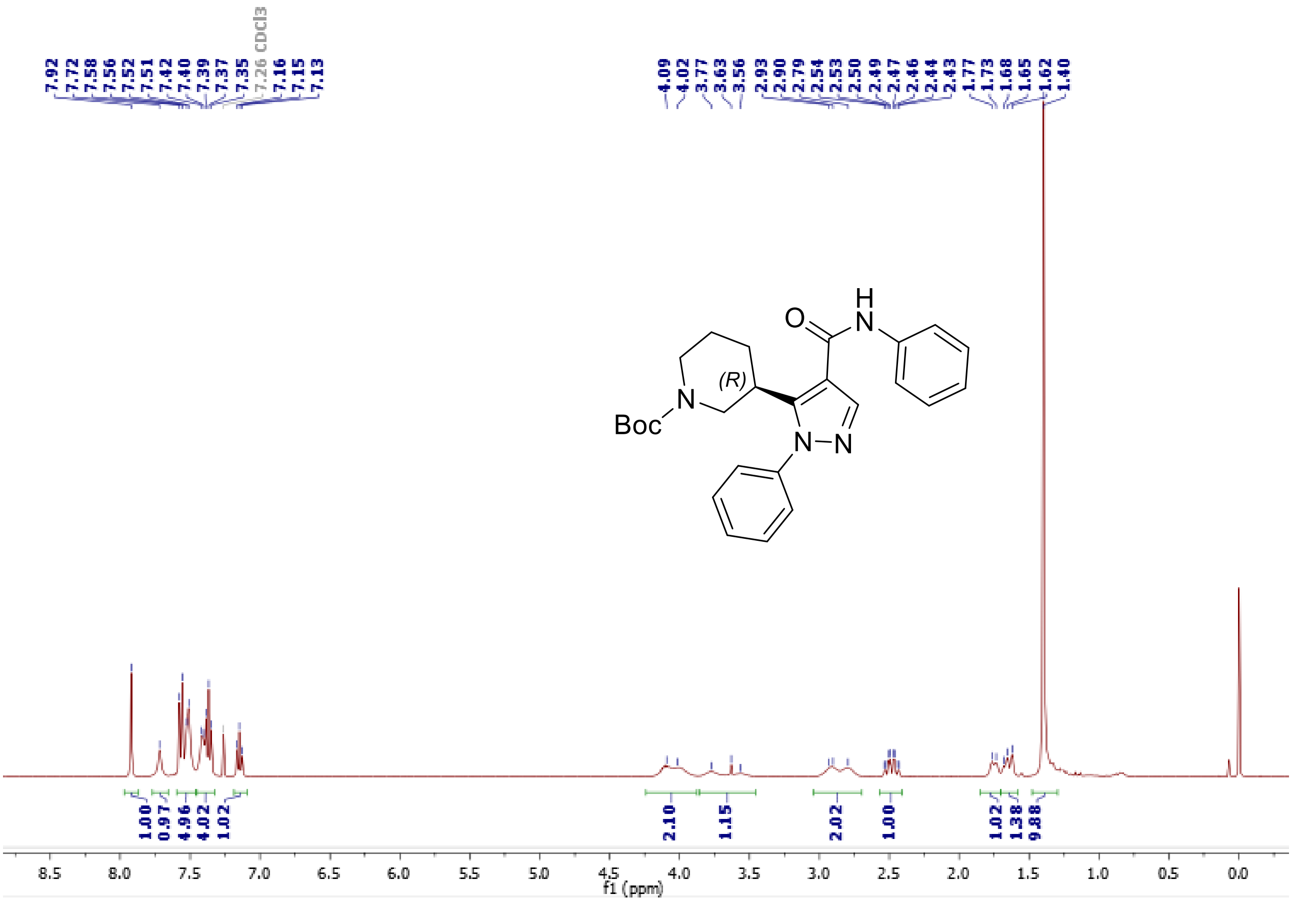

Figure S92. ***tert*-Butyl (3*R*)-3-[1-phenyl-4-(phenylcarbamoyl)-1*H*-pyrazol-5-yl]piperidine-1-carboxylate (10b). ^{1}H NMR spectrum (400 MHz, CDCl$_3$).**

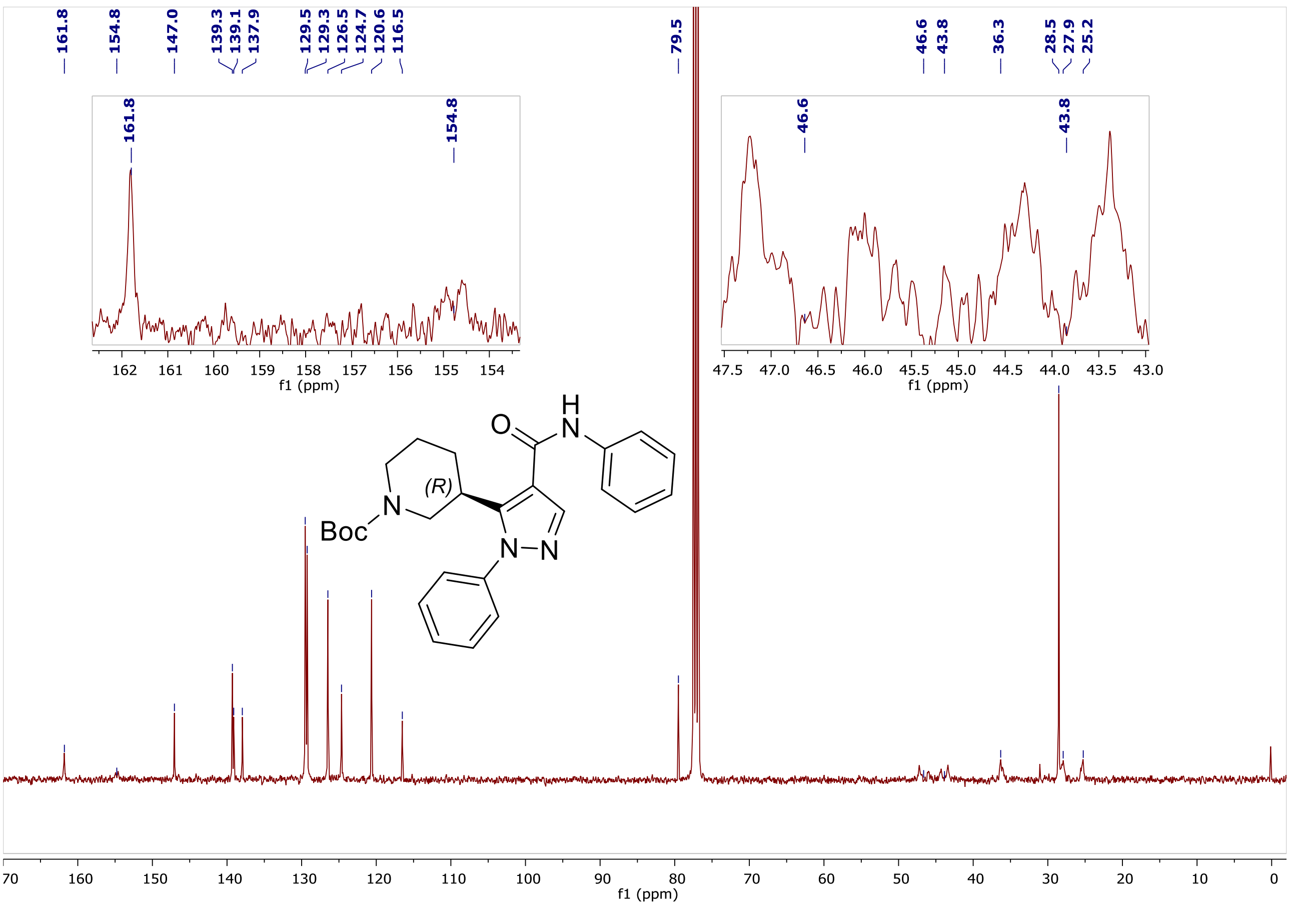

Figure S93. ***tert*-Butyl (3*R*)-3-[1-phenyl-4-(phenylcarbamoyl)-1*H*-pyrazol-5-yl]piperidine-1-carboxylate (10b). ^{13}C NMR spectrum (101 MHz, CDCl$_3$).**

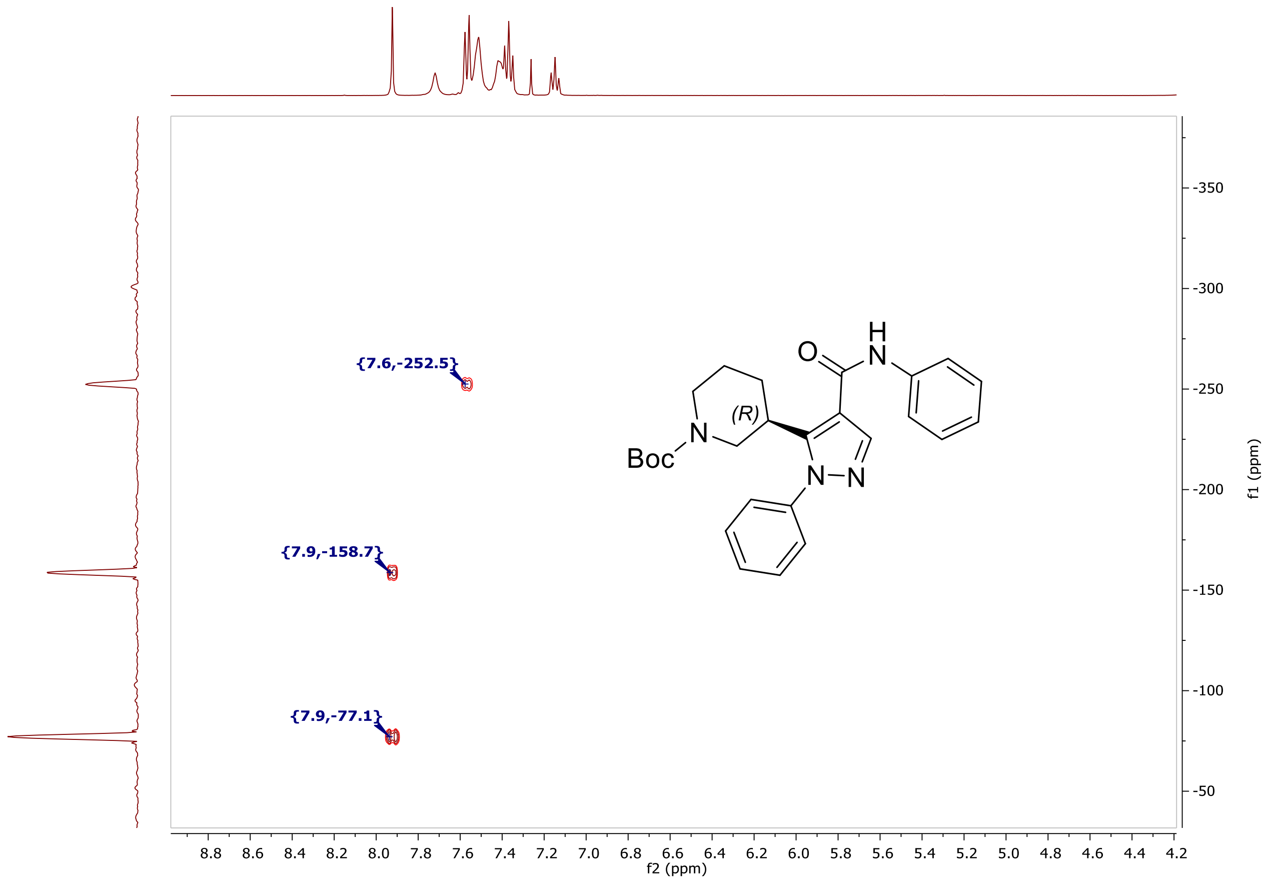

Figure S94. *tert*-Butyl (3*R*)-3-[1-phenyl-4-(phenylcarbamoyl)-1*H*-pyrazol-5-yl]piperidine-1-carboxylate (10b). ^{1}H-^{15}N HMBC spectrum (41 MHz, $CDCl_3$).

Compound Spectrum SmartFormula Report

Analysis Info

Analysis Name	D:\Data\GMP-129.d	Acquisition Date	2/4/2021 9:00:51 PM
Method	DirectInfusion_TuneLow_pos.m	Operator	hplc
Sample Name	GMP-129	Instrument	micrOTOF-Q III 8228888.20448
Comment	AB		

Acquisition Parameter

Source Type	ESI	Ion Polarity	Positive	Set Nebulizer	0.4 Bar
Focus	Not active	Set Capillary	4500 V	Set Dry Heater	180 °C
Scan Begin	50 m/z	Set End Plate Offset	-500 V	Set Dry Gas	4.0 l/min
Scan End	1000 m/z	Set Collision Cell RF	140.0 Vpp	Set Divert Valve	Waste

#	RT [min]	Area	Int. Type	I	S/N	Chromatogram	Max. m/z	FWHM [min]
n.a.	3.1	n.a.	Single spectrum	n.a.	n.a.	n.a.	469.2216	n.a.

+MS, 3.1min #180

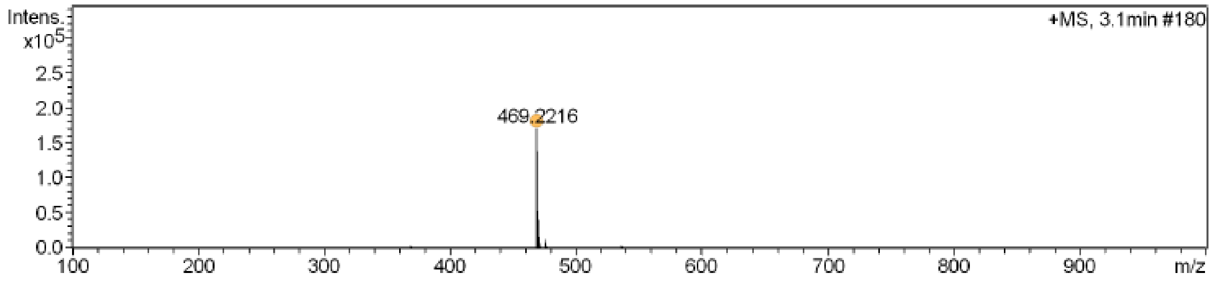

Meas. m/z	#	Ion Formula	m/z	err [ppm]	mSigma	# Sigma	Score	rdb	e⁻ Conf	N-Rule
469.2216	1	C26H30N4NaO3	469.2210	1.3	4.9	1	100.00	13.5	even	ok

Figure S95. *tert*-Butyl (3*R*)-3-[1-phenyl-4-(phenylcarbamoyl)-1*H*-pyrazol-5-yl]piperidine-1-carboxylate (10b). HRMS (ESI-TOF).

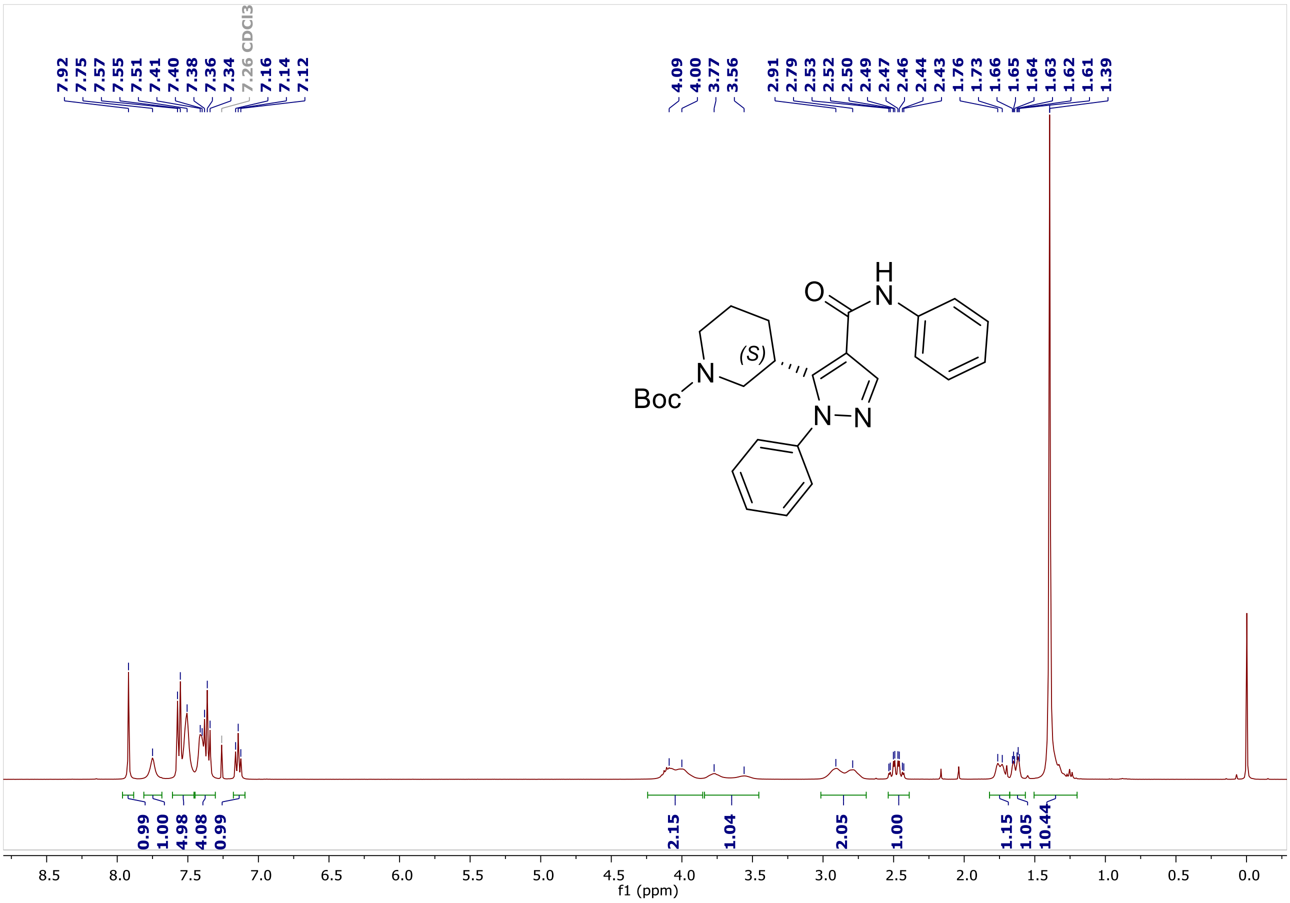

Figure S96. ***tert*-Butyl (3*S*)-3-[1-phenyl-4-(phenylcarbamoyl)-1*H*-pyrazol-5-yl]piperidine-1-carboxylate (10c). ¹H NMR spectrum (400 MHz, CDCl₃).**

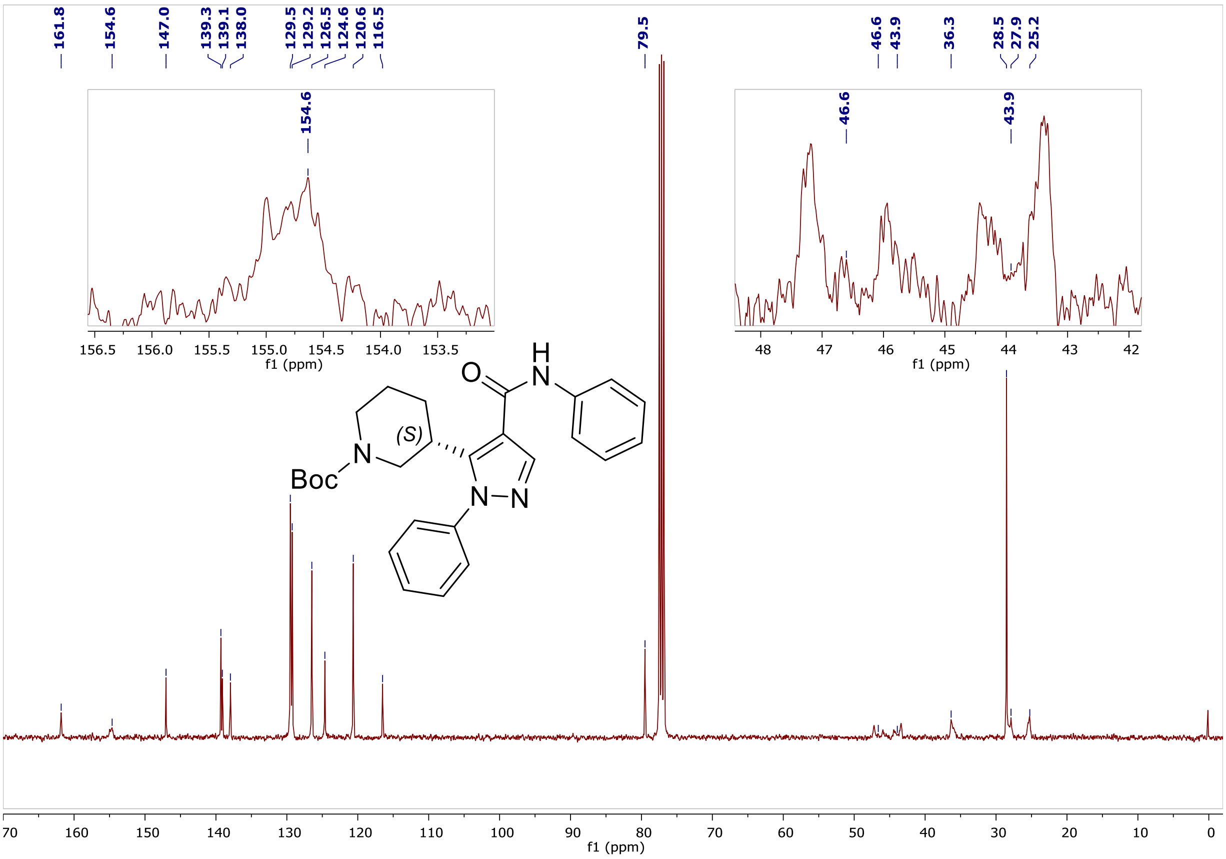

Figure S97. ***tert*-Butyl (3*S*)-3-[1-phenyl-4-(phenylcarbamoyl)-1*H*-pyrazol-5-yl]piperidine-1-carboxylate (10c). ¹³C NMR spectrum (101 MHz, CDCl₃).**

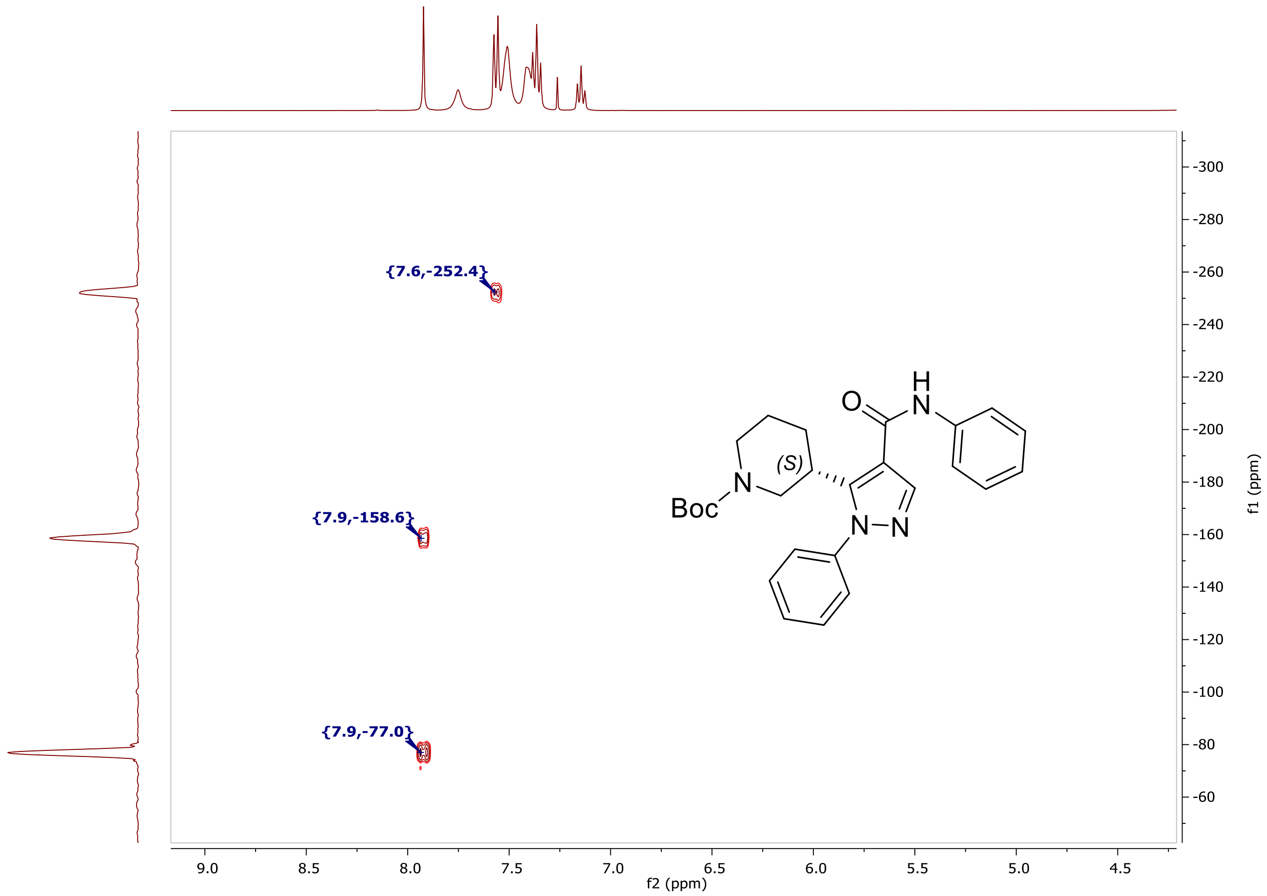

Figure S98. ***tert*****-Butyl (3*S*)-3-[1-phenyl-4-(phenylcarbamoyl)-1*H*-pyrazol-5-yl]piperidine-1-carboxylate (10c).** ^{1}**H-**^{15}**N HMBC spectrum (41 MHz,** $CDCl_3$**).**

Compound Spectrum SmartFormula Report

Analysis Info

Analysis Name	D:\Data\GMP-155.d	Acquisition Date	2/4/2021 1:42:18 PM
Method	DirectInfusion_TuneLow_pos.m	Operator	hplc
Sample Name	GMP-155	Instrument	micrOTOF-Q III 8228888.20448
Comment	AB		

Acquisition Parameter

Source Type	ESI	Ion Polarity	Positive	Set Nebulizer	0.4 Bar
Focus	Not active	Set Capillary	4500 V	Set Dry Heater	180 °C
Scan Begin	50 m/z	Set End Plate Offset	-500 V	Set Dry Gas	4.0 l/min
Scan End	1000 m/z	Set Collision Cell RF	140.0 Vpp	Set Divert Valve	Waste

#	RT [min]	Area	Int. Type	I	S/N	Chromatogram	Max. m/z	FWHM [min]
n.a.	3.4	n.a.	Single spectrum	n.a.	n.a.	n.a.	469.2210	n.a.

+MS, 3.4min #200

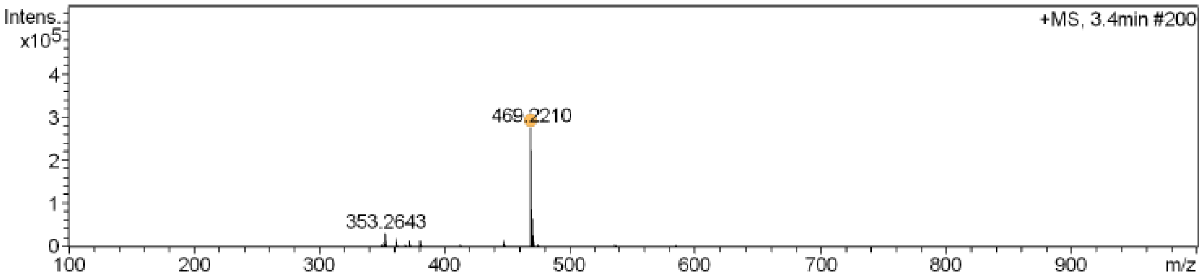

Meas. m/z	#	Ion Formula	m/z	err [ppm]	mSigma	# Sigma	Score	rdb	e⁻ Conf	N-Rule
469.2210	1	C26H30N4NaO3	469.2210	-0.1	5.0	1	100.00	13.5	even	ok

Figure S99. *tert*-Butyl (3*S*)-3-[1-phenyl-4-(phenylcarbamoyl)-1*H*-pyrazol-5-yl]piperidine-1-carboxylate (10c). HRMS (ESI-TOF).
